# A Review on the Toxicity and Non-Target Effects of Macrocyclic Lactones in Terrestrial and Aquatic Environments

**DOI:** 10.2174/138920112800399257

**Published:** 2012-05

**Authors:** Jean-Pierre Lumaret, Faiek Errouissi, Kevin Floate, Jörg Römbke, Keith Wardhaugh

**Affiliations:** 1 UMR 5175 CEFE, Zoogéographie, Université Montpellier III, 34199 Montpellier cedex 5, France; 2 UR Biodiversité et Biologie des Populations, ISSBAT, 9 ave Zohair Essefi, 1007, Tunis, Tunisia; 3 Agriculture and Agri-Food Canada, Lethbridge Research Centre, Lethbridge, Alberta, Canada, T1J 4B1; 4 ECT Oekotoxikologie GmbH, Böttgerstrasse 2-14, D-65439 Flörsheim, Germany; 5 11 Deane Street, Yarralumla, Canberra, ACT 2600, Australia

**Keywords:** Abamectin, doramectin, ecotoxicology, endectocides, eprinomectin, emamectin benzoate, ivermectin, moxidectin (milbemycin), spinosad, veterinary pharmaceuticals.

## Abstract

The avermectins, milbemycins and spinosyns are collectively referred to as macrocyclic lactones (MLs) which comprise several classes of chemicals derived from cultures of soil micro-organisms. These compounds are extensively and increasingly used in veterinary medicine and agriculture. Due to their potential effects on non-target organisms, large amounts of information on their impact in the environment has been compiled in recent years, mainly caused by legal requirements related to their marketing authorization or registration. The main objective of this paper is to critically review the present knowledge about the acute and chronic ecotoxicological effects of MLs on organisms, mainly invertebrates, in the terrestrial and aquatic environment. Detailed information is presented on the mode-of-action as well as the ecotoxicity of the most important compounds representing the three groups of MLs. This information, based on more than 360 references, is mainly provided in nine tables, presenting the effects of abamectin, ivermectin, eprinomectin, doramectin, emamectin, moxidectin, and spinosad on individual species of terrestrial and aquatic invertebrates as well as plants and algae. Since dung dwelling organisms are particularly important non-targets, as they are exposed via dung from treated animals over their whole life-cycle, the information on the effects of MLs on dung communities is compiled in an additional table. The results of this review clearly demonstrate that regarding environmental impacts many macrocyclic lactones are substances of high concern particularly with larval instars of invertebrates. Recent studies have also shown that susceptibility varies with life cycle stage and impacts can be mitigated by using MLs when these stages are not present. However information on the environmental impact of the MLs is scattered across a wide range of specialised scientific journals with research focusing mainly on ivermectin and to a lesser extent on abamectin doramectin and moxidectin. By comparison, information on compounds such as eprinomectin, emamectin and selamectin is still relatively scarce.

## 1 INTRODUCTION

Fermentation of soil-derived microbes under defined conditions and the screening of the resultant fermentation broths or their partially purified extracts is a long-honoured methodology for finding new chemical structures showing desirable biological activities [1]. During the 1980s, the field of veterinary medicine was revolutionized by the introduction of compounds showing strong activity against both ectoparasites and endoparasites which were thus termed as endectocides [2-3]. The earliest known such compounds were the avermectins with their potent anthelmintic and other insecticidal activities. Subsequent studies have resulted in the development of the milbemycins which have similar properties to the avermectins. More recently, fermentation processes have been used to isolate a whole new group of related chemicals, the spinosyns, which possess similar biological activity but have a different mode of action [4-5].

The avermectins, milbemycins and spinosyns are collectively referred to as macrocyclic lactones (MLs) which comprise several classes of chemicals derived from cultures of soil micro-organisms. Such compounds are extensively and increasingly used in veterinary medicine and agriculture. At least the newer ones are designed to have a specific mode of action in order to minimize side effects on beneficial species. In addition, many of them are persistent in the environment (*e.g.* soil, livestock faeces). Extensive data already exist about ecotoxicological effects of MLs on aquatic and terrestrial organisms and wildlife, and several comprehensive reviews on ecotoxicological and environmental effects are available [6-13].

### 1.1 Avermectins

Avermectins and the structurally related milbemycins are macrocyclic fermentation products of *Streptomyces avermilitis *and *Streptomyces cyanogriseus *respectively [14-15]. Eight naturally occurring novel macrocyclic lactones, namely avermectin A_1a_/A_1b_, A_2a_/A_2b_, B_1a_/B_1b_, B_2a_/B_2b_, have been discovered. Compounds of the B series of avermectins were found to be extremely active against helminths and arthropods. Subsequent chemical modifications resulted in the synthesis of ivermectin (22,23-dihydroavermectin B_1_), containing at least 80% 22,23-dihydroavermectin B_1a_ and no more than 20% 22,23-dihydroavermectin B_1b_ [16]. The avermectin structures are closely related complex 16-membered macrocyclic lactones. They share structural features with the antibacterial macrolides and the antifungal macrocyclic polyenes, but usually they are not grouped with these compounds, as they have neither antibacterial nor antifungal activities and do not inhibit protein or chitin synthesis as do the other two groups [17]. 

Ivermectin and avermectin B_1_ (abamectin) are generally used to control the ecto- and endoparasites (mites and nematodes) of livestock and antifilarial chemotherapy in humans [18]. Other forms of avermectins are also available for veterinary treatments in fish farms [19-20], and also for heart-worm chemotherapy in companion animals (*e.g.* elamectin). The benzoate salt of emamectin (derived from abamectin) in particular has found wide-spread use as an insecticide and also is commonly used in fish farms to eradicate fish lice (Copepod). Abamectin is also used as a pesticide to control mites and other crop pests [21]. 

Ivermectin is the most widely used avermectin and, as a result, large amounts of (eco)-toxicological information has been accumulated, particularly with respect to its use in cattle [22]. Since the first avermectins were commercialized, many novel avermectin derivatives have been developed mainly in crop protection [23, 24]. 

### 1.2 Milbemycins

Moxidectin (MOX), the most important milbemycin, is a semisynthetic methoxime derivative of nemadectin, a fermentation product of *Streptomyces cyanogriseus* subsp. *noncyanogenus*. Chemically, avermectins differ from each other by chain substitutions on the lactone ring, whilst milbemycins, which are structurally related, differ from the avermectins through the absence of a sugar moiety from the lactone skeleton [14, 25-26]. Milbemycin oxime is used against intestinal nematodes in dogs and cats, against adult heart-worm in dogs, and against ectoparasites in companion animals. Milbemectin (a mixture of ≥ 70% milbemycin A_4_ and ≤ 30% milbemycin A_3_) is an insecticide and acaricide effective against all development stages of mites. It is also active against pinewood nematode [27].

### 1.3 Spinosyns

The spinosyns are members of a new class of MLs with a unique mechanism of action involving disruption of nicotinic acetylcholine receptors. Their core structure is a polyketide-derived tetracyclic macrolide appended with two saccharides (an amino sugar (D-forosamine) and a neutral sugar (tri-O-methyl-L-rhamnose)), with a unique cross-bridged macrocyclic structure [28]. 

Aerobic fermentation of the actinomycete *Saccharopolyspora spinosa* [29], a soil-inhabiting micro-organism found in soil samples, produces mixtures of several analogs with two dominating forms, known as spinosyn A and D. Spinosad is a defined combination of the two principal fermentation factors, spinosyns A and D (thus its name, spinosAD). Structure-activity relationships have been extensively studied to increase activity and, importantly, minimize non-target impacts, leading to development of a semisynthetic second-generation derivative, spinetoram [30-31]. 

Spinosyns (mostly spinosad) are used to control crop and stored grains pests, and also for fly and mosquito control. Spinosad (SPI) is a neurotoxin which acts as a contact and stomach poison [4-5, 32] and has been shown to be an effective pest control agent [33-35]. Potential applications of SPI also have been investigated in the field of animal health [36]. Spinetoram offers increased efficacy over a larger range of susceptible pest insects with a similar environmental and toxicological profile to its parent compound, SPI. The residual activity of spinetoram was shown to be about 4-fold higher than SPI against codling moth larvae, and more than 6-fold higher against tobacco budworm larvae [31]. 

The main objective of this paper is to compile and critically review the present knowledge about the acute and chronic ecotoxicological effects on organisms, mainly invertebrates, of MLs in the terrestrial and aquatic environment. Detailed information is presented on the mode-of-action and the ecotoxicity of each of the most important compounds representing the three groups of MLs (avermectins, milbemycins and spinosyns). The legal requirements related to the marketing authorization (when used as veterinary pharmaceuticals) or registration (when used as pesticides) of these compounds is also briefly summarized, since most of the data provided in this review were gained when studying the effects of MLs on non-target organisms, *i.e.* as part of an environmental risk assessment. 

## 2 LEGAL TESTING REQUIREMENTS FOR MACROCYCLIC LACTONES

MLs are now widely used around the world with registrations in over 60 countries, including Canada, many European countries, India, Argentina, Japan, Australia, New Zealand, Zimbabwe and United States of America. The environmental assessment of avermectins by the US Food and Drug Administration was reviewed by Bloom and Matheson [37], while in Australia, the National Registration Authority for Agricultural and Veterinary Chemicals (NRA), now known as the Australian Pesticides and Veterinary Medicines Authority, has reviewed the registrations of the macrocyclic lactones: ivermectin (IVM), abamectin (ABM), moxidectin (MOX), doramectin (DOR) and milbemycin in terms of the effects of these products on dung insects and dung degradation [10]. Particular emphasis was placed on coprophagous beetles originally introduced into Australia under the CSIRO Dung Beetle Program to improve dispersal of cattle dung and control dung flies. The fate and effects of pesticides in the environment have been studied intensively for many years [38-40], and has been regulated for about 20 years in the European Union (EU) (*e.g.* EC 1991; EPPO 2003; EFSA 2007) [41-43]. This paper will focus on the environmental testing requirements for Veterinary Medicinal Products (VMPs) in the EU since relatively little attention has been given to these substances [44]. Over the past twenty years, the scientific community has become increasingly interested in the impacts of veterinary medicines in the environment, and there have been significant developments in the regulatory requirements for the environmental assessment of veterinary products [45]. Release of VMPs to the environment occurs directly from the use of medicines in fish farms, and indirectly via the application of animal manure containing excreted products to land or via direct excretion of residues onto pastures. Regulatory agencies have issued detailed guidelines on how VMPs should be assessed for possible unwanted effects on the environment. As long as 20 years ago, the EU has issued Directive 81/852/EEC [46] which requires pharmaceutical companies submitting a new product for registration to provide information that would assist in the assessment of the risk that such compounds may pose for the environment. Risk is the estimation of the relationship between the level of exposure to a substance, and the incidence and severity of an effect [47]. In ecological or environmental risk assessment (ERA) many species and processes may be exposed to chemicals by a variety of routes [48]. 

In the EU, the evaluation of the environmental risk of VMPs within marketing authorisation procedures has been discussed since the mid-nineties [48], and a first guidance document on how to perform an ERA was prepared by the European Medicines Agency in 1997 (EMA 1997) [49]. Later on, the International Cooperation on Harmonisation of Technical Requirements for Registration of Veterinary Medicinal Products (VICH; http://www.vichsec.org/) established rules for the ERA of VMPs that follow a two-phase approach [50-51]. In Phase I, exposure scenarios, *i.e.* intensively reared and/or pasture animals, are selected based on the application and the properties of the VMPs and predicted environmental concentrations (PEC) are estimated based on the dose and frequency of the application [50]. If PEC for soil exceeds the trigger value of 0.1 mg kg^-1^ dry weight (d.w.), studies on the environmental fate and effects on selected non-target species such as soil and dung organisms have to be performed in Phase II, Tier A [51]. A Phase II assessment is mandatory for endo- and ectoparasiticides regardless of the outcome of the Phase I assessment. Higher-tier studies (*e.g.* field studies) must be performed if a risk is identified in Phase II. In order to ensure the quality of the data and to allow comparability of the results, tests for the ERA should be performed according to standardised international guidelines whenever these are available, *e.g.* OECD (Organisation for Economic Co-Operation and Development) or ISO (International Organisation for Standardisation). Although laboratory test methods for assessing effects of veterinary pharmaceuticals on dung organisms became available recently [52-53], no methods have been standardised so far for higher tier tests, in particular field studies [54]. For the EU, additional guidance in support of the VICH guidelines is provided by EMA (2008) [55].

Since the book edited in 1989 by Campbell [2], several new MLs (*e.g.* doramectin (DOR), eprinomectin (EPR), milbemycin-oxime, moxidectin (MOX), selamectin) have been developed for the control of internal and external parasites, and other MLs are being used in agriculture and aquaculture. 

Despite their adverse effects on invertebrates, it was concluded in registration dossiers of companies that MLs would not have significant non-target effects due to physio-chemical properties that control their environmental fate and exposure potential. These assumptions were based on laboratory and modelled predictions, but were very difficult to verify in replicated mesocosm studies [56] despite the fact that such studies are considered to be a useful tool for the risk assessment of veterinary medicines [57]. 

As a case study, Liebig *et al.* [58-59] performed an environmental risk assessment of IVM mainly according to international and European guidelines (VICH 2000, 2004; EMA 2008) [50-51, 55] using a large number of new data on fate and effects of IVM and additional results from two-species tests, multi-species tests, semi-field and field studies. Previous ERAs for IVM had revealed no concern for the aquatic compartment. Effects on dung-insect populations had been considered as transient and thus not relevant. In contrast to these ERAs, the new case study – although in part preliminary – clearly demonstrates unacceptable risks (*e.g.* for daphnids and dung organisms) and, hence, suggests the necessity of reassessing ivermectin-based veterinary medicinal products. Furthermore, the case study indicates several gaps of the existing guidelines, which should be considered within guideline revision processes. Based on the outcome of the ERA, risk mitigation measures may be necessary to avoid the possible entry of IVM into the environment. The requirement and definition of risk mitigation measures within the registration and authorisation procedures for veterinary pharmaceuticals is a common practice [60]. Unfortunately, comparable comprehensive ERAs or reviews according to current requirements have not been performed for other MLs.

For the purposes of this review, the ecotoxicity of MLs will be considered successively in their action on organisms and environment, regardless of their use. Particular attention will be given to coprophagous organisms since ML compounds are excreted by animals mainly in the faeces. The role of dung beetles and earthworms in the complex process of degradation of animal faeces is considered an important ecosystem service which ensures the stability and sustainability of grazed ecosystems [61].

## 3 CHEMISTRY AND MODE OF ACTION OF MACROCYCLIC LACTONES

### 3.1 Structure and Chemical Properties

The most important structural difference between avermectins and milbemycins is a bisoleandrosyloxy substituent found at the 13-position of the macrolide ring of the avermectins, whereas that position is unsubstituted in milbemycins. Also, there can be several different alkyl substituents at C-25 in both groups. Removal of the 13-hydroxy group from avermectin aglycones gives 13-deoxyavermectin aglycones which are closely related to certain milbemycins [17]. Essentially, the molecular structures of the two groups are superimposable and one can think of the avermectins as glycosylated milbemycins, or of the milbemycins as deglycosylated avermectins [14].

The lipophilic bisoleandrosyl moiety at the C-13 position of the avermectins is clearly not obligatory for biological activity, but it has provided a convenient target for chemical modification [14, 17]. The 4"-position has been the most frequently studied because of its easy access. Acyl [62], amino [63], or thio [64] substitutions at this site have changed solubility, distribution, stability, and diversity of spectrum, while maintaining the overall potency of the parent molecule. Many synthetic modifications at the terminal sugar of avermectins offer derivatives having potent and improved bioactivity [65]. Avermectin aglycones, monosaccharides, and the naturally occurring disaccharides have been further modified by attaching various sugars to the different hydroxyl groups, the derivative of avermectin demonstrating various anthelmintic efficacity [17, 66]. Two potential metabolites of ivermectin were identified in cattle dung after animal treatment: 24-hydroxymethyl-H_2_B_1a_ and 300-O-desmethyl-H_2_B_1a_ [67]. These metabolites were also reported to be the most prominent in cattle and swine liver [68-69]. The amount of the metabolites was estimated to be less than the amount of parent compound [67]. In addition, the more polar degradation products of ivermectin (monosaccharide and aglycone), as detected as transformation products in soil, were shown to be less toxic to daphnids than the parent compound [70].

The physical/chemical properties of a compound determine its fate in the environment [22]. Avermectins are unlikely to volatilize and be distributed into the atmosphere, due to their high vapour pressure (Table **1**). Their solubility in water is relatively low; the time at which 50% of ivermectin has disappeared (DT_50_) from the water phase was found to be less than 6 h mainly due to the rapid sorption to the sediment [71]. A DT_90_-value in water of 16.8 d was determined while for the entire aerobic sediment/water system, a DT_50_-value of 127 d was determined. It reflects that transformation of ivermectin into TPs (transformation products) and bound residues was relatively slow [71]. Avermectins are soluble in methanol, chloroform, p-dioxane, dimethylformamide, ethyl acetate, 95% ethanol, diethyl ether, methylene chloride, acetone and aromatic hydrocarbons. Avermectins also have a high adsorption coefficient (K_oc_), indicating that they are not likely to accumulate in the water column. The accumulation of ivermectin in the environment is likely due to its hydrophobic property (log K_ow_ = 3.2) and the resulting high affinity to organic matter [72]. This was confirmed by tests that measured the degree of binding between ivermectin and a wide variety of soil types [70, 73]. Laboratory and field experiments have demonstrated that ivermectin residues bind tightly to soil [70, 74]. Compounds possessing K_oc_ > 1000 are considered tightly bound to organic matter in soil and immobile in the environment. Ivermectin has a K_oc_ of 12 600 and 15 700, depending on soil type, and is therefore classified as immobile (Table **1**). The octanol/water coefficient (K_ow_) of ivermectin, which is an indication of its affinity for lipids, is high enough to raise concerns about its bioconcentrating in fat tissues of species. The high K_ow_ of ivermectin is likely balanced by its large molecular weight, making it difficult to cross biological membranes (Table **1**). 

### 3.2 Mode of action of Avermectins

The gamma-aminobutyric acid (GABA) is a common neurotransmitter found in most invertebrates and in the central nervous systems of vertebrates [75-80]. Avermectins inhibit the GABA neurotransmission at two or more sites in nematodes [81], blocking interneuronal stimulation of excitatory motoneurons and thus leading to a flaccid paralysis [14-15, 82]. The drug is believed to block nerve signals by interfering with the glutamate-gated chlorid (GluCl) channel receptors (found only in invertebrates) [83], which make them likely to affect the membrane stability [84]. Exogenous glutamate inhibits pharyngeal pumping, which is mimicked by IVM [85], while paralysis of somatic muscles is associated with GABA-gated chloride channel receptors [86]. The target species become paralysed and die as a result of inhibition of inter-neural and neuromuscular transmission [87-88]. In arthropods, the avermectins interfere with the transmission among nervous and muscular cells, because the GABA receptors are located at the neuromuscular junction. In vertebrates, where GABA receptors are located mostly in the brain, avermectins also interact with the GABA receptors but their affinity for the invertebrate receptors is approximately 100 times greater [89]. The lack of effect of IVM on the mammalian nervous system at therapeutic concentrations is probably because it is a large molecule. Thus, vertebrates (mostly mammals) are normally protected from the effects of avermectins by the blood-brain barrier [90]. However, although signs of toxicosis have not been observed in collie dogs treated repeatedly with IVM at doses ≤ 60 µg kg^-1^ of body weight, certain genetic lines of collies (approximately 35% of all collies treated with 120 µg IVM kg^-1^) develop mild to moderate signs of toxicosis [91-92]. Radio-labelled IVM has been detected also in the brain of Atlantic salmon (*Salmo salar*) administered with IVM at normal treatment doses [93]. In the mite *Tetranychus cinnabarinus*, the major resistant mechanism to ABM was the increasing activities of carboxylesterases (CarE) and glutathione-S-transferase (GST) and the increase of mixed function oxidase of O-demethylase activity, which was probably because the molecular structure of ABM had the oxymethyl group (–OCH3) [94].

ABM has been considered to be a potent inhibitor of reproduction in some insects. When invasive red fire ant queens (*Solenopsis invicta*) were exposed to low doses, several histological impacts on the reproductive systems were noted, including hypertrophy of the epithelial cells surrounding eggs, reduced egg production and size, abnormal clumping of chromatin in the nurse cells (pycnosis), and the absence of egg yolk within the eggs [95]. These results suggest direct action on the endocrine system rather than simply an indirect effect of reduced feeding activity. Emamectin benzoate can interfere with the function of the moult-inhibiting hormone and disrupt endocrine systems in American lobster (*Homarus americanus*). Lobsters force-fed slurry containing emamectin benzoate moulted sooner than non-exposed lobsters. Furthermore, exposed lobsters that were bearing eggs aborted their broods [96]. 

Developmental abnormalities, also known as fluctuating asymmetry, have been observed in flies exposed to IVM-treated faeces. Adults of *Musca vetustissima *(Diptera) emerging from outdoor cow pats treated with ABM showed higher levels of fluctuating wing asymmetry [97]; significant differences in the symmetry of wing venation patterns were observed also in *Scatophaga stercoraria *(Diptera) exposed to dung containing 0.0005 mg kg^-1^ IVM [98]. Increases in fluctuating asymmetry have been linked to developmental instability as a consequence of genomic and/or environmental stress [99]. It should be noted however that several studies have been unable to detect fluctuating wing asymmetry [100-101].

### 3.3 Mode of Action of Milbemycins

Although the antiparasitic activity of the milbemycins has been described for more than two decades, their mode of action is still not well understood, particularly when compared with the avermectins [14, 102]. Nemadectin is the dominant member of the class of milbemycins, bearing unsatured longer chain groups at the 25-position. Nemamectin shows pronounced nematocidal and insecticidal activity and it is the starting material for moxidectin (MOX), a commercial endectocide.

MOX works in two ways: in common with other macrocyclic lactones, it displays a high affinity for the glutamate-gated ion channels specific to invertebrates. These glutamate-gated binding sites apparently occur in close proximity to GABA-gated chloride channels, and the macrolide endectocides may increase GABA-gated sites as well. MOX as well as IVM bind to receptors on neuronal membranes of nematodes and myoneural junctions of arthropods. The chloride ion influx lowers cell membrane resistance and causes a hyperpolarization of the post-synaptic cells. This in turn makes neurotransmission more difficult and results in flaccid paralysis, death and/or expulsion of the parasite [85, 75-77, 103-104]. In rats, MOX may activate the GABAergic system, resulting in a reduced motor coordination arising from the inhibition of striatal dopamine release [105].

With the commercial success of ivermectin, several hundred analogs of avermectin and milbemycin were tested in narrow-spectrum *in vitro* and *in vivo* tests and for broad-spectrum nematode, and to a lesser extent, arthropod activity in sheep, cattle, and dogs [14]. Each compound has its own unique 'spectral fingerprint', with its own strengths and its own dosage-limiting species. Although each avermectin and milbemycin maintained the same relative potency *in vivo* as in the narrow spectrum *in vitro* test, all required at least 0.2 mg kg^-1 ^to eliminate the dosage-limiting species for the full broadspectrum, and a dose of 0.5 mg kg^-1^ b.w. is needed for pour-on doses [14]. The increased dosage of the pour-on formulation (0.5 mg kg^-1^) increases the ecotoxic potential of this formulation [106]. Sommer and Steffansen [107] reported higher ivermectin concentrations (9.0 mg kg^-1^) in dung of cattle given the pour-on product compared with dung (3.9 mg kg^-1^) of cattle given the injectable formulation, but they had comparable persistence. 

### 3.4 Mode of Action of Spinosyns

The mechanism(s) by which spinosyns derive their insecticidal activity are thought to differ from those of other avermectins, though there is still much to be learned about the precise nature of their mode of action [108-109]. Several studies suggest that the spinosyns disrupt neural functions, most likely via an alteration of nicotinic receptor function [101-113, 317]. Spinosyn A, the principal constituent of the insecticide spinosad, does not interact directly with known binding sites of insect nicotinic receptors, including nicotinic or γ-aminobutyric acid (GABA)-based insecticidal target sites. Nor does spinosyn A interact with the target site for avermectins such as ABM [114]. The absence of interaction with well-known insecticide target sites supports the hypothesis developed by Orr *et al.* [114] that spinosyn A exerts its insecticidal actions via a novel mode of action. The activation of nicotinic currents by spinosyn A as described [110-111, 115] would suggest that spinosyn A is interacting with an as yet unidentified nicotinic receptor subtype. Recent knockout studies in *Drosophila melanogaster* implicated the Dα6 subunit of the nicotinic acetylcholine receptor as a target site of spinosyn [116-117].

## 4 ECOTOXICITY OF AVERMECTINS

### 4.1 Ecotoxicity of Abamectin

A summary of ecotoxicology data for ABM is provided in Table **2**. 

#### 4.1.1 Micro-Organisms

The avermectins have neither antibacterial nor antifungal properties [162]. The luminescent bacteria *Vibrio fischeri* exposed to ABM for 30 minutes presented an EC_50_ of 0.7 mg L^-1^ [118]. 

#### 4.1.2 Plants

The inhibition of specific growth rates for each concentration of ABM was calculated for the green unicellular algae *Scenedesmus subspicatus*, with an estimation of the 72-h EC_50_ [118]. There was no growth inhibition at 10 µg L^-1^ of ABM and the 72-h EC_50_ was found to be 4.4 mg L^-1^ of ABM. Ma *et al.* [119] reported 96-h EC_50_ values of 9.9 mg L^-1^ and 7.3 mg L^-1^ for *Scenedesmus obliquus* and *C. pyrenoidosa*, respectively. The concentration of ABM calculated to decrease frond production in *Lemna gibba* (duckweed) by 50% was 3.9 mg kg^-1^ [120]. 

#### 4.1.3 Terrestrial Organisms

##### 4.1.3.1 Mites

ABM rapidly degrades on plant surfaces [121]; therefore, residual activity depends on pests feeding on foliage that has absorbed the toxicant [122]. ABM is essentially nonphytotoxic (but available data mostly concern DOR; see Table **5** below), permitting its widespread use in crop protection. The impact of ABM on many species of insect and mite, particularly those regarded as pests, has been documented by Dybas & Green [122] and reviewed by Dybas [123]. Under laboratory conditions ABM is a highly toxic contact poison to the eriophyid mite *Phyllocoptrutta oleivora* (citrus rust mite) on leaf disc, with an LC_90_ of 0.02 mg kg^-1^ [124]. The contact effect against adult mites *Polyphagotarsonemus latus* (broad mite) gives quite similar results (LC_90_ = 0.05 mg kg^-1^) [122], whereas *Paronychus citri *(citrus red mite) reveals more resistance to ABM (LC_90_ = 0.24 mg kg^-1^) [122].

Several studies have shown that ABM is highly toxic to tetranychid spider mites (plant-feeding mites) under laboratory conditions, with LC_90_ values against adult mites in the range of 0.02 to 0.06 mg kg^-1^ [122-123, 125]. The comparison of a field population of *Tetranychus cinnabarinus* (carmine spider mite) with a laboratory colony of *T. urticae* (twospotted spider mite) showed that both species were highly susceptible to ABM, with an LC_50_ value of 0.0029 mg kg^-1^ for *T. cinnabarinus* compared to an LC_50_ of 0.0087 mg kg^-1^ for the laboratory colony of *T. urticae* [123].

Beneficial organisms such as predatory mites are also highly susceptible to ABM. The use of ABM in an integrated pest management (IPM) system should be carefully evaluated in field tests. In bioassays conducted with fresh residue of ABM sprayed on leaves and left to dry (0.01 ng cm^2^ a.i.), mortality of *Phytoseiulus plumifer* (Acari: Phytoseiidae) protonymphs was 100% [126]. These results are consistent with previous observations on this species [127], and similar results were obtained with ABM on *Phytoseiulus persimilis* [128] and *Neoseiulus cucumeris* [129]. The residual toxicities of ABM on leaflets to the phytoseiid mites *Galendromus occidentalis* and *Phytoseiulus persimilis* were assessed up to 37 days post-treatment at a concentration of 93.0 mg kg^-1^ ABM. Impacts on mortality, fecundity and fertility were determined following 3 days of exposure to each leaf surface residue interval. ABM significantly increased mortality of adult females of *G. occidentalis* 3 days after treatment vs 6 days for *P. persimilis.* Fecundity of *G. occidentalis* decreased significantly on only the first observation date (3 days) following treatment, contrary to *P. persimilis* (reduction for 14 days) [130]. The effects of ABM were short-lived with *G. occidentalis* but slightly persistent in the case of *P. persimilis*. Several other authors reported that exposure to ABM residues did not have a significant effect on *P. persimilis* mortality [131-133]. 

After 42 generations (laboratory selection), *Tetranychus cinnabarinus *became resistant to ABM: the LC_50_ values (contact with ABM) ranged from 0.02 mg L^-1^ (generation F0) to 0.15 mg L^-1^ (generation F42) [94] (Table **2**). Resistance was partially suppressed by piperonyl butoxide (PBO), diethyl maleate (DEM) and triphenyl phosphate (TPP), inhibitors of mixed function oxidase (MFO), glutathione S-transferases (GST), and hydrolases, respectively, suggesting that these three enzyme families are important in conferring ABM resistance in *T. cinnabarinus* [94]. Such values are of the same order of magnitude as those of other mites which are considered to be the most important pests of pastures and grain crops in Australia. LD_50_’s ranged from 30.2 mg ABM L^-1^ for *Penthaleus falcatus* (blue oat mite) to 154.6 mg L^-1^ for *Bryobia sp. *(clover mite). *Halotydeus destructor *(redlegged earth mite) showed intermediate values (97.7 mg L^-1^) (Table **2**) [134].

##### 4.1.3.2 Insects

ABM is also likely to affect other beneficial organisms. *Anagrus nilaparvatae* (Hymenoptera: Mymaridae) is a major parasitoid of the rice planthopper *Nilaparvata lugens* (Hemiptera: Delphacidae). When exposed for 1h to ABM (emulsible concentrate 1% ABM; final test concentration 16 mg ABM L^-1^), the LC_50_ for *A. nilaparvata* was 8.5 mg ABM L^-1^ (Table **2**) [135], with low contact and residual toxicity, but high oral toxicity. Short residual toxicity was also observed in adult parasitoids of *Dacnusa sibirica* (Braconidae) [132] and also in the pteromalid parasitoids of house flies *Nasonia vitripennis* and* Spalangia cameroni* when exposed for 1.5h to plywood boards treated with 0.001-0.1% ABM [136]. The direct contact or ingestion of ABM also had a significant negative effect on *Diglyphus isaea* (Hymenoptera: Eulophidae), a widespread ectoparasitoid of leafminer larvae. The latter species has been reported from a large number of host species, but commercially is of interest as a parasite of *Liriomyza bryoniae*,* L. trifolii*,* L. huidobrensis *and the chrysanthemum leafminer *Phytomyza syngenesiae* (Diptera, Agromyzidae) [137]. This adverse effect of ABM on parasitoids might be associated primarily with oral toxicity. ABM is highly toxic to adult *L. trifolii* and early instar larvae mining within leaf tissue. When applied to newly eclosed larvae on chrysanthemum, ABM provided 100% control at 12 mg kg^-1^ concentration. However 48 mg kg^-1^ was required to provide 100% kill of third stage larvae and complete inhibition of pupation and adult emergence [123, 138].

The susceptibility to ABM of the multicoloured Asian ladybird beetle *Harmonia axyridis *(also considered to be a beneficial arthropod) has been examined for all developmental stages [139]. This species is a generalist predator that feeds primarily on several aphid species and has been recognized for its potential contribution to the integrated management of various crop aphids. In laboratory tests, all instars and the adult stage were treated topically with 1 µL of ABM on the ventral abdomen with a micro-applicator at the concentration of 9 mg ABM L^-1^. ABM was highly toxic to eggs, larvae, pupae, and adult ladybirds at rates less than the recommended doses (Table **2**). When first and second instars were exposed to ABM, the survival rate was zero. The LC_50_ was <0.09 mg ABM L^-1^ for eggs and larvae until 3^rd^ instar, LC_50_ was 18.4 and 4.9 mg ABM L^-1^ respectively for the 4^th^ instar and adults. 

The toxicity of ABM to bees has been assessed in laboratory studies by applying it directly to the bees, by putting it in their food, and by exposing the bees to foliage that had been treated at various times prior to harvesting [120]. ABM was found to be quite toxic as a contact poison to the bees, with LD_50_ values of 2 and 17 ng bee^-1^ at 24 and 48 h, respectively. When ABM was fed to the bees, the LD_50_ was 9 ng bee^-1^. However foliage that had been treated with ABM 24 to 48 h earlier was not toxic [120].

Other beneficial organisms can be affected by the use of ABM for crop protection. The mortality of 2nd instar larvae of the common green lacewing *Chrysoperla carnea* (Neuroptera: Chrysopidae) was assessed at 24, 48 and 72 h after spraying leaves with a solution of ABM (0.25 mL of formulated compound (19 g a.i. L^-1^) per litre water). At 48 h after treatment, ABM showed no acute toxicity to chrysopid larvae (contact with leaves) [140], and this low toxicity of ABM to lacewings has been previously reported in other studies [141-142]. In contrast, ABM reduced the numbers of the strawberry pests *Anthonomus rubi* (weevil)*, Lygus rugulipennis* (plant bug) and *Chaetosiphon fragaefolii* (strawberry aphid), and the predatory mite *Phytoseiulus persimilis*. The difference in mortality between treatment and control for *P. persimilis* after 48 h was significant, and in accord with what has been reported in other experiments [143].

LC_90_ values of 0.02, 1.5 and 6.0 mg kg^-1^ from foliage bioassays performed in the laboratory were reported for ABM against first instar larvae of, respectively, the tobacco hornworm *Manduca sexta* (Sphingidae), the corn earworm *Heliothis zea* (Noctuidae) and the southern armyworm *Spodoptera eridania* (Noctuidae). These values indicate a 300-fold difference in sensitivity of southern armyworm compared to tobacco hornworm [122]. In Pakistan, *Spodoptera litura* developed a possible cross-resistance between ABM and other insecticides, with an LC_50_ ranging between 18.5 (susceptible laboratory population) and 2342 mg.L^-1^ (field strain) (Table **2**) [144]. In the 15 field populations tested, two populations showed very high levels of resistance to ABM, indicating sensitivities 196- and 127-fold higher than that recorded in a susceptible laboratory population (Lab-PK). Seven populations showed high levels of resistance when compared to the Lab-PK strain, with resistance ratios ranging from 31.8 to 79.7.

##### 4.1.3.3 Collembolans

The susceptibility of many organisms of the soil fauna to ABM has been investigated (Table **2**). For collembolans, the LC_50_ values ranged between > 2.5 and 67.0 mg ABM kg^-1^ dry soil for *Folsomia candida* [121-122] and 0.8 mg ABM kg^-1^ dry soil for *F. fimetaria* [145]. After 28 days of exposure, the EC_50_ reproduction values were of similar amplitude, ranging from 13 mg kg^-1^ dry soil in *F. candida* to 0.3 mg kg^-1^ in *F. fimetaria*. The EC_50_ values for mortality and reproduction of *F. candida* obtained with ABM contained in sheep faeces were lower from those obtained with soil substrate (respectively 1.0 and 1.4 mg kg^-1^ dry faeces), but dung and soil characteristics are very different [124]. In the collembolan *Sminthurus viridis*, the LD50 value of 18.9 mg L^-1^ was obtained after 8h of exposure (contact) to ABM [134].

##### 4.1.3.4 Isopods

The toxicity of ABM to *Porcellio scaber* upon 21 days of exposure in spiked Lufa 2.2 soil was low, with an LC_50_ of 69 mg ABM kg^-1^ (range of 48 to 89 mg kg^-1^) [146].

##### 4.1.3.5 Nematodes

There are a number of important plant parasitic nematodes limiting crop production in temperate, tropical and sub-tropical agriculture [147-148]. For example, *Pratylenchus zeae*, a migratory endoparasitic nematode, is often encountered in maize cultivars throughout the world and causes significant yield losses [149]. *Heterodera schachtii *is a sedentary endoparasitic cyst nematode that causes significant levels of damage to sugar beet in most major growing areas [150]. *Meloidogyne incognita *is a sedentary endoparasitic root-knot nematode that reduces yield in many crops worldwide, for example in large and small scale cotton production [151]. Cabrera *et al.* [152] investigated the efficacy of ABM when applied as a seed treatment on maize, cotton and sugar beet to enhance nematode management by giving high levels of nematode control at low cost. The EC_50_ and EC_80_ of ABM seed treatment in maize against *P. zeae *was established at 0.16 and 1.0 mg ABM per seed, respectively. The EC_50_ and EC_80_ in cotton against *M. incognita *was 0.02 and 0.3 mg ABM per seed, respectively. Against *H. schachtii *in sugar beet, the EC_50_ was 0.03 mg ABM per seed and the EC80 was not attained at the doses tested. The penetration of *Pratylenchus zeae* was reduced more than 80% in maize at a dose of 1.0 mg ABM seed^-1^.

##### 4.1.3.6 Earthworms

The toxic effects of ABM on earthworms have been examined using *Eisenia fetida* (compost worm) exposed at concentrations ranging between 0 and 5 mg kg^-1^ soil (dry weight) [153]. ABM showed significant toxicity on the growth of earthworms with increasing concentrations up to 5 mg kg^-1^, the most sensitive parameter being reproduction (cocoon production and hatchability). The number of cocoons was reduced at concentrations above 0.25 mg kg^-1^ and no cocoons were present at the highest concentration of 5 mg kg^-1^. Cocoons exposed to ABM exhibited a reduced hatching success at concentrations above 1.5 mg kg^-1^ (Table **2**). In another study with *E. fetida*, LC_50_ survival was estimated at 28 mg kg^-1^ after 28 days exposure [120]. For another earthworm, *Eisenia andrei*, the LC_50_ value was 18 mg kg^-1^ dry soil [146], with a NOEC value for reproduction <0.25 mg kg^-1^ of wet soil [145]. The sensitivity of enchytraeid worms is less, with an EC_10_ survival = 78.3 mg kg^-1^ dry soil [145] and EC_50_ = 111 mg kg^-1^ dry soil [146]. The LC_50_ survival of *Enchytraeus crypticus* was estimated at 1.1 mg kg^-1^ after 28 days exposure to ABM contained in sheep faeces [146] (Table **2**).

##### 4.1.3.7 Diptera 

ABM is generally regarded as being the most toxic of the MLs registered for veterinary use [9, 13], particularly among the higher Diptera (Table **2**). Concentrations of ABM as low as 8 μg kg^−1^ dung inhibited the survival of larvae of *Haematobia irritans exigua *[66], whereas larvae of the closely related *H. irritans *(horn fly) failed to develop in dung voided by cattle treated 7-14 days previously [154]*. *Assays on the bush fly, *Musca vetustissima *[155-157], indicated that residue levels in dung dropped by cattle injected with ABM were sufficient to inhibit larval survival for at least 2-5 weeks post-treatment. In one assay [158], flies emerging from dung voided 4 weeks after ABM treatment showed significantly enhanced levels of fluctuating asymmetry when compared with flies emerging for control dung. 

##### 4.1.3.8 Dung Beetles

Survival of larvae of *Digitonthophagus* (= *Onthophagus*)* gazella* has been shown to be significantly reduced in ABM-spiked dung containing 8 µg ABM kg^-1^ of fresh faeces; concentrations of ≥ 16 µg ABM kg^-1^ resulted in 100% mortality [66]. Exposure of newly emerged adults of *Onthophagus binodis *to dung voided by cattle treated 3 and 6 days previously with ABM at a dose rate of 200 μg kg^-1 ^l.w. resulted in delayed ovarian development and reduced survival [159]. Dadour *et al.* [160] have reported that ABM residues excreted in dung up to 42 days after injection had a deleterious impact on ovarian condition, brood mass (egg) production, and larval survival [160]. In a similar study [157], breeding by *O. binodis* was inhibited for at least one week post-treatment and severely reduced for a further 3 weeks (Table **2**).

#### 4.1.4 Aquatic Organisms

##### 4.1.4.1 Fish

The response of fish species to ABM is much more uniform than that observed in invertebrates. The LC_50_ for rainbow trout is 3.2 µg kg^-1^, while that for bluegill sunfish is 9.6 µg kg^-1^. The sheepshead minnow (*Cyprinodon variegates*) had an LC_50_ of 15 µg kg^-1^. The channel catfish (*Ictalurus punctatus*) and the carp had higher LC_50_ values, of 24 and 42 µg kg^-1^, respectively [120] (Table **2**).

##### 4.1.4.2 Crustaceans

Aquatic invertebrates vary widely in their sensibility to ABM, due to the mode of action of MLs. Crustaceans are very sensitive to ABM. Table **2** gives some values of LC_50_ (µg kg^-1^) obtained after 96h of exposure [120]. *Mysidopsis bahia* (mysid shrimp) was the most sensitive, with a LC_50_ of 0.022 µg kg^-1^, while *Penaeus duorarum* (pink shrimp) had a 96h LC_50_ of 1.6 µg kg^-1^, 2 orders of magnitude higher than the mysid shrimp. *Callinectes sapidus *(blue crab) and *Crassostrea virginica *(eastern oyster) were very much less sensitive to ABM, with 96h LC_50_ values of 153 and 430 µg kg^-1^, respectively. 

ABM has been shown to be highly toxic to *Daphnia magna* (Cladocera), the 24-h LC_50_ value (with mortality being the toxicity endpoint) being only 0.34 µg kg^-1^ [120]. This value was similar to that reported by Tišler and Eržen [118] from an acute toxicity test in a semi-static exposure system, and was obtained despite the fact that their toxicity endpoint was based only on the mobility of daphnids (24h- and 48h- EC_50s_ of 330 and 250 ng L^-1^ respectively). In a chronic toxicity test (semi-static), an ABM concentration of 9 ng L^-1^ still caused mortality and inhibited the reproduction of *D. magna* [118]. The NOEC was detected at 5 ng L^-1^ of ABM and the LOEC occurred at 9 ng L^-1^ (nominal concentrations). The 21-days IC_25_ (inhibiting concentration 25%) was 7 ng L^-1^. Daphnids are filter feeders and a possible reason for their extreme susceptibility to ABM is the uptake of ABM via algae. The results obtained by Tišler and Eržen [118] demonstrate that the daphnids were approximately 10 times more sensitive than reported by Wislocki *et al.* [120] who recorded a NOEL of 30 ng L^-1^ of ABM. The sensitivity of *Mysidopsis bahia* to ABM (NOEL 4 ng L^-1^) is similar to that of daphnids [120].

### 4.2 Ecotoxicity of Ivermectin

Literature on the effects of IVM on organisms is very numerous and in a review of this kind it is not possible to be exhaustive. The overall purpose of this section is to document the range of non-target effects of IVM on terrestrial and aquatic organisms and to present this as a basis for a risk assessment of their environment. Emphasis is placed on dung feeding invertebrates and the effects that faecal residues may have on pasture ecology, as IVM is used worldwide to control internal and external parasites of livestock. A summary of ecotoxicology data for IVM is provided in Table **3**.

Ivermectin is a mixture of two chemically modified avermectins that contain at least 80% of 22,23-dihydroavermectin-B_1a_ and >20% 22,23-dihydroavermectin-B_1b_. It is a highly lipophilic substance that dissolves in most organic solvents, but is practically insoluble in water (0.0004% m/v). It has exceptional potency against endo- and ectoparasites at extremely low dosage rates, normally expressed as µg kg^-1^. González Canga *et al.* [161] reviewed comprehensively the IVM spectrum of activity in several domestic animals and the distribution and pharmacokinetic parameters obtained after administration to ruminants and to monogastric species. IVM undergoes little metabolism, and most of the dose is excreted unchanged in the faeces of treated animals. Soil treatments in agriculture can affect earthworms and other soil organisms as the degradation half-life of IVM, in soil or faeces-soil mixtures, may range from 91 to 217 days in the winter and 7 to 14 days in the summer [70, 162].

Since its introduction in 1981, IVM has been the subject of numerous ecotoxicological studies. The first exhaustive review of IVM characteristics was published by Campbell in 1989 [2]. Some chapters refer to the use of MLs in agriculture and crop protection (mainly ABM) [120], whereas others deal with its use in veterinary medicine and its unintended effects on non-target organisms in pastures (dung beetles, flies, earthworms) [162-163]. The effects of IVM on dung-feeding arthropods have been reviewed comprehensively by Strong [164] and by Steel [10], the latter including much unpublished information on MLs submitted by pharmaceutical companies. This topic has been extensively developed in subsequent reviews [6-7, 9, 165], with a special 

attention to risks associated with the use of IVM in fish farms [8]. Floate *et al.* [13] and Kolar and Eržen [166] reviewed the nature and extent of the effects of parasiticides in dung, examined the potential risks associated with different classes of chemicals, and described how greater awareness of these non-target effects has resulted in regulatory changes in the registration of veterinary products. A more general review concerned the use of anthelmintics and the risks to non-target fauna in pastures [12].

#### 4.2.1 Dung Feeding Invertebrates

##### 4.2.1.1 Diptera

Following treatment, MLs are eliminated in the livestock faeces where they have a wide range of harmful affects upon certain characteristic insects that breed in dung. Few of these are pests, and many of which are beneficial. However the toxicity of MLs residues, in particular IVM residues, to the development of eggs and larvae of dung breeding flies has been extensively examined with a particular emphasis on pest species. Higher Diptera are particularly sensitive to drug residues and show a wide range of responses from death of larvae to delayed reproductive development, reduced fecundity, disruption of water balance, interference with moulting and emergence, and developmental abnormalities in surviving adults [167-171] (Table **3**). Differential sensitivity to excreted residues is especially evident among muscid flies. Mortalities of 47% and 87% were observed for *Neomyia cornicina* exposed for 7 days to dung containing 0.125 and 0.50 mg IVM kg^-1^ respectively [167]. Similar results were obtained by Wardhaugh and Rodriguez-Menendez [172] and by Lumaret *et al.* [173]. For *Scathophaga stercoraria*, the 24-h EC_50_ and 48-h EC_50_ were 0.05 and 0.04 mg IVM kg^-1 ^dung, respectively [98]. Wing abnormalities were observed with concentrations as low as 0.5 μg kg^-1^ [98], although in other cases such abnormalities were not observed in *M. vetustissima *[100] nor in *S. stercoraria* [101] (Table **3**). 

Dung voided by cattle treated subcutaneously with IVM inhibited the survival of *Musca nevilli* larvae for 49 to 56 days post-treatment [168]. Similar assays with *M. vetustissima* [174], *M. autumnalis* [175-176], and *M. domestica* [174] detected lethal effects for 28 to 35, 14 to 28, and 7 to 14 days, respectively. Treatment of cattle with an injectable formulation of 200 µg IVM kg^-1^ body weight inhibited larval growth and prevented emergence of adults of *Musca domestica* (house fly) [174, 177-178] and *M. vetustissima* (bush fly) [155-156] for 7-14 days post-treatment. An oral drench of 200 µg IVM kg^-1^ live weight to sheep prevented emergence of *M. vetustissima* for the first 4-6 days post-treatment of animals, with 100% survival at day 28 [100], and delayed reproductive development of *Lucila cuprina* (sheep blowfly) [169-170]. No larvae of *M. vetustissima* survived in faeces voided up to 39 days post-application of a controlled-release formulation of IVM to sheep [179]. In Malaysia, treatment of cattle with a sustained-release device of IVM (SR bolus formulation 1.72 g of IVM) prevented the establishment of myiases caused by *Chrysomya bezzania* (Old World screw-worm fly) for at least 102 days post-treatment [180]. In the same trials, faecal residues of IVM adversely affected the survival of two species of dung breeding fly (*Musca inferior* and *Orthelia timorensis*). Likewise, the dung of sheep treated with a controlled release capsule of IVM [179] prevented larval survival of the dung breeding fly *Musca vetustissima,* over an observation period of 39 days. Non-pest flies (or their larvae) including species of muscoids, sepsids, sphaerocerids and most Cyclorrhapha are also deleteriously affected [172, 177, 181].

Many of these studies have documented the biological consequences of treating livestock with MLs, but few have provided the information needed for preparing a proper risk assessment (*e.g.* EC_50_ and NOEC values). Guidelines developed by VICH 2004 [51] may require an environmental risk assessment when faecally excreted residues of veterinary pharmaceuticals are deemed to adversely affect non-target organisms that are responsible for the breakdown and re-cycling of animal faeces and the sustainability of the pasture ecosystem. To standardise tests required for the registration of veterinary pharmaceuticals, a standardised bioassay using *Musca autumnalis* has been developed to test the lethal and sublethal toxicity of parasiticide residues in livestock dung [182]. The repeatability of this test was assessed for the parasiticide IVM in seven trials performed in six laboratories in Germany, France and UK. The calculated effect concentration at which 50% emergence was observed (EC_50_) averaged 4.65 ± 2.2 µg IVM kg^-1^ fresh dung (range: 1.20 - 7.7). Effects on emergence were, with one exception, not observed below the NOEC ranging between 1.1 and 3.3 µg IVM kg^-1^. No effect on development time was observed. Authors concluded that *M. autumnalis* is suitably sensitive, and the methods sufficiently repeatable, to support use of this standardised bioassay by the international community in the registration of new veterinary pharmaceuticals. Following these considerations, this species was accepted as a possible test organism in a recently published OECD Guideline No. 228 [52].

##### 4.2.1.2 Dung Beetles

Larvae of dung beetles generally appear to be more sensitive to MLs residues than adults. Coleopteran larvae have biting mouthparts and feed on whole dung, whereas most adult beetles have specialised mouthparts that screen out the larger fragments of organic material [13, 183]. Because IVM attaches strongly to the particulate phase of digesta [162], filter-feeding adults are likely to imbibe less IVM than their bulk-feeding larvae. Moreover, larvae feeding within the brood ball repeatedly consume their own faeces during their period of development and hence increase their exposure to chemical residues [184].

Table **3** summarises the range of sensitivity of several species of dung beetles, with a comprehensive survey being already published by Steel in 1998 [10]. Preparations developed for topical administration or via injection have prolonged effects, generally affecting development and/or survival for at least 2 to 4 weeks. Bioassay data on SR devices used in sheep and cattle indicate that the blood-plasma levels needed to achieve reliable parasite control also result in the production of faeces that are likely to be toxic to coprophilous insects for the entire period that the devices are active (100 days for sheep and 135 days for cattle). Such results were obtained both in temperate (Europe) and tropical conditions (Malaysia) [180, 185]. In Europe, residues of SR bolus formulation with 12 mg day^–1^ over 120 days (cattle) inhibited larval development of several species in dung deposited up to 199 days post-treatment [186-187]. Slow release capsules of IVM developed for sheep also had an extended impact of the development and survival of two species of paracoprid beetle (*Onthophagus taurus* and *Euoniticellus fulvus*) [179].

Single standard injection of cattle with 200 µg IVM kg^-1^ body weight reduced the number of brood balls by *Euoniticellus intermedius*, and reduced the emergence up to day 14 post-treatment, with 0 to 3% survival from day 2 to day 14 [188]. In *Onitis alexis*, reduced emergence was observed on days 2 to 7, and a prolonged development up to day 21 post-treatment [188]. Similar effects were obtained with *Euoniticellus fulvus* [100, 173], *Diastellopalpus quinquedens* [189], *Digitonthophagus gazella* [193-195] and *Onthophagus taurus* [192].

In the same way that has been developed for Diptera, a standardised test has been developed for dung beetles. The advisory group DOTTS (Dung Organism Toxicity Test Standardization) of the Society for Environmental Toxicity and Chemistry (SETAC) decided to develop tests with dung beetles, including the temperate species *Aphodius constans*. In the *A. constans* test, the survival of larvae was determined after exposure to four veterinary parasiticides (IVM, MOX, dicyclanil, and praziquantel) representing different treatment regimes, modes of action, and effect levels [193-195]. IVM was the most toxic substance (median lethal concentration [LC_50_] = 0.9 - 1.0 mg of active ingredient per kilogram of dung dry weight [mg IVM kg^-1^ dung (d.w.)] followed by dicyclanil (LC_50_ = 1.5-6.0 mg dicyclanil kg^-1^ dung [d.w.]) and MOX (LC_50_ = 4.0 - 5.4 mg MOX kg^-1^ dung [d.w.]), whereas praziquantel showed very low toxicity (LC_50_ >1,000 mg praziquantel kg^-1^ dung [d.w.]). The toxicity in fresh and formulated dung differed by a factor of between 1.1 and 4. NOEC values were as low as 0.3 mg IVM kg^-1^ dung (d.w) [193-194]. In another test using the same method, an LC_50_ of 0.5 mg IVM kg^-1^ dung (d.w.) and a NOEC of 0.3 mg IVM kg^-1^ dung (d.w.) was determined for the related species *Aphodius fimetarius* (Dagmar Thauer, ECT Oekotoxikologie, Flörsheim, Germany – personal communication). In five tests with dung from treated cattle performed in parallel with the work reported here, very similar LC_50_ values were found: 0.5 to 0.8 mg IVM kg^-1^ dung (d.w.) [195]. The LC_50_ using dung directly obtained from treated cattle ranged from 0.5 to 0.7 mg IVM kg^-1^ dung (d.w.) and 0.07 to 0.1 mg IVM kg^-1^ dung (fresh weight; f.w.). Using mixtures, the outcome of two tests was almost identical: 0.77 to 0.78 mg IVM kg^-1^ dung (d.w.); 0.11 to 0.13 mg IVM kg^-1^ dung (f.w.). In comparison to the LC_50_ values obtained when IVM was spiked in control dung at several concentrations (LC_50_ = 0.9-1.0 mg IVM kg^-1^ dung, d.w.), the LC_50_ values were again very similar [195].

#### 4.2.2 Soil Organisms

##### 4.2.2.1 Earthworms

Earthworms can be important dung decomposers in pastureland [196-197] and several studies have focused on effects of residues and metabolites of IVM on earthworm populations associated with dung pats in the field. Other studies were concerned with IVM toxicity under controlled laboratory conditions. Earthworm fecundity and mortality were investigated at different concentrations of IVM in dung provided as food [198-199] and in the soil [70, 200] (Table **3**). IVM appeared toxic at high concentrations in the artificial soils but showed no adverse effects on earthworm growth and survival at the low levels typically observed on pastures [177, 198-199]. The effects of residual IVM on earthworm activity and dung decomposition (*Pheretima heteropoda* and *P. divergens*) were studied in Japan where artificial cowpats containing 0, 0.1 and 1 mg IVM kg^-1^ dung were deposited on grassland [201]. Earthworms aggregated around the pats regardless of the concentration of IVM and no difference in degradation rates was detected. These results are in accord with previous studies reporting the apparent absence of adverse effects of IVM on earthworm activity [175, 177, 202-204]. Svendsen *et al.* [204] investigated the long term effects of IVM on earthworm populations and dung pat decomposition in two grazing seasons in Denmark. IVM excreted by heifers treated with a sustained release bolus had no negative impact on earthworm populations, worm biomass, or species composition.

The effects of the mixture of 94% ivermectin B_1a_ and 2.8% ivermectin B_1b_ on soil invertebrates have been investigated in laboratory tests on three soil invertebrate species: the earthworm *Eisenia fetida*, the springtail *Folsomia candida*, and the predatory mite *Hypoaspis aculeifer* [205] (values in Table **3**). The effects of IVM on reproduction started at a concentration of 5 mg kg^-1^ soil (d.w.), and reproduction was reduced to 10% of control levels at 10 mg kg^-1^ soil (d.w.). These values have to be compared with those obtained for *E. fetida* by Sun *et al.* with avermectin B_1a _(= abamectin) [206]. The 7-days LC_50_ and 14-days LC_50_ values were 24.1 and 17.1 mg kg^-1^ soil d.w., respectively [206]. In the Oligochaeta *Enchytraeus crypticus*, with 21-days exposure, the EC_10_ and EC_50_ values were 14 mg kg^-1^ soil (d.w.) within the concentration range tested (0-300 mg kg^-1^ d w ) [207].

##### 4.2.2.2 Springtails

For the collembolan *Folsomia candida*, 36% mortality started for adults at 3.2 mg.kg^-1^ soil (d.w.) and no springtails survived at the highest test concentration of 100 mg IVM kg^-1^ soil (d.w.) [205]. The LC_50_ value was 12.4 mg kg^-1^ soil (d.w.). Reproduction was impacted at lower concentrations, with NOEC and LOEC values for reproduction of 0.3 mg kg^-1^ soil (d.w.) and 1.0 mg kg^-1^ soil (d.w.), respectively and a EC_50_ for reproduction of 1.7 mg kg^-1^ soil (d.w.) [0.8-3.4 mg kg^-1^ soil (d.w.)]. The ACR (acute-to-chronic ratio) between LC_50_ and NOEC was 41.3 in the test with *F. candida*. The tests revealed a high sensitivity of the collembolan *F. candida* to IVM as shown by a NOEC of 0.3 mg kg^-1^ soil (d.w.). Very similar results were found in another collembolan species, *Folsomia fimetaria,* with NOEC of 0.3 [207] and 0.4 mg kg^-1^ soil d.w. [208], with an ACR between LC_50_ and NOEC with *F. fimetaria* of about 28 [197] and 13 [208].

##### 4.2.2.3 Mites 

The mortality of adults of the predatory mite *Hypoaspis aculeifer* exposed to IVM occurred at the highest test concentration of 31.6 mg kg^-1^ soil (d.w.) (33% mortality) [205]. Reproduction was affected at only the next lowest test concentration such that the NOEC and LOEC values for the end-point reproduction were determined to be 3.2 mg kg^-1^ soil (d.w.) and 10.0 mg kg^-1^ soil (d.w.), respectively, with EC_50_ for reproduction of 17.8 mg kg^-1^ soil (d.w.) [15.4 - 20.8 mg kg^-1^ soil (d.w.)].

##### 4.2.2.4 Nematodes

Yeates *et al.* investigated soil nematodes beneath faecal pats from IVM-treated cattle over 3 years by depositing fresh pats regularly on the same soil spots [209]. Adverse effects of IVM on abundance were found only for a few taxa, but not consistently over the entire study period. Similarly, another study found no effect of faeces from IVM-treated reindeer on total soil nematode abundance [210]. However, significantly lower total abundances of soil nematodes were found beneath faecal pats from IVM-treated sheep [211]. The population growth of the soil nematode *Pristionchus maupasi* was significantly reduced to below zero at a concentration of 5 mg IVM kg^-1^ faeces (w.w.) compared to the density in control [212] (Table **3**).

#### 4.2.3 Aquatic Organisms

The effects of MLs on aquatic organisms have been exhaustively reviewed by Kövecses and Marcogliese [22] and by Brinke *et al.* [213]. Due to strong binding of IVM to soil [214] and, thus, little potential for transport from the terrestrial to the aquatic compartment, no risk for aquatic organisms was indicated in previous environmental risk assessments of IVM [74, 120, 156]. As a result, very few studies have been undertaken to examine the adverse impacts of exposure to IVM on freshwater organisms. To date, however, *Daphnia magna *has the lowest LC_50_ of all organisms tested [22, 215-216], while the freshwater oligochaete *Lumbriculus variegatus*, which has been tested for lethal and sublethal effects was found with a LC_50_ (72 h) of ≈ 0.5 mg L^-1^ [217] and a NOEC (56 days) of 0.2 mg kg^-1^ sediment (d.w.) [218] (Table **3**). Benthic cladocerans are very sensitive to IVM, as reported for pelagic species. Halley *et al.* [70] have already noted that acute toxicity of IVM for *Daphnia magna *occurs at concentrations as low as 25 ng L^-1^, but recent studies have found that even lower concentrations yield acute (5.7 ng L^-1^) or chronically (1 pg L^-1^) toxic effects [215]. *Ceriodaphnia dubia *was shown to be less sensitive than *D. magna*, but its growth and reproduction were nonetheless significantly affected at a concentration of 0.01 ng L^-1^ [219].

IVM has a distinct impact on nematodes, leading to significantly lower abundances at concentrations of 6.2 μg kg^-1^ d.w. and 31 μg kg^-1^ d.w. A single species toxicity test, using the free-living nematode *Caenorhabditis elegans*, revealed a NOEC of 100 μg kg^-1^ d.w. for reproduction [58, 213]. Indoor microcosms were used to assess the impact of IVM on freshwater meiobenthic communities over a period of 224 days. IVM significantly altered meiobenthic communities, with pronounced effects on benthic microcrustaceans (cladocerans, ostracods) and nematodes. The most pronounced effects on the meiofauna community composition occurred at the highest treatment level (31 μg kg^-1^ d.w.), leading to a no observed effect concentration (NOECCommunity) of 6.2 μg kg^-1^ d.w. [213].

Indoor microcosms were used to assess the impact of IVM on freshwater meiobenthic communities over a period of 224 days. IVM significantly altered meiobenthic communities, with pronounced effects on benthic microcrustaceans (cladocerans, ostracods) and nematodes. The most pronounced effects on the meiofauna community composition occurred at the highest treatment level (31 μg kg^-1^ d.w.), no observed effect concentration (NOECCommunity) of 6.2 μg kg^-1^ d.w. leading to a no observed effect concentration (NOECCommunity) of 6.2 μg kg^-1^ d.w. [213].

The aquaculture industry uses IVM as an alternative chemotherapeutic treatment for ectoparasitic copepods, also known as sea lice [22]. Its use in the aquaculture industry is “off-label”; use in fish is not recommended by the manufacturer, but veterinarians are still allowed to prescribe food treated with IVM under “emergency situations,” although in recent years, emamectin benzoate (another avermectin) is increasingly used to treat lice infestations in salmon. The ecotoxicological effects and persistence of IVM in terrestrial ecosystems has raised significant concerns among researchers and the public about its use in marine environments [220]. Subsequently, studies have been undertaken to measure the potential impacts on target and non-target fauna in marine systems. Grant & Briggs studied the toxicity of IVM to several estuarine and marine invertebrates [221]. The LC_50_ values varied from 0.03 to more than 10,000 µg L^-1^ (Table **3**). The most sensitive organisms were the mysid *Neomysis integer* and the amphipod *Gammarus* spp. Toxicity thresholds for these species were as low as 0.03 µg L^-1^. These values are an order of magnitude lower than those of *Daphnia magna*, *Crangon septemspinosa* (invertebrates) and *Salmo gairdneri* and *Lepomis macrochirus* among fish [70, 162, 222], and are comparable with those reported for abamectin [120].

Molluscs and nematodes have the highest LC_50_ values, but sublethal effects on the behaviour of *Littorina littorea* were observed at low concentrations [223] (Table **3**). In bivalvia, LC_50_ ranged from 80 to 430 µg kg^-1^ [223], whereas the distribution of LC_50_ values in gastropoda is much broader (30 to >10,000 µg L^-1^) [167,220-221,223-224].

Due to strong binding of IVM to soil particles [214], sediment dwelling and benthic organisms are particularly exposed to IVM. *Asterias rubens *(Echinoderm) presented a 10-days LC_50_ of 23.0 mg kg^-1^, and an exposure to 20 mg IVM kg^-1^ sediment (d.w.) significantly reduced the ability of *A. rubens *to right itself [225]. Reductions in the rate of cast production by *Arenicola marina *were measured at all test concentrations (≥ 0.006 mg kg^-1^ sediment, d.w.). The LC_50_ value (10 days exposure to IVM) was of 0.023 mg kg^-1^ sediment d.w. and prior exposure to IVM significantly reduced its ability to rebury itself in clean sediment [226] (Table **3**).

#### 4.2.4 Ecotoxicity of Other Avermectins

Eprinomectin (EPR) and doramectin (DOR) have been registered against all stages of the major gastrointestinal nematodes, lungworm, as well as lice, horn fly, ticks and mange mites of cattle [227]. Both of these MLs have significant persistent activity against a range of important nematodes. EPR is the most recent member of the avermectin class of MLs and was selected for development as a topical endectocide in cattle after examination of several hundred analogues because it possesses the most potent broad-spectrum activity against nematodes [228]. The toxicity of these MLs has been evaluated and many end-points obtained for these compounds for plants, invertebrates and vertebrates (Tables **4-6**).

### 4.3 Ecotoxicity of Eprinomectin

A summary of ecotoxicology data for eprinomectin is provided in Table **4**. 

EPR residues have been shown to have adverse effects on the survival of dung-feeding diptera for periods of 1-4 weeks after treatment [9-10,178,180,231]. The treatment of cattle with a topical application of EPR at the dosage of 500 μg kg^-1^ l.w. suppressed the development of horn fly (*Haematobia irritans*) for at least 4 weeks post-application. Suppression of stable fly (*Stomoxys calcitrans*) and house fly *Musca domestica* ranged from 1 to 5 weeks [178]. Faeces voided by cattle treated with EPR were also associated with high larval mortality of *Neomyia cornicina* during the first 12 days after treatment, with null emergence until day 7. The NOEC for *N. cornicina *was estimated to be close to 7±5 µg kg^-1^ [231]. Survival of larvae of two tropical species of dung breeding fly (*Musca inferior* and *Orthelia timorensis*) was reduced for 1-2 weeks post-treatment in pats voided by cattle treated with a topical dose of EPR [180].

The toxicity of EPR was first determined on two species of dung beetle, *Digitonthophagus gazella* and *Euoniticellusintermedius* [230]. No live progeny were recovered at the 166 or 590 µg kg^-1^ levels on a wet-weight basis. The NOEC, based on numbers of emerged progeny relative to pooled controls (untreated and solvent controls), was 64.7 µg kg^-1^ for both species. Faeces voided by cattle treated with a pour-on formulation of EPR were associated with high juvenile mortality (larvae) of the dung beetle *Onthophagus taurus* during the first 1-2 weeks after treatment [231]. Increased mortality also occurred among newly emerged beetles fed on faeces collected 3 days after EPR treatment and there was evidence of suppressed brood production among those that survived. This effect was still apparent even after insects fed for a further 10 days on the faeces of untreated cattle. A 3-year study performed to assess the effect of endectocide residues on the attractiveness of cattle dung to colonizing insects showed that EPR tended to repel insects, contrary to IVM and MOX which showed a strong attractive effect [232-233]. Suárez *et al.* [234] also found that dung voided by cattle treated with EPR attracted fewer beetles than the dung of untreated animals.

The 28-day toxicity (LC_50_) value for earthworms exposed to EPR in an artificial soil was greater than 951 mg EPR kg^-1^ dry soil, the highest concentration tested, while the NOEC was 295 mg EPR kg^-1^ dry soil [235]. These levels are higher than the levels expected in faeces from dosed cattle or in soil fertilised with manure from dosed cattle, which indicates a wide margin of safety for this compound to earthworms. However, the worms exhibited a dose-dependent weight loss at all test concentrations, so the no-observed-effect concentration was below the lowest level tested in this study, 90.8 mg kg^-1^ dry soil. Wall and Strong [202] and Madsen *et al. *[177] also found no effects on earthworms in field and laboratory studies of dung from cattle after treatment with IVM. Toxicity of eprinomectin B_1a_ (the major component of EPR) to *Lumbricus terrestris* was carried on under conditions mimicking typical product use on pasture (concentrations between 0 and 0.01 mg kg^-1^ dry soil, weight basis) [236]. No significant differences (p > 0.05) were observed at any day post-treatment in the survival or behavioural effects of any worms fed post-dose faeces relative to the worms fed control faeces. None of the post-dose comparisons of weight changes of living earthworms to the control group were significantly different (p > 0.05), indicating that treatment of cattle with EPR did not affect feeding or weight gain of earthworms. The LC_50_ value and the results of this study established the wide margin of safety afforded to earthworms by EPR under typical usage conditions.

The acute toxicity of EPR to the cladoceran *Daphnia magna* was based on the mortality/immobility data for 24 and 48h of exposure of daphnids to EPR. The 48h EC_50_ value was 0.45 (0.37 - 0.64) µg a.i. L^-1^ while the 48-h NOEC was less than 0.37 µg a.i. L^-1^, the lowest concentration tested [230].

### 4.4 Ecotoxicity of Doramectin

A summary of ecotoxicology data for DOR is provided in Table **5** [7, 240]. When compared to dihydroavermectin B_la_, the major component of IVM, doramectin (DOR) displays favorable intrinsic activity and duration of efficacy in preventing the establishment of nematode infections in cattle [237]. A lower clearance, a lower volume of distribution and, probably, a higher bioavailability of DOR over IVM may explain the differences [238]. A study compared the faecal elimination profile of DOR after oral or intramuscular (IM) administration in horses (oral dose of 0.2 mg DOR kg^-1^ b.w.; IM route of 0.2 mg DOR kg^-1^ b.w.) [239]. In horses treated orally, the peak faecal concentration (FC_max_) was 2.3 ± 0.6 mg kg^-1^ observed at 1.9 ± 0.5 days after treatment, whereas, for those treated by the IM route, the FC_max_ was lower (0.16 ± 0.03 mg kg^-1^) and it was observed at 5.6 ± 2.9 days. Such results can explain the differences in toxicity observed to non-target organisms feeding on faeces of treated animals.

No mortality was observed in the earthworm *Eisenia fetida* exposed to 1,000 mg kg^-1 ^DOR in an artificial soil for 28 days. The 28 day LC_50_ is therefore > 1,000 mg kg^-1^. Based on weight gain, the most sensitive criteria monitored, the NOEC was 2 mg kg^-1^ and the LOEC was 4 mg kg^-1^ [240]. Kolar *et al.* investigated the toxicity of DOR to soil invertebrates in soil and in faeces from recently treated sheep [146]. In soil, the LC_50_ for earthworms (*Eisenia andrei*) was 228 mg kg^-1^ dry soil, while LC_50s_ were >300 mg kg^-1^ for springtails (*Folsomia candida*), isopods (*Porcellio scaber*) and enchytraeids (*Enchytraeus crypticus*). EC_50s_ for the effect on reproduction of springtails and enchytraeids were 42 and 170 mg kg^-1^, respectively. For earthworms, NOEC was 8.4 mg kg^-1^ for DOR effects on body weight. When exposed in faeces, springtails and enchytraeids gave LC_50s_ and EC_50s_ of 2.2 to >2.4 mg kg^-1^ for DOR. Earthworm reproduction was not affected [146].

A study was conducted to evaluate the insecticidal persistence in dung of DOR administered topically to cattle at a dosage of 500 μg kg^-1^ (= 0.1 mL of medicinal product for every 1 kg body weight) against two dung beetles (*Euoniticellus intermedius* and *Digitonthophagus gazella*) and a predaceous staphylinid (*Philonthus flavolimbatus*) [240]. Bioassays conducted in the laboratory showed that *E. intermedius* and *D. gazella* produced significantly fewer progeny when exposed to faeces collected from cattle 7 and 14 days after treatment with DOR pour-on compared with exposure to faeces collected from control cattle. The LC_50_ and LC_90_ of DOR for immature *D. gazella* were approximately 12.5 µg kg^-1^ and 38.2 µg kg^-1^, respectively; concentrations up to 250 µg kg^-1^ had no effect upon number of brood balls produced by mating pairs. 

Reduction of progeny was observed at day 7 for *P. flavolimbatus* and until day 14 for *E. intermedius* and for *D. gazella, *indicating that residues excreted in dung during this time period were present at concentrations that affected beetle development. Larvae of dung feeding flies, mainly *Ravinia* spp., *Neomyia cornicina *and *Musca autumnalis *were reduced in pats voided by DOR-treated cattle [240]. Similar effects were noted when DOR was used on cattle to test its efficacy against Old World screw worm fly (*Chrysomya bezziana*). The cattle remained myiasis-free for 1 week and their pats supported no fly larvae for 9-13 days post-treatment [180]. DOR pour-on (formulation 0.1 mL kg^-1^ b.w) has also been shown to reduce the survival of larvae of *Musca domestica*, *Haematobia irritans* (horn fly) and *Stomoxys calcitrans* for at least 1 to 4 weeks after treatment [178]. In the case of horn fly larvae, the LC_50_ and NOEC for egg to adult emergence were *ca.* 3 µg L^-1^ and 2.4 µg L^-1^ respectively [240].

The susceptibility of vertebrates (fish) is less than for invertebrates. Acute toxicity of DOR for bluegill sunfish *(Lepomis macrochirus)* and rainbow trout *(Onchorhynchus mykiss) *was measured under static conditions. The 96h LC_50_ and the NOEC were of 11 and 2.3 µg kg^-1^, respectively for *L. macrochirus *and of 5.1 and 2.5 µg kg^-1^ for *O. mykiss* [240]. Acute toxicity of DOR for the water flea *Daphnia magna *was measured under static conditions. The 48h EC_50_ concentration and NOEC are 0.10 µg L^-1^ and 25 ng L^-1^, respectively [240]. 

### 4.5 Ecotoxicity of Selamectin

Selamectin is a semisynthetic monosaccharide oxime derivative of DOR which combines anti-arthropod with anti-nematodal activity. It is active against nematodes (heart-worm *Dirofilaria immitis*), fleas (*Ctenocephalides felis felis*), ear mites (*Otodectes*), sarcoptic mange (*Sarcoptes scabei*) and ticks in dogs and cats [241-242]. The efficacy and safety of selamectin used off-label in exotic pets have been recently reviewed [243].

Few studies were published on ecotoxicology of this ML. In a sediment/water toxicity study with *Daphnia* sp., the PEC was of 20.3 ng L^-1^ with a PEC/PNEC ratio of 0.85 [244]. Under static renewal conditions, the calculated 48-h EC_50_ was 26 (23 – 35) ng L^-1^for *D. magna*, with a 48-h NOEC of 7.1 ng L^-1^. Under static conditions in the presence of freshwater sediment, the 48-h EC_50_ value for *D. magna* in the sediment:water system was 0.24 µg L^-1^, with a NOEC of 0.073 µg L^-1^. For the mysid shrimp, the 96-h LC_50_ was 28 ng L^-1^, while for sheepshead minnow the 48-h LC_50_ was > 500 µg L^-1^ and for *Selenastrum capricornutum* NOEC > 763 µg L^-1^. 

### 4.6 Ecotoxicity of Emamectin Benzoate

A summary of ecotoxicology data for emamectin benzoate (EMB) is provided in Table **6**. Emamectin benzoate [(4″R)-4″-deoxy-4″-(methylamino) avermectin B_1_ benzoate] is a synthetic analogue of abamectin developed for the control of insect pests [245]. EMB is widely used in fish farms as it is an effective therapeutic agent against juvenile, chalimus, and motile pre-adult and adult life stages of sea lice whilst being less toxic to salmon than IVM [246]. A review of characteristics of this ML was deposed for the dossier of Slice®, a formulation of EMB against sea lice [247].

#### 4.6.1 Aquatic Plants

Micro-algae are not sensitive to avermectins and no effects were detected at the highest concentrations of EMB tested with *Selenastrum capricornutum *over 5 days, 3.9 μg L^-1^ [246-247]. *Lemna gibba* (duckweed), exposed to EMB at concentrations up to 94 μg L^-1^ for 14 days was not affected although higher concentrations were detected in the plants than in the test solutions [246].

#### 4.6.2 Aquatic Invertebrates

The sensitivity of invertebrates to emamectin benzoate differs markedly between and within phyla with marine crustaceans being the most sensitive. The lowest toxicity value reported for animals exposed in water is the 96h LC_50_ value of 0.04 μg L^-1^ reported for *Mysidopsis bahia. Crangon crangon *and *Nephrops norvegicus *were affected by EMB in water, with 192h LC_50_ values of 161 and 572 μg L^-1^, respectively [247]. There were no significant effects or mortalities in *C. crangon *or *N. norvegicus *observed for 192h and fed for 8 days on fish feed treated with EMB at concentrations up to 69.3 and 68.2 mg kg^-1^ respectively [247].

EMB is the only medicinal product allowed since 2000 by official authority in Chile for control of the ectoparasitic copepod *Caligus rogercresseyi*, the most important parasite of farmed salmonids in Chile. The sensitivity of *C. rogercresseyi* to EMB was studied in 18 salmon farms [248]. Sensitivity values, recorded as EC_50_ (immobilization) in adults, were between 57 and 203 µg L^-1^ (µg kg^-1^) in the summer season, and between 202 and 870 µg kg^-1^ for the winter season. The EC_50_ control value, obtained from naïve *Lepeophtheirus mugiloidis* parasites, was 34 µg kg^-1^. Willis and Ling [249] investigated the acute and sublethal toxicity of emamectin benzoate to non-target planktonic marine copepods. The comparative sensitivity of three life stages (nauplii, copepodites, adults) of *Acartia clausi*, *Pseudocalanus elongatus*, *Temora longicornis* and *Oithona similis* was assessed in 48-h exposures followed by a recovery period in toxicant-free sea water. The calanoid copepods responded similarly to EMB and EC_50_ values were significantly lower than those for the cyclopoid *O. similis*. Nauplii and copepodite 48h EC_50_ values were generally lower than those for the adults. EC_50_ values ranged from 0.12 µg L^-1^ (*P. elongatus* nauplii) to 232 µg L^-1^ (*O. similis* adults). The primary toxic effect, immobilization, was generally irreversible. A 7-day sublethal test with adult *A. clause *females measured a significant reduction in egg production at higher concentrations. The NOEC and LOEC values were 0.05 and 0.158 µg L^-1^, respectively. Authors concluded that concentrations causing toxicity to planktonic copepods were considerably higher than Predicted Environmental Concentrations (PEC) in the vicinity of treated salmon farms and suggest that the use of emamectin benzoate for lice control is unlikely to adversely affect planktonic copepods [249]. However metabolites of the ivermectins/avermectins are generally less toxic than the parent compounds [250] and it has been found with *Acartia tonsa* (Copepod) that the desmethylamino metabolite of EMB was approximately 36% less toxic than the parent compound [247].

High doses of EMB can disrupt the molt cycle of ovigerous American lobsters (*Homarus americanus*), causing them to enter proecdysis prematurely and lose their attached eggs when the shell is cast [96]. The greatest risk of benthic crustaceans being exposed to EMB is from the consumption of EMB-medicated fish feed or fish faeces, as the drug is not found in the water column and does not bioaccumulate in animals nor biomagnify in the food chain [247]. Densities of American lobsters and other crustaceans can be disproportionately high near salmon cages [251-253] and waste fish feed may be providing a food supply that attracts lobsters to farm sites [254]. To determine the dose response to EMB, lobsters were forced to ingest doses that ranged from 0.05 to 0.39 mg kg^-1^ body weight [255]. A significant proportion of lobsters given doses of 0.39 and 0.22 mg kg^-1^ (37% and 23%, respectively) moulted prematurely, almost a year earlier than the control group. All the lobsters in the 0.05 and 0.12 mg kg^-1^ groups moulted at the normal time and the mean time of moult was similar to that of the control group. The NOEL and lowest-observed-effect level (LOEL) of EMB on the moult cycle were 0.12 and 0.22 mg EMB kg^-1^ lobster, respectively. To acquire the LOEL, a 500-g lobster would have to consume 22 g of salmon feed medicated with EMB at a level of 5 mg EMB kg^-1^ feed [255].

The oyster, *Crassostrea virginica, *is markedly less sensitive to EMB than the most sensitive crustaceans, with an estimated LC_50_ value of 0.7 mg L^-1^ and an EC_50_ for shell deposition of 0.5 mg L^-1^ [247]. The binding characteristics of EMB indicate that the organisms which will come into contact with the highest concentrations are the sediment reworkers and epibenthic scavengers. The annelid *Arenicola marina* and the amphipod *Corophium volutator* have similar sensitivities with 10 day LC_50_ values of 0.1 and 0.2 mg kg^-1^ (wet weight) sediment respectively, although polychaetes, *Capitella capitata*, collected from beneath salmon farms were markedly less sensitive with a 21 day LC_50_ value of 1.0 mg.kg^-1^ determined in microcosms [247] (Table **6**). Experimental data on tissue concentrations enable an assessment to be made of EMB levels which might be considered lethal to invertebrates. Concentrations in animals surviving exposure to water concentrations above the respective NOEC values for *Crangon *and *Nephrops *of 0.16 and 0.8 mg L^-1^ were 0.14 and 0.10 mg kg^-1^. In the case of *Nephrops *the tissue concentration in animals surviving exposure to the NOEC of 0.4 mg L^-1^ was 0.07 mg kg^-1^. Concentrations as high as 0.2 and 0.14 mg kg^-1^ were determined in *Nephrops *and *Crangon *surviving exposure to 1.5 and 0.16 mg L^-1^ respectively [246]. It appears that both animals can tolerate exposure to EMB which gives rise to edible tissue concentrations of *ca. *0.07 mg kg^-1^. The similarity of the apparently lethal body concentrations in the two crustaceans and the scale of the difference in the water NOEC values may be related to differences in the metabolic degradation of EMB [246].

#### 4.6.3 Terrestrial Invertebrates

Emamectin benzoate is one of the newer MLs and was developed for the control of insect pests. However, there are already reports of resistant populations. Argentine *et al.* studied the sensitivity of six species of Lepidoptera to EMB [256]. The LC_90_ values for emamectin benzoate ranged from 5 to 22 μg EMB L^-1^, while LC_50_ varied from 3 to 1 μg EMB L^-1^ (Table **6**). There was a 10-fold difference in LC_50_ values between the least sensitive species of this study and the most sensitive laboratory population (Lab-PK) of *Spodoptera litura*, a serious crop pest in Pakistan and elsewhere. In Pakistan* Spodoptera litura* (Lepidoptera Noctuidae) developed a possible cross-resistance between emamectin benzoate and other insecticides, with a LC_50_ ranging between 0.03 mg EMB L^-1^ for the susceptible laboratory population (Lab-PK) and 2.3 mg EMB L^-1^ for field populations [144] (Table **6**). Out of 19 field populations tested, five showed moderate level of resistance (resistance ratio ranging from 15- to 21-fold greater than the most sensitive laboratory strain, Lab-PK), whereas 11 populations were tolerant to EMB (resistance ratio of 2 – 10-fold only). Three populations revealed high levels of resistance, with resistance factor of 33 to 77- fold.

#### 4.6.4 Vertebrates

Salmon fed with emamectin benzoate in medicated feed at up to 356 μg kg^-1^ fish day^-1^ for 7 days exhibited no mortality with a NOEC of 0.17 mg kg^-1^ fish day^-1^ [246]. In acute toxicity studies three freshwater fish species exhibited similar sensitivities, with 96h LC_50_ values of 0.17-0.19 mg L^-1^ of water, while *Cyprinodon variegatus* (sheepshead minnow, a marine fish) was markedly less sensitive with an LC_50_ value which was eight-fold greater, 1.35 mg L^-1^ (Table **6**). Exposure of bluegill sunfish to 1.2 μg L^-1^ for 28 days resulted in no signs of toxicity [257]. NOEC and MATC (Maximum Acceptable Toxicant Concentration) values of 12 and 18 μg L^-1^ respectively have been determined in an early life stage study with fathead minnow. As with invertebrates, fish appear more sensitive to IVM than they are to EMB.

Toxicity data for mallard duck (*Anas platyrhynchos*) showed them to be at least twice as sensitive as bobwhite quail (*Colinus virginianus*) by both the acute oral (LC/LD_50_ of 76 and 264 mg EMB kg^-1^, respectively, and NOEC mortality of 25 and 100 mg EMB kg^-1^, respectively) and dietary routes (LC/LD_50_ of 570 and 1,318 mg EMB kg^-1^, respectively, and NOEC mortality of 80 and 500 mg EMB kg^-1^, respectively) [258]. No effects on reproduction were obtained on either species at the highest concentrations tested, 40 and 125 mg kg^-1^ for the mallard and bobwhite, respectively [246].

### 4.7 Ecotoxicity of Aversectin C

Aversectin C (the letter “C” stands for “complex”) is a purified mixture of eight naturally occurring avermectins extracted from the biomass of *Streptomyces avermitilis*, with the following composition: A_1a_ - 9%; A_1b_ - 4%; B_1a_ - 36%; B_1b_ - 6%; A_2a_ - 19%; A_2b_ - 4%; B_2a_ - 21%; B_2b_ - 1%. Aversectin C is mostly used in Russia and neighbouring countries, both for veterinary use (endo- and ectoparasites) and crop protection (control of Colorado potato beetle, spider mite, melon and peach aphids, tobacco and California thrips and other leaf miners and leaf-sucking pests in both field and greenhouse) [259-260]. 

Few ecotoxicological data are available on this compound. The acute oral, cutaneous, and inhalation toxicity of aversectin C was studied on rats and mice. The compound was less toxic for rats than for mice, the LD_50_ for oral administration being 90 and 33 mg kg^-1^, respectively. Aversectin C exhibited maximum acute toxicity following inhalation in rats (LD_50_ = 40 mg kg^-1^), while a minimum toxicity level was observed for the cutaneous application in rats (1,700 mg kg^-1^) [261]. Aversectin C inhibits thymocyte apoptosis of rat. The IC_50_ dose was in the range of 0.1-0.3 mg L^-1^ for aversectin C, whereas abamectin and IVM produced no effect up to 1 mg L^-1^ [262]. We are unaware of any studies that have assessed the non-target effects of aversectin C residues in the environment.

## 5 ECOTOXICITY OF MILBEMYCINS

Much less information is available on the ecotoxicity of milbemycins than for avermectins. Of the former group, virtually all information is limited to moxidectin (MOX) – primarily for studies on dung-dwelling invertebrates. A summary of ecotoxicology data for MOX is provided in Table **7**.

### 5.1 Diptera

In one of the earliest studies, residues in dung of cattle treated 2 days previously with a recommended topical dose of MOX (0.5 mg kg^-1^ b.w.) did not affect numbers of cyclorrhaphan Diptera, but the authors viewed this result with caution due to the very low numbers of larvae present in the control treatment [263]. However, the relatively low toxicity of MOX to dung-dwelling dipterans since has been documented in several other studies. 

Larval survival of house fly (*Musca domestica*) was unaffected by residues in dung of cattle dosed with MOX 2-7 days previously [174, 178, 264] or in dung of swine treated 9 days previously [264]. Residues did not affect the survival of bush fly (*Musca vetustissima*) in dung of cattle treated 2 days previously [174] or stable fly (*Stomoxys calcitrans*) in dung of cattle treated 7 days previously [178]. Larvae of the closely-related buffalo fly (*Haematobia irritans exigua*) and horn fly (*H. irritans*) appear to be more susceptible. In an extreme example, reduced survival of horn fly was reported in dung from cattle treated 28 days previously with an injectable dose of MOX [265], but this result has not since been repeated. Topical applications of MOX reduced survival of *H. irritans* larvae in dung from cattle treated one, but not 2 weeks, previously [178, 266]. Although concentrations of ≤ 64 µg kg^-1^ MOX in spiked dung did not affect pupation by larvae of *H. i. exigua* [66], such concentrations are likely to be exceeded in dung of cattle for 1-4 days after treatment with MOX applied in injectable dose [267]. Larval survival of *Neomyia cornicina* has been reported to be reduced for 7-14 [266] and 10-16 days [268] after MOX treatment. 

### 5.2 Dung Beetles

The residual toxicity of MOX in dung mainly has been tested for two species of dung beetles; i.e., *Euoniticellus intermedius* and *Digitonthophagus gazella*. For both species, residues present in dung of cattle treated 1-42 days previously with MOX in an injectable [269] or topical formulation [270], had no effect on reproductive success. MOX added directly to fresh cattle dung had no effect on the fecundity of *D. gazella* at concentrations of 4-512 µg kg^-1^, and only reduced survival of larvae at concentrations of 256-512 µg kg^-1^ [66]. Residues in dung from cattle dosed topically withMOX 3-70 days previously for tests on *Onthophagus taurus *[179], or 1-28 days previously for tests *Caccobius jessoensis *[266], had no detectable effect on the reproduction of either species. Reduced larval survival of *Aphodius constans* was not detected in dung of sheep orally-treated with MOX beyond 2 days post-treatment [268], and no effect of residue on the survival of *Aphodius* larvae was detected in dung from cattle treated 2-14 days previously with MOX in an injectable formulation [263].

## 6 ECOTOXICITY OF SPINOSYNS 

The insecticidal properties of the spinosyns were first identified in a qualitative mosquito bioassay conducted as part of a soil-sample screening program for biologically active compounds [271]. A summary of ecotoxicology data for spinosad (SPI) is provided in Table **8**. This insecticide is used for the control of caterpillars [272-274,317], thrips [275], beetle and fly pests in a range of fruit and vegetable crops [276-278], ornamentals, turf [279], and stored grains [280-282]. SPI is also used against mosquitos [283-285] and flies [286] as well as tsetse fly [287] control. An oral insecticide formulation of spinosad (tablet formulation) was also developed for treatment and control of adult fleas [288-290]. SPI has contact activity on all life stages of insects, including eggs [291], larvae and adults. Eggs must be sprayed directly but larvae and adults can be effectively dosed through contact with treated surfaces [292]. SPI is most effective when ingested. Foliar applications are not highly systemic, although trans-laminar activity is evident in certain vegetable crops and ornamental plants [293].

Kirst [108] has reviewed the action of spinosyn family of insecticides and their environmental effects. It appears that extensive studies of the effects of SPI on many beneficial species have been published [294-298]. The overall assessment is that the selectivity of the spinosyns against target species is significantly improved over many older insecticides. SPI has a reduced risk to beneficial species when compared with many other insecticides [294-302]. SPI wasclassified by the U.S. Environmental Protection Agency as a reduced-risk material due to its low environmental persistence [303], its moderate toxicity to fish but very little toxicity to birds and mammals [271, 304-306]. The marketing of SPI has focused on its favourable environmental profile, emphasizing its potential for use in integrated pest management systems [306-307]. However laboratory studies indicate that some free-swimming and sediment-dwelling aquatic invertebrates may be sensitive to long-term exposure to SPI [293, 307-309]. Williams *et al.* reviewed laboratory and field studies in order to clearly define the risks to beneficial arthropods posed by SPI use [309]. Authors examined the available information on the impact of SPI on natural enemies and classified mortality responses to SPI using the IOBC laboratory and field scales that run, from 1 (harmless) to 4 (harmful). In total, there were 228 observations on 52 species of natural enemies, of which 162 involved predators (27 species) and 66 involved parasitoids (25 species). The assertion that SPI has little impact on populations of insect natural enemies is probably realistic for predator populations; however certain types of predators are clearly vulnerable to SPI, including earwigs and ants [310]. Overall, 71% of laboratory studies and 79% of field-type studies on predators gave a class 1 result (not harmful). Hymenopteran parasitoids were significantly more susceptible to SPI than predatory insects with 78% of laboratory studies and 86% of field-type studies returning a moderately harmful or harmful result [309]. Predators generally suffer insignificant sub-lethal effects following exposure to SPI, whereas parasitoids often show sub-lethal effects including loss of reproductive capacity and reduced longevity. All studies agree that SPI residues degrade quickly in the field, with little residual toxicity at 3-7 days post application [309].

Cases of resistance to SPI have been reported in various studies, with eventual synergism with other insecticides in *Spodoptera litura* [144]. Resistance was reported in diamondback moths in Hawaii (2000), Georgia (2001), and California (2002) as a consequence of a few years of extensive applications in each region although changes in management practices restored susceptibility, suggesting resistance was reversible [310-313]. Resistance to SPI was associated with microsomal oxidase in *Plutella xylostella* and *Musca domestica* [312-315]. In the western flower thrips *Frankliniella occidentalis* (Thysanoptera: Thripidae), mortality data from reciprocal crosses of resistant and susceptible thrips indicated that resistance was autosomal and not influenced by maternal effects [316].

## 7 ECOTOXICITY OF MLS IN ENVIRONMENTAL COMPARTMENTS AND EFFECTS ON THE WHOLE DUNG COMMUNITY

The physical/chemical properties of MLs indicate that, once they have entered the environment, they can persist for extended periods of time at concentrations high enough to exert toxic impacts. To date, pasture ecosystems have been of greatest concern. In terrestrial systems, the entry of MLs into the environment is through livestock excretion on pasture soils. MLs enter marine systems in the feces of farmed salmon, as well as through uneaten food that settles in sediments [22]. The degradation half-life of ivermectin, in soil or feces-soil mixtures, has been shown to be in the range of 91 to 217 days in the winter and 7 to 14 days in the summer [22, 70, 162] (Table **1**). Lumaret *et al.* [173] reported that ivermectin in dung pats deposited on fields at the end of spring in Spain could no longer be measured after six days, while Sommer & Steffansen [107] reported half-lives of 2.5 to 3 days (pour-on and injection treatments of cattle). In contrast, Madsen *et al.* [177] reported that ivermectin remained active (as measured by toxic impacts on dung fauna) in dung pats for two months and Herd *et al.* [318] reported measurable concentrations of ivermectin up to 50 days post-treatment. Ivermectin has been shown to undergo rapid photodegradation as a thin, dry film on glass with an estimated half-life of 3 h [70, 162] (Table **1**). However reports of low ivermectin persistence in manure following summer or dry conditions might be an artefact resulting from reduced ivermectin extraction efficiency at low moisture content of the solid matrix [67]. Near the surface of open water under clear skies, the half-life of ivermectin is 12 h in summer and 39 h in winter [70].

Tarazona *et al.* [319] analyzed the environmental risk assessment of pharmaceuticals. The screening assessment based exclusively on fate properties, particularly soil adsorption based on the organic carbon adsorption coefficient (K_OC_), is not sufficient in some cases because of the extreme toxicity of some molecules. Ivermectin offers a perfect example; its soil adsorption is very high, resulting in a mobilization potential close to negligible. However, because of its remarkable toxicity, with a chronic NOEC of 0.0003 ng L^-1^ for *Daphnia magna* [215], 10^9^ times below the criteria for highly toxic substances, a potential risk for aquatic bodies after releases into the terrestrial environment cannot be excluded [58]. In grazed ecosystems where rapid recycling of nutrients from the breakdown of dung is a necessary process, it is essential that key organisms be preserved. MLs may enter the terrestrial compartment via spreading of manure from intensively reared animals on arable land or by excretion of dung by animals on pastures [58]. Several models were developed to estimate the environmental burden (predicted environmental concentration, PEC) of MLs that accumulates in environment, both in the terrestrial and aquatic environment [22, 70, 320-321]. Ivermectin was selected as a case study compound within the project ERAPharm (Environmental Risk Assessment of Pharmaceuticals) [58]. The ERA clearly demonstrated unacceptable risks for all investigated environmental compartments and hence suggested the necessity of reassessing ivermectin-containing products. Based on this case study, several gaps in the existing guidelines for ERA of pharmaceuticals were shown and improvements have been suggested [58]. The risk characterization using long-term effects data for aquatic and sediment organisms (*D. magna* and *C. riparius*) as required according to VICH 2004 [51] resulted in an indication of risk for these compartments. While the RQ for sediment organisms was between 2.1 and 36, the RQ for daphnids was >10^5^, indicating a very high risk for aquatic invertebrates [58].

### 7.1 Community Response and Dung Degradation

At least three studies have examined the effects of MOX residues in cattle dung, on assemblages of coprophilous organisms that have colonised dung in the field (Table **9**). In the first study, reductions were observed for six of 19 taxa considered, of which five taxa (2 wasps, 2 beetles, 1 fly) were reduced in dung voided one week after treatment, and one taxon (fly) was reduced in dung voided 2 weeks after treatment with a topical application of MOX [322]. Reductions of the beetle (predators) and wasp (parasitoids) taxa likely reflected reduced numbers of the flies. The second study reported reductions for three of 29 fly taxa considered, in dung voided from cattle topically-treated with moxidectin 1, 7, 14 and 21 days previously (treatments combined for analyses) [266]. In the third study, fewer arthropods (all species combined) were observed in dung of MOX-treated cattle voided 11, but not 21 days, post-treatment [234]. This result mainly reflected the recovery of fewer fly larvae, springtails and mites. Evidence for delayed degradation of dung pats deposited by treated cattle was inconclusive [322], not detected [234], or untested [266].

### 7.2. Comparisons Among Macrocyclic Lactones

The most rigorous comparisons of ecotoxicity are achieved by testing multiple compounds with the same methods at the same time on the same species. Such studies show that the insecticidal activities of avermectin (*i.e.,* ABM, DOR, EPR, IVM) residues are considerably higher than that for MOX. Laboratory bioassays using the larvae of three fly species (*H. irritans*, *M. domestica*, *S. calcitrans*) identified toxicity rankings of DOR > IVM ≈ EPR >> MOX [178]. In a second comparison based on recovery of insects from dung of treated cattle colonised naturally in the field, toxicity rankings were identified as DOR > IVM > EPR >> MOX [322]. In the latter study, use of DOR reduced numbers of several taxa in dung voided at least 4 weeks post-treatment, whereas use of MOX reduced only 1 taxon in dung voided 2 weeks post-treatment. Residues of DOR since have been shown to reduce numbers of some insect taxa in dung of cattle treated up to 16 weeks previously [323].

Other studies support this general rating. In cattle dung, larvae of the fly, *M. domestica*, were unaffected by residues of MOX, but were suppressed by residues of DOR and IVM in dung voided up to 28 days post-treatment [264]. When the study was repeated using swine dung, suppression was observed in dung voided 15 and 11 days after treatment with DOR and IVM, respectively [264]. Numbers of *Aphodius* beetle larvae were reduced in dung of cattle treated 7 days previously with IVM, but not in dung of cattle treated 2 days previously with IVM [263]. Use of DOR caused greater reductions than did MOX, of beetle larvae (mainly *Aphodius* spp.) in dung of cattle treated 3 days previously, and of fly larvae in dung of cattle treated 3-21 days previously [234]. For the beetle, *O. taurus*, larval survival was unaffected by MOX residues in dung from cattle treated 3 days previously, but was reduced by EPR residues in dung of cattle treated 7 days previously [174]. Based on the development of the beetle, *D. gazella*, and the fly, *H. i. exiguae*, in cattle dung spiked with known concentrations of product, abamectin was determined to be about 64-fold more toxic than MOX [66].

In reporting reduced efficacy of MOX against *Gasterophilus* (bot fly) larvae in horses compared with IVM, Xiao *et al.* [324] suggested that MOX may be less lethal to arthropods than IVM, and thus ecologically safer [106]. Fincher and Wang [269] reported that dung from cattle injected with 0.2 mg kg^-1^ MOX had no adverse effects on brood ball production or adult emergence of the dung beetles *D. gazella* and *E. intermedius*, whereas IVM residues reduced adult emergence. Blind field trials in England by Strong & Wall [263] compared the effects of standard injections (0.2 mg kg^-1^) of cattle with MOX and IVM on dung-colonizing insects after spring turnout of livestock. Dung from MOX-treated and control calves showed extensive colonization by *Aphodius* dung beetle larvae at all times, whereas they were unable to colonise dung collected for at least 7 days after IVM treatment.

## CONCLUSION 

The development of MLs is undoubtedly a success in terms of the commercialization of chemicals derived from cultures of soil micro-organisms, and their subsequent widespread adoption over the past thirty years for the control of agricultural and livestock pests (particularly those of cattle, sheep and horses, but also camels and reindeers) and in human medicine to control onchocerciasis and insect vectors of various diseases. MLs are used on most continents, from theArctic Circle to tropical regions, with environmental consequences on non–target organisms that are still difficult to assess but surely significant. The results of this review clearly demonstrate that in regard to environmental aspects many macrocyclic lactones are substances of high concern. However, with the exception of IVM and, to a lesser extent, MOX and DOR, the knowledge available from the open literature is still very limited. The immediate challenge therefore is to devise ways of filling the gaps in our knowledge base, focusing in particular on:

- The refinement and expansion of our understanding of pharmacokinetics and toxicology in order to provide a better basis for environmental risk assessment of MLs [325];
- Performance of tests on different levels (laboratory, semi-field and field) following standard test guidelines whenever possible (for coprophilous arthropods see, for example, OECD 2008 and OECD 2009) [52-53];
- Performance of higher-tier studies under realistic (field) conditions, including structural (biodiversity) and functional (dung decomposition) endpoints (see for example Jochmann *et al.*) [326];
- Definition of the goals to be protected when assessing the potential environmental risks of new MLs and / or new formulations;
- Linking ecological knowledge (*e.g.* population dynamics of single species) with ecotoxicological risk assessment, with a long-term aim of modelling the impact of these compounds on ecosystems in the field, particularly at the landscape level.

## Figures and Tables

**Table 1.** Physical and Chemical Properties of Avermectins [Values According to Ref. 22 and 162]

Parameter	Ivermectin	Abamectin	Emamectin Benzoate
Molecular weight	875	873.1	994 – 1 008
Kow	1 651	9 772	100 000
Koc	12 660–15 700	5 300–15 700	3 485 – 24 176
Aqueous solubility	4 mg·L^-1^	7.8 μg·L^-1^	24 – 320 mg·L^-1^
Vapour pressure	< 1.5 x 10^-9^ mm Hg	NA	3 x 10^-8^ mm Hg
Photolysis in water	< 0.5 days	< 0.5 days	0.7 – 35.4 days
Soil half-life; and other experimental conditions	93–240 days* 7–14 days** 91–217 days*** 3 h****	14–56 days	174 days

* In the laboratory, in the dark, ~22°C, in soil/feces mixtures.

** Outdoors, in summer, in soil and soil/feces mixtures.

*** Outdoors, in winter, in soil and soil/feces mixtures

**** Outdoors, thin, dry film on glass, sunlight

NA: Not available.

**Table 2.** Ecotoxicity of Abamectin (ABM) to Aquatic and Terrestrial Organisms (for key to Dosages see Table Footnote)

Source	Reference	Test Organism	Toxicity / response	Conditions
		**VERTEBRATES**		
Wislocki *et al.* (1989)	[120]	*Cyprinodon variegates* (Sheepshead minnow)	LC_50_ (96 h ) = 15 µg kg^-1^	Route of exposure to test organisms: dissolved state
Wislocki *et al.* (1989)	[120]	*Ictalurus punctatus* (Channel catfish)	LC_50_ (96 h ) = 24 µg kg^-1^	Route of exposure: dissolved state
Wislocki *et al.* (1989)	[120]	*Cyprinus* spp.	LC_50_ (96 h ) = 42 µg kg^-1^	Route of exposure: dissolved state
Tišler & Eržen (2006)	[118]	*Danio rerio* (Zebrafish)	Mortality (LC): 96 h LC_10_ = 30.8 µg L^-1^ 96 h LC_50_ = 55.1 µg L^-1^ (46.1–66.7 µg.L^-1^) 96 h LC_90_ = 98.3 µg L^-1^ Swimming ability (EC): 96 h EC_50_ = 21.1 µg L^-1^ 96 h EC_50_ = 49.3 µg L^-1^ (37.3–63.3 µg L^-1^) 96 h EC_90_ = 114.8 µg L^-1^	Acute toxicity; exposure: semi-static, duration 96 h Endpoints: mortality and swimming ability
		TERRESTRIAL INVERTEBRATES		
Ridsdill-Smith (1988)	[157]	*Onthophagus binodis* (dung beetle)	Reduced larval survival and oviposition for 4-8 weeks post-treatment	Injectable (cattle)
Dadour *et al.* (2000)	[160]	*Onthophagus binodis* (dung beetle)	Reduced survival of newly emerged beetles in dung voided 3-6 days post-treatment. Inhibition of egg laying in dung voided 5-6 wks previously	Injectable (cattle); Sub-lethal effects
Houlding *et al.* (1991)	[159]	*Onthophagus binodis* (dung beetle)	Reduced survival of newly emerged beetles in dung voided 3-6 days post-treatment; delayed oocyte development	Injectable (cattle)
Doherty *et al.* (1994)	[66]	*Digitonthophagus gazella* (dung beetle)	Oviposition not reduced; complete mortality of larvae with ≥ 16µg a.i. kg^-1^	Injectable 1% formulation (cattle)
Wardhaugh & Mahon (1991)	[155]	*Onthophagus* spp.; *Euoniticellus fulvus* (dung beetles)	Dung of ABM-treated cattle attracted more dung beetles than dung voided by untreated animals	Injectable (cattle)
Youn *et al.* (2003)	[139]	*Harmonia axyridis* (Asian ladybird beetle)	LC_50_ (eggs) <0.09 mg a.i. L^-1^ LC_50_ (1st instar) <0.09 mg a.i. L^-1^ LC_50_ (2nd instar) <0.09 mg a.i. L^-1^ LC_50_ (3rd instar) <0.09 mg a.i. L^-1^ LC_50_ (4th instar) = 18.40 mg a.i. L^-1^ LC_50_ (pupae) <0.09 mg a.i. L^-1^ LC_50_ (4th instar) = 4.90 mg a.i. L^-1^	Mortality 48 h (mobile stages) eggs and pupae: one week
Ahmad *et al.* (2008)	[144]	*Spodoptera litura* (Noctuidae)	LC_50_ =18.5 - 2342 mg L^-1^	Mortality assessed after 72 h exposure to ABM. Comparison laboratory susceptible population with field populations
Wang *et al.* (2008)	[135]	*Anagrus nilaparvata* (Hymenoptera Mymaridae)	LC_50_ = 8.5 mg a.i. L^-1^	1 h exposure (contact); emulsible concentrate 1% a.i.; final test concentration: 16 mg a.i. L^-1^
Guglielmone *et al.* (1999)	[154]	*Haematobia irritans* (Horn fly)	100% mortality in dung voided 7 days post-treatment	Injectable (cattle) egg-adult development
Clarke & Ridsdill-Smith (1990)	[158]	*Musca vetustissima* (Bush fly)	Reduced survival and enhanced asymmetry of wing veins for flies from dung voided 4 weeks post injection; no effect on survival or asymmetry at weeks 8 to 11	Injectable (cattle) egg-adult development
Ridsdill-Smith (1988)	[157]	*Musca vetustissima* (Bush fly)	0% egg-adult survival in dung voided up to 2 wks post-treatment 98% survival by wk 8	Injectable (cattle)
Wardhaugh & Mahon (1991)	[155]	*Musca vetustissima* (Bush fly)	0% larval survival days 3-25. 6 % at day 35	Injectable (cattle)
Wardhaugh & Mahon (1998)	[156]	*Musca vetustissima* (Bush fly)	Fly survival suppressed for 16-32 days	Injectable (cattle)
Kolar *et al.* (2008)	[146]	*Folsomia candida* (springtail)	LC_50_ (survival) = 67 mg kg^-1^ d.s. EC_50_ (reproduction) = 5.2 mg kg^-1^ d.s. EC_50_ (reproduction) = 13 mg kg^-1^ d.s.	Soil; mortality, reproduction after 28 days of exposure
Kolar *et al.* (2008)	[146]	*Folsomia candida* (springtail)	LC_50_ (survival) = 1.0 mg kg^-1^ d.f. NOEC (reproduction) = 0.8 mg kg^-1^ d.f. EC_50_ (reproduction) = 1.4 mg kg^-1^ d.f.	Concentrations of ABM in sheep faeces; mortality, reproduction after 28 days of exposure
Diao *et al.* (2007)	[145]	*Folsomia candida* (springtail)	NOEC (survival) >2.5 mg kg^-1^ dry weight LOEC (survival) >2.5 mg .kg^-1^ d.w. EC_50_ (survival) >2.5 mg kg^-1^ d.w. EC_50_ (survival) >2.5 mg kg^-1^ d.w. NOEC (reproduction) = 0.25 mg kg^-1^ d.w. LOEC (reproduction) = 0.50 mg kg^-1^ d.w. EC_50_ (reproduction) = 0.19 mg kg^-1^ d.w. EC_50_ (reproduction) = 0.68 mg kg^-1^ d.w.	21 days exposure in soil; mortality, reproduction
Diao *et al.* (2007)	[145]	*Folsomia fimetaria* (springtail)	LOEC (survival) =1.00 mg kg^-1^ d.w. EC_50_ (survival) = 0.48 mg kg^-1^ d.w. EC_50_ (survival) = 0.81 mg kg^-1^ d.w. NOEC (reproduction) <0.25 mg kg^-1^ d.w. LOEC (reproduction) = 0.25 mg kg^-1^ d.w. EC_50_ (reproduction) = 0.05 mg kg^-1^ d.w. EC_50_ (reproduction) = 0.33 mg kg^-1^ d.w.	21 days exposure in soil; mortality, reproduction
Umina *et al.* (2010)	[134]	*Sminthurus viridis* (Collembolan, Lucerne flea)	LD50 = 18.94 mg L^-1^	a.i. = 18 g L^-1^; dilution 300 ml 50 L^-1^ water ; contact 8h; mortality
Lin *et al.* (2009)	[94]	*Tetranychus cinnabarinus* (Acariformes, Tetranychidae)	F0 LC_50_ = 0.02 mg L^-1^ F0 LC_90_ = 0.04 mg L^-1^ F42 LC_50_ = 0.15 mg L^-1^ F42 LC_90_ = 0.39 mg L^-1^	Selection of resistance to ABM in Tetranychus cinnabarinus (generations F0 to F42). Mortality 24h
Umina *et al.* (2010)	[134]	*Halotydeus destructor* (redlegged earth mite)	LD50 = 97 mg L^-1^	a.i. = 18 g L^-1^; dilution 300 ml.50 L^-1^ water; contact 8h; mortality
Umina *et al.* (2010)	[134]	*Penthaleus falcatus* (blue oat mite)	LD50 = 30 mg L^-1^	a.i. = 18 g L^-1^; dilution 300 mL 50 L^-1^ water; contact 8h; mortality
Umina *et al.* (2010)	[134]	*Bryobia sp.* (clover mite)	LD50 = 155 mg L^-1^	a.i. = 18 g L^-1^; dilution 300 mL 50 L^-1^ water; contact 8h; mortality
Lin *et al.* (2009)	[94]	*Tetranychus cinnabarinus* (Acariformes, Tetranychidae)	F0 LC_50_ = 0.02 mg L^-1^ F0 LC_90_ = 0.04 mg L^-1^ F42 LC_50_ = 0.15 mg L^-1^ F42 LC_90_ = 0.39 mg L^-1^	Selection of resistance to ABM in Tetranychus cinnabarinus (generations F0 to F42). Mortality 24h
Kolar *et al.* (2008)	[146]	*Porcellio scaber* (isopod)	LC_50_ (survival) = 69 mg kg^-1^ d.s.	Soil; mortality after 21 days of exposure
Wislocki *et al.* (1989)	[120]	*Eisenia fetida* (compost earthworm)	LC_50_ = 28 mg kg^-1^ d.w.	After 28 days exposure
Jensen *et al.* (2007)	[153]	*Eisenia fetida* (compost earthworm)	EC_50_ = 0.06 mg kg^-1^: Change in biomass. LOEC for cocoon production: 0.25 mg kg^-1^ EC_50_ and EC_50_ approximately 0.16 and 1.03 mg kg^-1^ for cocoon production No cocoons production at concentration 5 mg kg^-1^	E. fetida were exposed to 0, 0.25, 0.5, 1, 1.5, 2.5 and 5 mg kg^-1^ d.s.
Kolar *et al.* (2008)	[146]	*Eisenia andrei* (earthworm)	LC_50_ (survival) = 18 mg kg^-1^ d.s. LOEC (weight loss) = 29 mg kg^-1^ d.s. NOEC (weight loss) = 9.8 mg kg^-1^ d.s.	Soil; mortality, weight loss after 28 days of exposure
Kolar *et al.* (2008)	[146]	*Eisenia andrei* (earthworm)	LC_50_ (survival) >1.4 mg kg^-1^ d.f. no effect on reproduction NOEC > 1.4 mg kg^-1^ d.f.	Sheep faeces; mortality, reproduction after 28 days of exposure
Diao *et al.* (2007)	[145]	*Eisenia andrei* (earthworm)	NOEC (survival) = 5.0 mg kg^-1^ d.s. LOEC (survival) >5.0 mg kg^-1^ soil wt EC_50_ (survival) >5.0 mg kg^-1^ soil wt EC_50_ (survival) >5.0 mg kg^-1^ soil wt NOEC (reproduction) < 0.25 mg kg^-1^ soil wt	Soil; mortality, reproduction after 70 days of exposure
Sun *et al.* (2005)	[206]	*Eisenia fetida* (earthworm)	LC_50_ (7 days) = 24 mg kg_1 (d.w.) LC_50_ (14 days) = 17 mg kg_1 (d.w.)	After 14 days of exposure in artificial OECD soil
Kolar *et al.* (2008)	[146]	*Enchytraeus crypticus* (enchytraeid)	EC_50_ (survival) = 111 mg kg^-1^ d.s. EC_50_ (reproduction) = 4.6 mg kg^-1^ d.s. EC_50_ (reproduction) = 38 mg kg^-1^ d.s.	Soil; mortality, reproduction after 28 days of exposure
Kolar *et al.* (2008)	[146]	*Enchytraeus crypticus* (enchytraeid)	LC_50_ (survival) = 1.1 mg kg^-1^ d.f. EC_50_ (reproduction) = 0.9 mg kg^-1^ d.f. NOEC = 0.8 mg kg^-1^ d.f.	Concentrations of ABM in sheep faeces; mortality, reproduction after 28 days of exposure
Diao *et al.* (2007)	[145]	*Enchytraeus crypticus* (enchytraeid)	NOEC (survival) = 10 mg kg^-1^ d.s. LOEC (survival) = 150 mg kg^-1^ d.s. EC_50_ (survival) = 78 mg kg^-1^ d.s. NOEC (reproduction) = 10 mg kg^-1^ d.s. LOEC (reproduction) = 25 mg kg^-1^ d.s. EC_50_ (reproduction) = 12 mg kg^-1^ d.s. EC_50_ (reproduction) = 24 mg kg^-1^ d.s.	21 days exposure in soil; mortality, reproduction
			Aquatic Invertebrates	
Wislocki *et al.* (1989)	[120]	*Penaeus duorarum* (Decapoda)	96 h LC_50_ = 1.6 µg kg^-1^	Route of exposure: dissolved state; 96 h exposure
Wislocki *et al.* (1989)	[120]	*Callinectes sapidus* (Blue crab, Decapoda)	96 h LC_50_ = 153 µg kg^-1^	Route of exposure: dissolved state; 96 h exposure
Wislocki *et al.* (1989)	[120]	*Daphnia magna* (Cladocera)	48 h LC_50_ = 0.34 µg kg^-1^	Route of exposure: dissolved state; 24 h exposure
Tišler & Eržen (2006)	[118]	*Daphnia magna* (Cladocera)	24 h EC_50_ = 0.11 µg L^-1^ 48 h EC_50_ = 0.12 µg L^-1^ 24 h EC_50_ = 0.33 µg L^-1^ (range 0.21–0.43 µg.L^-1^) 48 h EC_50_ = 0.25 µg L^-1^ (range 0.21–0.30 µg.L^-1^) 24 h EC_90_ = 0.97 µg L^-1^ 48 h EC_90_ = 0.50 µg L^-1^	Acute toxicity. Exposure: static; duration 24 and 48 h Endpoint: immobility
Tišler & Eržen (2006)	[118]	*Daphnia magna* (Cladocera)	21 d LOEC = 0.009 µg L^-1^ 21 d NOEC = 0.005 µg L^-1^ 21 d IC25 = 0.007 µg L^-1^	Chronic toxicity.
Wislocki *et al.* (1989)	[120]	*Neomysis bahia* (Mysidacea)	96 h LC_50_ = 0.022 µg kg^-1^	Route of exposure: dissolved state; 96 h exposure
Wislocki *et al.* (1989)	[120]	*Crassostrea virginica* (Bivalvia)	96 h LC_50_ = 430 µg kg^-1^	Route of exposure to test organisms: dissolved state; 96 h exposure
			ALGAE	
Tišler & Eržen (2006)	[118]	*Scenedesmus subspicatus*	no growth inhibition at 10 µg L^-1^ ABM 72 h EC_50_ = 0.7 mg L^-1^ 72 h EC_50_ = 4.4 mg L^-1^ 72 h EC_90_ = 21 mg L^-1^	Exposure: static; chronic toxicity
			MICRO-ORGANISMS	
Tišler & Eržen (2006)	[118]	*Vibrio fischeri* (luminescent bacteria)	30 min EC20 = 390 µg L^-1^ 30 min EC_50_ = 690 µg L^-1^ (610-770 µg L^-1^) 30 min EC_80_ = 1200 µg L^-1^	Exposition (static) 30 min to ABM; endpoint: luminescence

Dosages for cattle are: 500 (pour-on), 200 (injectable) or 200 (oral) µg kg^-1^ b.w.

**Table 3.** Ecotoxicity of Ivermectin (IVM) to Aquatic and Terrestrial Organisms (for key to Dosages see Table Footnote)

Source	Reference	Test organism	Toxicity / response	Conditions
		VERTEBRATES		
Geets *et al.* (1992)	[327]	*Anguilla anguilla* (eel)	24 h LC_50_ = 0.2 mg kg^-1^	Route of exposure to test organisms: dissolved state
Halley *et al.* (1989)	[70]	*Lepomis macrochirus* (Bluegill sunfish)	96 h LC_50_ = 4.8 mg L^-1^	Route of exposure: dissolved state
Halley *et al.* (1989)	[70]	*Salmo gardneri* (Rainbow trout)	96 h LC_50_ = 3.0 mg L^-1^ 96 h NOEC = 0.9 mg L^-1^	Route of exposure: dissolved state
Kilmartin *et al.* (1997)	[223]	*Salmo gardneri* (Rainbow trout)	96 h LC_50_ = 3 µg kg^-1^	Route of exposure: dissolved state
Kilmartin *et al.* (1997)	[223]	*Salmo salar* (Atlantic salmon)	96 h LC_50_ = 500 µg kg^-1^	Route of exposure: injection
Halley *et al.* (1989)	[70]	*Salmo salar* (Atlantic salmon)	96 h LD50 = 17 mg kg^-1^	Route of exposure to test organisms: dissolved state
Wislocki *et al.* (1989)	[120]	*Salmo trutta* (Brown trutta)	96 h LC_50_ = 300 µg kg^-1^	Route of exposure: injection
Katharios *et al.* (2001)	[328]	*Sparus aurata* (Sea bream)	35 d LC_50_ = 0% mortality	Peritoneal injection of doses of 100 – 800 µg kg^-1^ fish
		TERRESTRIAL INVERTEBRATES		
Wardhaugh & Rodriguez-Menendez (1988)	[172]	*Copris hispanus* (dung beetle)	Mortality of newly emerged adults fed on dung voided 2-16 days after injection. Mature adults unaffected	Injectable (calves); dose rate of 0.2 mg.kg^-1^ of body weight
Iwasa *et al.* (2007)	[329]	*Caccobius jessoensis, Copris ochus, Copris acutidens*	Altered oviposition by adults in dung ≤ 7 days post-treatment; reduced survival of offspring in dung 3 days post-treatment	Pour-on (cattle)
Fincher (1996)	[330]	*Euoniticellus intermedius* (dung beetle)	Reduced egg-adult survival in dung voided 1-2 weeks post-treatment; no effect on brood ball production	Pour-on (cattle)
Fincher (1996)	[330]	*Digitonthophagus gazella* (dung beetle)	Reduced egg-adult survival in dung voided 2-3 weeks post-treatment; no effect on brood ball production	Pour-on (cattle)
Wardhaugh & Mahon (1991)	[155]	*Onthophagus* spp.; *Euoniticellus fulvus* (dung beetles)	Dung of IVM-treated sheep attracted more dung beetles than dung voided by untreated animals	Oral drench (sheep)
Wardhaugh *et al.* (1993)	[100]	*Euoniticellus fulvus* (dung beetle)	Larval mortality confined to dung voided 1-2 days post-treatment. Mortality and delayed maturation in newly emerged beetles	Oral drench (sheep)
Wardhaugh *et al.* (2001)	[180]	*Onthophagus sagittarius* (dung beetle)	Inhibited larval survival for 15 weeks post-treatment	IVM SR bolus formulation: 1.72g of IVM (cattle)
Wardhaugh *et al.* (2001)	[179]	*Onthophagus taurus*; *Euoniticellus fulvus* (dung beetles)	Excreted residues inhibited larval survival. High mortality and delayed maturation in newly emerged beetles	Controlled release capsule containing. 160 mg of IVM (sheep)
Sommer *et al.* (1993)	[189]	*Diastellopalpus quinquedens* (dung beetle)	Reduced % of brood masses with live larvae and reduced larvae or pupae on day 2	Injectable (cattle) 28 days; development, mortality, morphology of head capsule.
Lumaret *et al.* (1993)	[173]	*Euoniticellus fulvus* (dung beetle)	Slight delay in development. Increased number of beetles in treated pats	Injectable (cattle); 0.20 mg IVM kg^-1^ b.w. 30 days
Krüger & Scholtz (1997)	[188]	*Euoniticellus intermedius* (dung beetle)	Reduced number of brood balls on day 3; reduced emergence days 2 to 14; 0 to 3% survival days 2 to 14; development time prolonged days 1 to 28; adult fertility reduced day 1.	Injectable (cattle) 56 days; emergence, development, survival, fecundity and fertility
Iwasa *et al.* (2005)	[331]	*Liatongus minutus* (dung beetle)	Reduced egg-adult survival in dung voided ≤ 14 days post-treatment; possibly delayed development	Pour-on (cattle)
Krüger & Scholtz (1997)	[188]	*Onitis alexis* (dung beetle)	Reduced emergence days 2 to 7; prolonged development days 1, 2, 4 to 21; no difference in adult live mass	Injectable (steers) 56 days; emergence, development, adult size
Sommer *et al.* (1993)	[189]	*Digitonthophagus gazella* (dung beetle)	Reductions in development and mortality on days 2 and 8; reduced head capsule width	Injectable (cattle); 56 days; development, mortality, morphology of head capsule
Sommer & Nielsen (1992)	[190]	*Digitonthophagus gazella* (dung beetle)	17 days	Injectable (cattle) Sensitivity of coleopteran larvae, indicated by days post-treatment until adult emergence from dung equalled that of control
Fincher (1992)	[191]	*Digitonthophagus gazella* (dung beetle)	21 days	Injectable (steers) Sensitivity of coleopteran larvae, indicated by days post-treatment until adult emergence from dung equalled that of control
Dadour *et al.* (1999)	[192]	*Onthophagus taurus* (dung beetle)	Reductions on days 7 and 10 post-treatment	Injectable (cattle) 15 days; % dung pat dispersal, number of beetles / pat
Strong *et al.* (1996)	[186]	Scarabaeidae larvae	Residues reduced oviposition and inhibited larval development but had no effect on numbers of adult beetles.	IVM SR bolus formulation: 1.72 g of IVM (cattle)
Errouissi *et al.* (2001)	[185]	*Aphodius constans* (dung beetle)	No development until day 105 post-treatment; reduction emergence until day 135.	SR bolus IVM; dose rate of 12 mg day–1 over 135 days. Larval development.
Lumaret *et al.* (2007)	[195]	*Aphodius constans* (dung beetle)	LC_50_ = 590 µg kg^-1^ dung (f.w.)	Pour-on (cattle) Larval mortality during the first 3 weeks post- treatment
Hempel *et al.* (2006)	[193]	*Aphodius constans* (dung beetle)	LC_50_ = 0.88 – 0.98 mg kg^-1^ dung (d.w.)	Spiked dung
Finnegan *et al.* (1997)	[332]	*Aphodius sphacelatus* (dung beetle)	Reduced activity of adult beetles in dung increases sporulation by the coprophilous fungus Pilobius	Pour-on (cattle) or spiked dung (1 mg kg^-1^ IVM dung wet wt.); pour-on (cattle)
Madsen *et al.* (1990)	[177]	*Aphodius* spp (dung beetle)	10 days	Injectable (cattle) Sensitivity of coleopteran larvae, indicated by days post-treatment until adult emergence from dung equalled that of control
Sommer *et al.* (1992)	[175]	*Aphodius* spp. (dung beetle)	13-14 days	Injectable (cattle) Sensitivity of larvae, indicated by days post-treatment until adult emergence from dung equalled that of control
Strong & Wall (1994)	[263]	*Aphodius* spp. (dung beetle)	14 days	Injectable (cattle) Sensitivity of larvae, indicated by days post-treatment until adult emergence from dung equalled that of control
Webb *et al.* (2010)	[333]	*Aphodius* spp. (dung beetles)	Increased capture of adults in pastures with avermectin-treated versus untreated cattle	Pour-on (cattle)
Holter *et al.* (1993)	[334]	Coleoptera (several species individually)	Altered attraction to dung of treated animals	Injectable (cattle)
Floate (1998)	[335]	Coleoptera (several species individually)	Altered attraction to dung voided 1 and 4 weeks post-treatment	Pour-on (cattle)
Floate (1998)	[336]	Coleoptera (several species individually)	Altered attraction to dung voided 1 and 4 weeks post-treatment	Pour-on (cattle)
Barth *et al.* (1993)	[187]	Coleoptera (several species combined)	Suppression of larvae, but not adults, in dung deposited 21, 70 and 199 days post-treatment	SR bolus formulation with 12 mg day–1 over 120 days (cattle)
Kryger *et al.* (2005)	[337]	Coleoptera (several species)	No effect on species richness or diversity	Injectable (cattle)
Fincher (1992)	[191]	*Haematobia irritans* (horn fly)	Effects 63 days	Injectable (steers)
Schmidt (1983)	[338]	*Haematobia irritans* (horn fly)	Efects 42 days	Injectable (cattle)
Fincher (1996)	[330]	*Haematobia irritans* (horn fly)	Reduced egg-adult survival in dung voided 5-6 weeks post-treatment	Pour-on (cattle)
Römbke *et al.* (2010)	[182]	*Musca autumnalis* (face fly)	EC_50_ (emergence) = 4.65 ± 2.17 (SD) µg IVM kg^-1^ fresh dung (range: 1.2 – 7.7); NOEC = between 1.1 and 3.3 µg IVM kg^-1^ dung f.w. No effect on development time.	Spiked cattle dung with 5 concentrations between 0.37 and 30 µg·kg^-1^ dung f.w. (cattle)
Wardhaugh & Mahon (1991)	[156]	*Musca vetustissima* (bush fly)	IVM - 0% larval survival days 1-6; 100% survival at day 28	Oral drench (sheep)
Wardhaugh *et al.* (1993)	[100]	*Musca vetustissima* (bush fly)	0% survival days 1 to 4; No evidence of fluctuating asymmetry	Oral drench (sheep)
Wardhaugh *et al.* (1996)	[174]	*Musca vetustissima* (bush fly)	IVM inhibited larval growth for 7 to 14 days after treatment.	Injectable (steers)
Wardhaugh & Mahon (1998)	[156]	*Musca vetustissima* (bush fly)	Reduced larval survival for 16 days in both assays	Oral drench (cattle) Injectable (cattle)
Wardhaugh *et al.* (2001)	[179]	*Musca vetustissima* (bush fly)	No larvae survived in faeces voided up to 39 days post-application	Controlled release capsule containing 160mg of IVM (sheep)
Cook (1991)	[171]	*Lucilia cuprina* (sheep blowfly)	Cessation of oocyte development, increased mortality	Oral drench.(sheep); ad lib feeding of adults on dung of sheep 18 days post-treatment
Cook (1993)	[170]	*Lucilia cuprina* (sheep blowfly)	Fewer mating attempts until 6 days, longer mating duration, no difference in % mating; delayed oviposition; increased mortality	Oral drench (sheep); Mortality, mating and reproduction
Mahon *et al.* (1993)	[169]	*Lucilia cuprina* (sheep blowfly)	Reduced adult survival, delayed reproductive development and reduced fecundity in dung voided 1 day after treatment. Increased mortality; fewer gravid females, and reduced oocyte production day 1; reduction in mature oocyte retention, no effect on egg viability	Oral drench (sheep); 14 days and 6 days exposure to residues; mortality, fecundity and ovarian development
Wardhaugh *et al.* (2001)	[180]	*Chrysomya bezziana* (Old World screw-worm fly (OWS))	IVM gave 14-102 days protection	IVM SR bolus formulation 1.72g of IVM
Wardhaugh *et al.* (1996)	[174]	*Musca domestica* (house fly)	IVM inhibited larval growth for 7 to 14 days after treatment.	Injectable (steers)
Floate *et al.* (2001)	[178]	*Musca domestica* (house fly)	no emergence at wk 1; < control at wk 2	Pour-on (cattle) Survival of flies developing in dung
Farkas *et al.* (2003)	[264]	*Musca domestica* (house fly)	Reduced larva-to-adult survival in dung voided ≤ 28 (cattle) or ≤ 11 (swine) days post-treatment	Injectable (cattle) Injectable (swine) 300 µg·kg^-1^ b.w.
Floate & Fox (1999)	[339]	*Musca domestica* (house fly)	Pupae exposed to ≥ 0.25 mg kg^-1^ IVM produced 63% fewer parasitoids than control pupae. Pupae exposed to 0.01 ppm IVM produced 23% more parasitoids. No detectable effect exposure to 0.10 mg kg^-1^ IVM	Fly-rearing media spiked with IVM at concentrations ranging from 0.01 to 1.50 mg kg^-1^
Madsen *et al.* (1990)	[177]	*Musca domestica* (house fly)	Increased mortality for 20 days	Injectable (cattle); 30 days; mortality
Marley *et al.* (1993)	[340]	*Musca domestica* (house fly)	Reduced egg-adult survival in dung voided ≤ 11 days post-treatment.	Pour-on (cattle)
Mayer *et al.* (1980)	[176]	*Musca autumnalis* (face fly)	14 days	Injectable (cattle) Sensitivity of dipteran larvae, indicated by days post-treatment until adult emergence from dung equalled that of control
Krüger & Scholtz (1995)	[168]	*Musca nevilli* (dung-breeding fly)	Development delayed 4 weeks; 0% larval survival and emergence 4 weeks; reduced fertility 60%	Injectable (cattle); 0.20 mg IVM kg^-1^ b.w. 56 days; development, survival, emergence, reproduction
Iwasa *et al.* (2005)	[331]	*Musca bezzii* (fly)	Reduced pupation in dung voided ≤ 21 days post-treatment	Pour-on (cattle)
Miller *et al.* (1981)	[341]	*Haematobia irritans* (horn fly)	56 days	Sensitivity of dipteran larvae, indicated by days post-treatment until adult emergence from dung equalled that of control.
Floate *et al.* (2001)	[178]	*Haematobia irritans* (horn fly)	No emergence 4 weeks after treatment.	Pour-on (cattle) Survival of flies developing in dung.
Schmidt (1983)	[338]	*Stomoxys calcitrans* (stable fly)	14 days	Sensitivity of dipteran larvae, indicated by days post-treatment until adult emergence from dung equalled that of control
Floate *et al.* (2001)	[178]	*Stomoxys calcitrans* (stable fly)	< control at weeks 1 and 4	Pour-on (cattle); survival of flies developing in dung.
Lumaret *et al.* (1993)	[173]	*Neomyia cornicina* (dung-dwelling Diptera)	Significant decrease until day 23; no larval development at 0.16 mg kg^-1^ dung	Injectable (steers). Sensitivity of dipteran larvae, indicated by days post-treatment until adult emergence from dung equalled that of control
Wardhaugh *et al.* (2001)	[180]	*Musca inferior* (livestock fly)	Inhibited larval survival for >16 days	IVM SR bolus formulation 1.72g of IVM
Boxall *et al.* (2002)	[167]	*Neomyia cornicina* (dung fly)	0.13 mg kg^-1^	behaviour
Boxall *et al.* (2002)	[167]	*Neomyia cornicina* (dung fly)	0.13 mg kg^-1^	47% mortality over 7 d (dung)
Boxall *et al.* (2002)	[167]	*Neomyia cornicina* (dung fly)	0.25 mg kg^-1^	77% mortality over 7 d (dung)
Boxall *et al.* (2002)	[167]	*Neomyia cornicina* (dung fly)	0.50 mg kg^-1^	87% mortality over 7 d (dung)
Boxall *et al.* (2002)	[167]	*Neomyia cornicina* (dung fly)	1 mg kg^-1^	100% mortality over 7 d (dung)
Boxall *et al.* (2002)	[167]	*Neomyia cornicina* (dung fly)	0.14 mg kg^-1^	7 d LC_50_
Gover & Strong (1995)	[342]	*Neomyia cornicina* (dung fly)	LC_50_ = 0.139 and LC95 = 0.393 mg kg^-1^ (f.w.); reduced and delayed oviposition; reduced egg hatch	Spiked dung; adult feeding for 1 week
Gover & Strong (1995)	[343]	*Neomyia cornicina* (dung fly)	Reduced excretion and increased abdominal mass	Spiked dung; adult feeding for 3-5 days
Wardhaugh & Rodriguez-Menendez (1988)	[172]	*Orthelia cornicina* (dung fly)	Larval mortality exceeded 97% in dung voided 1-32 days post-injection	Injectable (calves); dose rate of 0.2 mg kg^-1^ of body weight 30 days
Wardhaugh *et al.* (2001)	[180]	*Orthelia timorensis *	Inhibited larval survival for >16 days	IVM SR bolus formulation 1.72g of IVM
Strong & James (1993)	[98]	*Scatophaga stercoraria*(yellow dung fly)	EC_50_ = 0.05 mg kg^-1^ (d.w.)	24h mortality (larvae)
Strong & James (1993)	[98]	*Scatophaga stercoraria*(yellow dung fly)	EC_50_ = 0.04 mg kg^-1^ (d.w.)	48h mortality (larvae)
Strong & James (1993)	[98]	*Scatophaga stercoraria* (yellow dung fly)	EC_50_ = 0.001 mg kg^-1^	10 days emergence; 50% reduction in emergence
Strong & James (1993)	[98]	*Scatophaga stercoraria *(yellow dung fly)	EC_50_ = 0.02 mg kg^-1^	10 days pupariate ; 50% reduction in pupation
Strong & James (1993)	[98]	*Scatophaga stercoraria* (yellow dung fly)	0.0005 mg kg^-1^	10 days fluctuating asymmetry
Iwasa *et al.* (2005)	[331]	*Scatophaga stercoraria* (yellow dung fly)	Reduced egg-adult survival in dung voided ≤ 28 days post-treatment	Pour-on (cattle)
Webb *et al.* (2007)	[333]	*Scatophaga stercoraria *(yellow dung fly)	No difference in recovery of adult flies from pastures with treated versus untreated cattle	Pour-on (cattle)
Römbke *et al.* (2009)	[344]	*Scatophaga stercoraria* (yellow dung fly)	Egg-to-adult mortality: EC_50_ = 20.9 ug kg^_1^ dung f.w.; Mortality: NOEC = 8.1 ug kg^_1^ f.w.; Prolonged development: NOEC < 0.8 ug kg^_1^ f.w.	Spiked dung seeded with eggs
West & Tracy (2009)	[345]	*Scatophaga stercoraria* (yellow dung fly)	Elevated phenoloxidase activity (i.e., possibly elevated immune function) in adults reared with exposure to residues of 0.001 mg kg^_1^ d.w. (= 0.0002 mg kg^-1^ f.w.)	Spiked dung seeded with eggs.
Floate & Coghlin (2010)	[101]	*Scatophaga stercoraria *(yellow dung fly)	No effect of residues on fluctuating asymmetry of wing or wing traits for adults reared with exposure to residues	Spiked dung seeded with eggs
Strong *et al.* (1996)	[186]	Diptera Cyclorrhapha	Larval development completely inhibited	IVM SR bolus formulation: 1.72g IVM (cattle)
Madsen *et al.* (1990)	[177]	Diptera Cyclorrapha	> 30 days	Injectable (cattle) Sensitivity of dipteran larvae, indicated by days post-treatment until adult emergence from dung equalled that of control
Sommer *et al.* (1992)	[175]	Diptera Cyclorrapha	42 days	ditto
Madsen *et al.* (1990)	[177]	Diptera Nematocera	>10 days	ditto
Sommer *et al.* (1992)	[175]	Diptera Nematocera	0 day	ditto
Jensen *et al.* (2003)	[207]	*Folsomia fimetaria *(springtail)	28 d NOECreprod = 0.3 mg kg^_1^ soil (d.w.) 28 d LC_50_ = 8.4 mg kg^_1^ soil (d.w.) 28 d EC_50_ reproduction = 0.26 mg kg^_1^ soil (d.w.) 28 d ECreprod = 1.7 mg kg^_1^ soil (d.w.)	field soil: TOC 1.6% mortality, reproduction, 28 days
Barth *et al.* (1993)	[346]	Diptera (several species combined)	Suppression of larvae in dung deposited 21, 70 and 199 days post-treatment	SR bolus formulation with 12 mg day_1 over 120 days.
Floate & Fox (1999)	[339]	*Muscidifurax zaraptor* (Pteromalidae)	Fewer wasps from fly pupae reared with exposure to ≥ 0.25 mg kg^-1^ IVM; more wasps from fly pupae reared with exposure to 0.01 mg kg^-1^ IVM	Development in the puparia of house flies reared in media spiked with IVM at concentrations ranging from 0.01 to 1.50 ppm
Jensen *et al.* (2009)	[208]	*Folsomia fimetaria *(springtail)	28 d NOEC_reprod._=0.4 mg kg^_1^ soil (d.w.) 28 d EC_50_ reprod. = 0.9 mg kg^_1^ soil (d.w.) 28 d LC_50_ = 5.3 mg kg^_1^ soil (d.w.)	field soil: TOC 2.2%
Jensen *et al.* (2009)	[208]	*Folsomia fimetaria *(springtail)	21 d NOEC_reprod._ < 0.2 mg kg^_1^ soil (d.w.) 21 d EC50reprod. = 0.11 mg kg^_1^ soil (d.w.) 21 d LC_50_ = 0.14 mg kg^_1^ soil (d.w.)	21 days, reproduction, with H. aculeifer (field soil: TOC 2.2%)
Römbke *et al.* (2010)	[205]	*Folsomia candida *(springtail)	28 d NOEC_reprod._ = 0.3 mg kg^_1^ soil (d.w.) 28 d EC50reprod. = 1.7 mg kg^_1^ soil (d.w.) 28 d LC_50_ = 12.4 mg kg^_1^ soil (d.w.)	artificial soil: TOC 2.7%
Jensen *et al.* (2009)	[208]	*Hypoaspis aculeifer *(predatory mite)	21 d NOEC_reprod._ = 5 mg kg^_1^ soil (d.w.) 21 d EC_50_ reprod. = 5 mg kg^_1^ soil (d.w.) 21 d LC_50_ = 5 mg kg^_1^ soil (d.w.)	21 days, reproduction, with F. fimetaria (field soil: TOC 2.2%)
Römbke *et al.* (2010)	[205]	*Hypoaspis aculeifer *(predatory mite)	16 d NOEC_reprod._ = 3.2 mg kg^_1^ soil (d.w.) 16 d EC_50_ reprod. = 17.8 mg kg^_1^ soil (d.w.) 16 d LC_50_ = 31.6 mg kg^_1^ soil (d .w.)	artificial soil: TOC 2.7%
Kolar *et al.* (2008)	[146]	*Eisenia fetida* (earthworm)	no survival at soil concentrations > 20 mg kg^_1^ soil (d.w.) LC_50_ = 15.8 mg kg^-1^	14 days mortality
Gunn & Sadd (1994)	[200]	*Eisenia fetida* (earthworm)	EC_50_ = 4.7 mg IVM kg^-1^ 14 d LC/EC_50_ = 15.7 mg kg^-1^ soil	14 days growth artificial soil
Gunn & Sadd (1994)	[200]	*Eisenia foetida *(earthworm)	EC_50_ = 4.0 ppm 14 d NOEC biomass = 4.0 mg kg^_1^ (d.w.)	14 days cocoon production artificial soil
Gunn & Sadd (1994)	[200]	*Eisenia fetida* (earthworm)	56% fewer cocoon over 21 days at a soil concentration of 4 mg kg^-1^	21 days cocoon production artificial soil
Halley *et al.* (1989)	[70]	*Eisenia fetida* (earthworm)	28 d LC_50_ = 314 mg kg^_1^ soil (d.w.) 28 d NOEC biomass = 12 mg kg^_1^ soil (d.w.)	28 days mortality Chronic earthworm toxicity test (artificial soil)
Römbke *et al.* (2010)	[205]	*Eisenia fetida* (earthworm)	28 d NOEC biomass = 5.0 mg kg^_1^ soil (d.w.) 56 d NOEC reprod. = 2.5 mg kg^_1^ soil (d.w.) 56 d EC_50_ reprod. = 5.3 mg kg^_1^ soil (d.w.) 28 d LC_50_ ≥ 10 mg kg^_1^ soil (d.w.)	artificial soil, TOC 2.7%
Svendsen *et al.* (2002)	[199]	*Lumbricus terrestris *(earthworm)	Mean growth rate higher than in control	Pastures with treated heifers; SR IVM bolus 24 weeks survival and growth
Kaneda *et al.* (2006)	[201]	*Megascolecidae* (earthworm)	No effect on numbers or dung degradation	Spiked dung with 0, 0.1, and 1 mg IVM.kg^_1^ dung (w.w.)
Jensen *et al.* (2003)	[207]	*Enchytraeus crypticus *(Enchytraeidae)	28 d NOECreprod = 3 mg kg^-1^ soil (d.w.) 28 d EC_50_ = 14 mg kg^_1^ soil (d.w.) 28 d EC_50_ reprod = 36 mg kg^_1^ soil (d.w.) 28 d LC_50_ > 300 mg kg^_1^ soil (d.w.)	Field soil: TOC 1.6%
Grønvold *et al.* (2004)	[212]	*Pristionchus maupasi* (Nematode)	No survival at concentration of 5 mg kg^-1^ faeces (w.w.)	IVM picked in cattle dung; 0.5 to 40 µg IVM g^-1^ faeces (w.w.)
		AQUATIC INVERTEBRATES		
Egeler *et al.* (2010)	[218]	*Chironomus riparius *(Diptera larvae)	10 d NOEC, larval growth = 3.1 µg kg^-1^ dry wt	Test method: OECD 218 (2004), sediment exposure
Garric *et al.* (2007)	[215]	*Daphnia magna* (Cladocera)	48 h EC_50_ (immobilisation) = 5.7 ng L^-1^ (range 1.2 to 10.7 ng IVM L^-1^) 21 d LOEC (growth rate) = 0.001 ng L^-1^ 21 d NOEC (growth rate) = 0.0003 ng L^-1^ 21 d LOEC (reproduction) = 0.001 ng L^-1^ 21 d NOEC (reproduction) = 0.0003 ng L^-1^ 21 d LOEC (sex ratio) = 0.001 ng L^-1^ 21 d NOEC (sex ratio) = 0.0003 ng L^-1^	Static 48 h-acute test (immobilisation). Semi-static 21 d-reproduction test (growth rate, reproduction, sex-ratio)
Halley *et al.* (1989)	[70]	*Daphnia magna *(Cladocera)	48 h LC_50_ = 0.025 µg kg^-1^ 48 h NOEC = 0.01 µg kg^-1^ Acute toxicity 25 µg L^-1^	Route of exposure: dissolved state
Halley *et al.* (1989)	[70]	*Daphnia magna *(Cladocera)	48 h LC_50_ = 0.4 µg kg^-1^ 48 h NOEC = 0.1 µg kg^-1^	Route of exposure: dissolved state
Halley *et al.* (1989)	[70]	*Daphnia magna *(Cladocera)	48 h LC_50_ > 17 µg kg^-1^ 48 h NOEC > 9 µg kg^-1^	Route of exposure: dissolved state
Halley *et al.* (1993)	[347]	*Daphnia magna *(Cladocera)	48 h CE50 = 39 µg kg^-1^	Route of exposure: sediment
Halley *et al.* (1993)	[347]	*Daphnia magna* (Cladocera)	48 h LC_50_ = 6.5 µg kg^-1^	Route of exposure: dissolved state; dung leachate
Nessel *et al.* (1989)	[74]	*Daphnia magna *(Cladocera)	to some extent toxic	
Schweitzer *et al.* (2010)	[216]	*Daphnia magna *(Cladocera)	NOEC = 53 µg kg^-1^ dung dry weight	Water–sediment test system; addition of IVM-spiked cattle dung; mortality
Grant & Briggs (1998)	[221]	*Artemia salina* (Anostraca)	24 h LC_50_ > 300 µg L^-1^	Route of exposure: dissolved state
Grant & Briggs (1998)	[221]	*Sphaeroma rugicauda* (Isopoda)	96 h LC_50_ = 348 µg kg^-1^	Route of exposure: dissolved state
Burridge & Haya (1993)	[222]	*Crangon septemspinosa *(Decapoda)	24 h LC_50_ = 13.1 mg kg^-1^ 48 h LC_50_ = 9.7 mg kg^-1^ 96 h LC_50_ > 21.5 µg kg^-1^	Route of exposure: food Route of exposure: food Route of exposure: dissolved state
Grant & Briggs (1998)	[221]	*Palaemonetes varians *(Decapoda)	96 h LC_50_ = 54 µg L^-1^	Route of exposure: dissolved state
Grant & Briggs (1998)	[221]	*Gammarus duebeni *(Amphipoda)	96 h LC_50_ = 0.033 µg L^-1^	Route of exposure: dissolved state
Grant & Briggs (1998)	[221]	*Gammarus zaddachi *(Amphipoda)	96 h LC_50_ = 0.033 µg L^-1^	Route of exposure: dissolved state
Grant & Briggs (1998)	[221]	*Carcinus maenas *(Decapoda)	96 h LC_50_ = 957 µg L^-1^	Route of exposure: dissolved state
Davies *et al.* (1998)	[225]	*Corophium volutator* (Amphipoda)	10 d LC_50_ = 0.18 mg L^-1^	Route of exposure: sediment
Grant & Briggs (1998)	[221]	*Neomysis integer *(Mysidacea)	48 h LC_50_ = 0.026 µg L^-1^	Route of exposure: dissolved state
Davies *et al.* (1998)	[225]	*Neomysis integer* (Mysidacea)	96 h LC_50_ = 0.07 µg L^-1^	Route of exposure: dissolved state
Davies *et al.* (1998)	[225]	*Asterias rubens *(Asteroida)	10 d LC_50_ = 23.6 mg kg^-1^	Route of exposure: sediment
Grant & Briggs (1998)	[221]	*Arenicola marina *(lugworm, Polychaeta)	10 d LC_50_ = 23.0 µg kg^-1^	Route of exposure: dry sediment
Thain *et al.* (1997)	[226]	*Arenicola marina* (lugworm, Polychaeta)	10 d LC_50_ = 0.018 mg a.i. kg^-1^ wet sediment (= 0.023 mg kg^-1^ d.w.) 10 d LOEC = 0.019 mg a.i. kg^-1^ wet sediment (= 0.024 mg kg^-1^ d.w) 10 d NOEC = 0.012 mg a.i. kg^-1^ wet sediment (= 0.015 mg kg^-1^ d.w.).	Route of exposure: sediment. 10 days exposure
Grant & Briggs (1998)	[221]	*Nereis diversicolor* (Polychaeta)	96 h LC_50_ = 0.0075 mg.L^-1^	Route of exposure: dissolved state
Boxall *et al.* (2002), Thain *et al.* (1997)	[167, 226]	*Arenicola marina *(Polychaeta)	<0.005 mg kg^-1^	Effects on feeding
Boxall *et al.* (2002), Thain *et al.* (1997)	[167, 226]	*Arenicola marina *(Polychaeta)	>0.008 mg kg^-1^	Effect on burrowing
Ding *et al.* (2001)	[217]	*Lumbriculus variegates *(freshwater oligochaete)	72 h LC_50_ ~490 µg L^-1^	Water
Egeler *et al.* (2010)	[218]	*Lumbriculus variegates *(ben thic oligochaete)	56 d NOEC = 160 µg kg^-1^ d.w. 28 d NOEC, reprod., biomass = 160 µg kg^-1^ d.w.	Sediment exposition, OECD 225 (2007)
Liebig *et al.* (2010)	[58]	*Caenorhabditis elegans* (nematode)	96 h NOEC, reprod. ≤ 1.0 µg L^-1^	Test method: ISO/CD 10872 (2008) (water-only exposure)
Liebig *et al.* (2010)	[58]	*Caenorhabditis elegans* (nematode)	96 h NOEC, reprod. = 100 µg kg^-1^ dry wt	Test method: ISO/CD 10872 (2008) (sediment exposure)
Kilmartin *et al.* (1997)	[223]	*Crassostrea gigas *(Bivalvia)	96 h LC_50_ (larvae) = 80-100 µg L^-1^ 96 h LC_50_ (spat) = 600 µg L^-1^	Route of exposure: dissolved state
Kilmartin *et al.* (1997)	[223]	*Mytilus edulis* (Bivalvia)	96 h LC_50_ = 400 µg L^-1^	Route of exposure: dissolved state
Kilmartin *et al.* (1997)	[223]	*Pecten maximus* (Bivalvia)	96 h LC_50_ = 300 µg L^-1^	Route of exposure: dissolved state
Kilmartin *et al.* (1997)	[223]	*Tapes semidecussata *(Bivalvia) Larvae Spat	96 h LC_50_ = 380 µg L^-1^ 96 h LC_50_ = 460 µg L^-1^	Route of exposure: dissolved state
Boxall et al. (2002), Davies & Rodger (2000)	[167, 220]	*Monodonta lineata* (Gasteropoda)	96 h LC_50_ = 0.78 mg L^-1^	Route of exposure: dissolved state
Matha & Weiser (1988)	[224]	*Biomphlaria glabrata *(Gasteropoda)	24 h LC_50_ = 0.03 mg L^-1^	Route of exposure: dissolved state
Grant & Briggs (1998)	[221]	*Hydrobia ulvae *(Gasteropoda)	96 h LC_50_ > 10 mg L^-1^	Route of exposure: dissolved state
Grant & Briggs (1998)	[221]	*Potamopyrgus jenkinsii *(Gasteropoda; freshwater snail)	96 h LC_50_ < 9 mg L^-1^	Route of exposure: dissolved state
Grant & Briggs (1998)	[221]	*Littorina littorea* (Gasteropoda)	96 h LC_50_ > 1000 mg L^-1^	Route of exposure: dissolved state
Kilmartin *et al.* (1997)	[223]	*Littorina littorea *(Gasteropoda)	96 h LC_50_ = 580 mg L^-1^	Route of exposure: dissolved state
Kilmartin *et al.* (1997)	[223]	*Nucella lapillus *(Gasteropoda)	96 h LC_50_ = 390 µg L^-1^	Route of exposure: dissolved state
Kilmartin *et al.* (1997)	[223]	*Patella vulgata *(Gasteropoda)	96 h LC_50_ = 600 µg L^-1^	Route of exposure: dissolved state
		Algae and Plants		
Halley *et al.* (1989)	[70]	*Chlorella pyrenoidosa *(water green algae)	NEL > 9.1 mg L^-1^	
Halley *et al.* (1989), Boxall *et al.* (2002)	[70, 167]	*Chlorella pyrenoidosa *(water green algae)	14 d LC_50_ >10000 mg L^-1^	
Garric *et al.* (2007)	[215]	*Pseudokirchneriella subcapitata* (green algae)	EC_50_ (growth rate) >4000 µg L^-1^ LOEC (growth rate) = 1250 µg L^-1^ NOEC (growth rate) = 391 µg L^-1^ EC_50_ (yield) >4000 µg L^-1^ LOEC (yield) = 1250 µg L^-1^ NOEC (yield) = 391 µg L^-1^	Static acute growth rate and yield
Boxall *et al.* (2002)	[167]	Higher plants	0.56 mg.kg^-1^	NOEC

Dosages for cattle are: 500 (pour-on), 200 (injectable) or 200 (oral) µg kg^-1^ b.w.

**Table 4.** Ecotoxicity of Eprinomectin (EPR) to Aquatic and Terrestrial Organisms (for key to Dosages, see Table Footnotes)

Source	Reference	Test organism	Toxicity / response	Conditions
		VERTEBRATES		
Boxall *et al.* (2002), Merck and Co (1996)	[167, 230]	Bobwhite quail	272 mg kg^-1^	14 d LC_50_
Boxall *et al.* (2002), Merck and Co (1996)	[167, 230]	Bobwhite quail	<62.5 mg kg^-1^	14 d NOEC
Boxall *et al.* (2002), Merck and Co (1996)	[167, 230]	Bobwhite	1813 mg kg^-1^	(dietary) 8 d LC_50_
Boxall *et al.* (2002), Merck and Co (1996)	[167, 230]	Bobwhite	1000 mg kg^-1^	(dietary) 8 d NOEC
Boxall *et al.* (2002), Merck and Co (1996)	[167, 230]	Mallard duck	24 mg kg^-1^	14 d LC_50_
Boxall *et al.* (2002), Merck and Co (1996)	[167, 230]	Mallard duck	<7.8 mg kg^-1^	14 d NOEC
Boxall *et al.* (2002), Merck and Co (1996)	[167, 230]	Mallard duck	447 mg kg^-1^	(dietary) 8 d LC_50_
Boxall *et al.* (2002), Merck and Co (1996)	[167, 230]	Mallard duck	<100 mg kg^-1^	(dietary) 8 d NOEC
Merck and Co. (1996)	[230]	*Onchorhynchus mykiss* (Rainbow trout)	1.2 mg L^-1^	96 h LC_50_
Merck and Co. (1996)	[230]	*Onchorhynchus mykiss* (Rainbow trout)	0.37 mg L^-1^	96 h NOEC
Merck and Co. (1996)	[230]	*Lepomis macrochirus* (Bluegill)	0.37 mg L^-1^	96 h LC_50_
Merck and Co. (1996)	[230]	*Lepomis macrochirus* (Bluegill)	0.14 mg L^-1^	96 h NOEC
		TERRESTRIAL INVERTEBRATES		
Wardhaugh *et al.* (2001)	[231]	*Onthophagus taurus* (dung beetle)	High larval mortality for 1-2 weeks post-treatment. Newly emerge beetles susceptible to residues in dung voided on day 3	Pour-on (cattle)
Floate *et al.* (2001)	[178]	*Musca domestica* (house fly)	< control at weeks 1; 2 and 4.	Pour-on (cattle); Survival of flies developing in dung
Wardhaugh *et al.* (2001)	[180]	*Musca inferior* (diptera)	Reduced larval survival for 9-13 days post-treatment	Pour-on (cattle)
Floate *et al.* (2001)	[178]	*Haematobia irritans* (horn fly)	No emergence at week 1; < control at wks 2 and 4.	Pour-on (cattle); Survival of flies developing in dung
Floate *et al.* (2001)	[178]	*Stomoxys calcitrans* (stable fly)	< control until week 4	Pour-on (cattle); Survival of flies developing in dung
Lumaret *et al.* (2005)	[229]	*Neomyia cornicina* (dung-dwelling Diptera)	NOEC = 7 ± 5 mg g-1 null emergence until day 7 after treatment; high larval mortality until day 12	Pour-on (cattle)
Wardhaugh *et al.* (2001)	[180]	*Orthelia timorensis* (dung-dwelling Diptera)	Reduced larval survival for 9-13 days post-treatment	Pour-on (cattle)
Halley *et al.* (2005)	[236]	*Lumbricus terrestris* (earthworm)	Mortality and behaviour unaffected by residues in dung voided ≤ 14 days post-treatment	Pour-on (cattle) 28 day test period in lab study
Boxall *et al.* (2002), Merck and Co (1996)	[167, 230]	*Lumbricus terrestris* (earthworm)	295 mg kg^-1^	28 d NOEC (mortality)
Boxall *et al.* (2002), Merck and Co (1996)	[167, 230]	*Lumbricus terrestris* (earthworm)	90.8 mg kg^-1^	28 d LC_50_ (weight)
		AQUATIC INVERTEBRATES		
Merck and Co. (1996)	[230]	*Daphnia magna* (Cladocera)	0.002 mg L^-1^	24 h EC_50_
Merck and Co. (1996)	[230]	*Daphnia magna* (Cladocera)	0.0005 mg L^-1^	48 h EC_50_
		PLANTS		
Merck and Co. (1996)	[230]	*Selenastrum capricornutum* (green algae)	29 mg L^-1^	14 d MIC
Merck and Co. (1996)	[230]	*Selenastrum capricornutum* (green algae)	7 mg L^-1^	14 d NOEC
Boxall *et al.* (2002), Merck and Co (1996)	[167, 230]	Cucumber, lettuce, soybean, ryegrass, tomato, wheat	1300 mg kg^-1^	NOEC germination
Boxall *et al.* (2002), Merck and Co (1996)	[167, 230]	Cucumber, soybean	9.5 mg kg^-1^	NOEC root elongation
Boxall *et al.* (2002), Merck and Co (1996)	[167, 230]	Lettuce, ryegrass, tomato, wheat	8.5 mg kg^-1^	NOEC root elongation
Boxall *et al.* (2002), Merck and Co (1996)	[167, 230]	Cucumber, ryegrass, tomato, wheat	0.47 mg kg^-1^	NOEC shoot length and root weight
Boxall *et al.* (2002), Merck and Co (1996)	[167, 230]	Lettuce, soybean	6.5 mg kg^-1^	NOEC shoot length and root weight
		MICRO-ORGANISMS		
Boxall *et al.* (2002), Merck and Co (1996)	[167, 230]	26 microbial species	1000 mg kg^-1^	NOEC antimicrobial activity

Dosages for cattle are: 500 (pour-on), 200 (injectable) or 200 (oral) µg kg^-1^ b.w.

**Table 5.** Ecotoxicity of Doramectin (DOR) to Terrestrial and Aquatic Organisms (for key to Dosages, see Table Footnotes)

Source	Reference	Test Organism	Toxicity / Response	Conditions
		VERTEBRATES		
Boxall *et al.* (2002), Pfizer (1996)	[167, 240]	*Lepomis macrochirus* (Bluegill)	0.01 mg L^-1^	96 h LC_50_
Boxall *et al.* (2002), Pfizer (1996)	[167, 240]	*Lepomis macrochirus* (Bluegill)	0.002 mg L^-1^	96 h NOEC
		TERRESTRIAL INVERTEBRATES		
Webb *et al.* (2010)	[333]	*Aphodius spp*. (dung beetles)	Increased capture of adults in pastures with avermectin-treated versus untreated cattle; preferential colonization of dung from untreated versus DOR-treated cattle	*Pour-on* (cattle)
Dadour *et al.* (2000)	[160]	*Onthophagus binodis* (dung beetle)	Reduced survival of newly emerged adults fed dung voided 9 days post-treatment; reduced ovarian condition and egg production of newly emerged adults fed dung voided up 3 &amp;amp; 6 days post-treatment; reduced egg-to-adult survival in dung voided 3 &amp;amp; 6 days post-treatment	*Injectable* (cattle)
Boxall *et al.* (2002), Pfizer (1996)	[167, 240]	*Digitonthophagus gazella* (dung beetle)	12.5 mg kg^-1^ dung	LC_50_
Boxall *et al.* (2002), Pfizer (1996)	[167, 240]	*Digitonthophagus gazella* (dung beetle)	38.2 mg kg^-1^ dung	LC_90_
Floate *et al.* (2001)	[178]	*Musca domestica* (house fly)	No emergence at week 1; < control at wks 2 and 4 after treatment.	*Pour-on* (cattle) Survival of flies developing in dung.
Farkas *et al.* (2003)	[264]	*Musca domestica* (house fly)	Reduced larva-to-adult survival in dung voided ≤ 23 (cattle) or ≤ 15 (swine) days post-treatment	Injectable (cattle) Injectable (swine) with 300 µg kg^-1^ b.w.
Wardhaugh *et al.* (2001)	[180]	*Musca inferior* (Diptera)	Reduced larval survival for 9-13 days post-treatment	Pour-on (cattle)
Floate *et al.* (2001)	[178]	*Haematobia irritans* (horn fly)	No emergence 4 weeks after treatment.	Pour-on (cattle); 0.5 mg DOR.kg^-1^ bw. Survival of flies developing in dung
Boxall *et al.* (2002), Pfizer (1996)	[167, 240]	*Haematobia irritans* (horn fly)	3 mg kg^-1^ dung	LC_90_
Andress *et al.* (2000)	[348]	*Haematobia irritans* (horn fly)	Suppression of adult flies on cattle for 8-9 weeks after single application	Pour-on (cattle)
Webb *et al.* (2007)	[349]	*Scatophaga stercoraria* (Diptera)	No difference in recovery of adult flies from pastures with avermectin-treated versus untreated cattle; increased fluctuating wing asymmetry for flies from pastures of DOR-treated cattle	Pour-on (cattle)
Floate *et al.* (2001)	[178]	*Stomoxys calcitrans* (stable fly)	no emergence at wk 1 < control at weeks 2 and 4	Pour-on (cattle); 0.5 mg DOR.kg^-1^ bw. Survival of flies developing in dung.
Wardhaugh *et al.* (2001)	[180]	*Chrysomya bezziana* (Old World screw-worm fly OWS)	DOR gave 7 days protection	Pour-on formulation at 1ml 10 kg^-1^ b.w.
Wardhaugh *et al.* (2001)	[180]	*Orthelia timorensis* (Diptera)	Reduced larval survival for 9-13 post-treatment	Pour-on (cattle)
Kolar *et al.* (2008)	[146]	*Folsomia candida* (springtail)	28 d LC_50_ (survival) >300 mg kg^-1^ d.s. 28 d EC_50_ (reproduction) = 26 mg kg^-1^ d.s. 28 d EC_50_ (reproduction) = 42 mg kg^-1^ d.s. 28 d NOEC = 30 mg.kg^-1^ d.s.	Soil; mortality, reproduction after 28 days of exposure
Kolar *et al.* (2008)	[146]	*Folsomia candida* (springtail)	28 d LC_50_ (survival) >2.5 mg kg^-1^ d.f. 28 d EC_50_ >2.5 mg kg^-1^ d.f. 28 d NOEC >2.5 mg kg^-1^ d.f. No effect on reproduction	concentrations of DOR in sheep faeces; mortality, reproduction after 28 days of exposure
Kolar *et al.* (2008)	[146]	*Porcellio scaber* (isopod)	21 d LC_50_ (survival) >300 mg kg^-1^ d.s.	Soil; mortality after 21 days of exposure
Kolar *et al.* (2008)	[146]	*Eisenia andrei* (earthworm)	28 d LC_50_ (survival) = 228 mg kg^-1^ d.s. 28 d LOEC (weight loss) = 25 mg kg^-1^ d.s. 28 d NOEC weight loss) = 8.4 mg kg^-1^ d.s.	Soil; mortality, weight loss after 28 days of exposure
Kolar *et al.* (2008)	[146]	*Eisenia andrei* (earthworm)	28 d EC_50_ >2.5 mg kg^-1^ d.f. 28 d LC_50_ >2.5 mg kg^-1^ d.f. no effect on reproduction 28 d NOEC > 2.5 mg kg^-1^ d.f..	concentrations of DOR in sheep faeces; mortality, reproduction after 28 days of exposure
Boxall *et al.* (2002), Pfizer (1996)	[167, 240]	*Eisenia foetida* (earthworm)	28 d LC_50_ > 1000 mg kg^-1^	
Boxall *et al.* (2002), Pfizer (1996)	[167, 240]	*Eisenia foetida* (earthworm)	2 mg kg^-1^	28 d NOEC (growth)
Boxall *et al.* (2002), Pfizer (1996)	[167, 240]	*Eisenia foetida* (earthworm)	4 mg kg^-1^	28 d LOEC (growth)
Kolar *et al.* (2008)	[146]	*Enchytraeus crypticus* enchytraeid)	28 d LC_50_ (survival) >300 mg kg^-1^ d.s. 28 d EC_50_ (reproduction) = 79 mg kg^-1^ d.s. 28 d EC_50_ (reproduction) = 170 mg kg^-1^ d.s. 28 d NOEC = 100 mg kg^-1^ d.s.	Soil; mortality, reproduction after 28 days of exposure
Kolar *et al.* (2008)	[146]	*Enchytraeus crypticus* (enchytraeid)	28 d EC_50_ (reproduction) = 2.2 mg kg^-1^ d.f. 28 d LC_50_ >2.5 mg kg^-1^ d.f. 28 d NOEC = 2.5 mg kg^-1^ d.f.	concentrations of DOR in sheep faeces; mortality, reproduction after 28 days of exposure
		AQUATIC INVERTEBRATES		
Boxall *et al.* (2002), Pfizer (1996)	[167, 240]	*Daphnia magna* (Cladocera)	0.0001 mg L^-1^	48 h EC_50_
Boxall *et al.* (2002), Pfizer (1996)	[167, 240]	*Daphnia magna* (Cladocera)	0.000025 mg L^-1^	48 h NOEC
		PLANTS		
Boxall *et al.* (2002), Pfizer (1996)	[167, 240]	*Selenastrum* (green algae)	Not acutely toxic	Not acutely toxic
Boxall *et al.* (2002), Pfizer (1996)	[167, 240]	Corn	840 mg kg^-1^	% germination NOEC
Boxall *et al.* (2002), Pfizer (1996)	[167, 240]	Corn	840 mg kg^-1^	% root elongation NOEC
Boxall *et al.* (2002), Pfizer (1996)	[167, 240]	Corn	980 mg kg^-1^	% seedling growth NOEC
Boxall *et al.* (2002), Pfizer (1996)	[167, 240]	Cucumber	840 mg kg^-1^	% germination NOEC
Boxall *et al.* (2002), Pfizer (1996)	[167, 240]	Cucumber	840 mg kg^-1^	% root elongation NOEC
Boxall *et al.* (2002), Pfizer (1996)	[167, 240]	Cucumber	53 - 130 mg kg^-1^	% seedling growth NOEC
Boxall *et al.* (2002), Pfizer (1996)	[167, 240]	Ryegrass	6.6 mg kg^-1^	% germination NOEC
Boxall *et al.* (2002), Pfizer (1996)	[167, 240]	Ryegrass	1.6 mg kg^-1^	% root elongation NOEC
Boxall *et al.* (2002), Pfizer (1996)	[167, 240]	Ryegrass	< 33 mg kg^-1^	% seedling growth NOEC
Boxall *et al.* (2002), Pfizer (1996)	[167, 240]	Soy bean	990 mg kg^-1^	% germination NOEC
Boxall *et al.* (2002), Pfizer (1996)	[167, 240]	Soy bean	990 mg kg^-1^	% root elongation NOEC
Boxall *et al.* (2002), Pfizer (1996)	[167, 240]	Soy bean	47 mg kg^-1^	% seedling growth NOEC
Boxall *et al.* (2002), Pfizer (1996)	[167, 240]	Tomato	840 mg kg^-1^	% germination NOEC
Boxall *et al.* (2002), Pfizer (1996)	[167, 240]	Tomato	840 mg kg^-1^	% root elongation NOEC
Boxall *et al.* (2002), Pfizer (1996)	[167, 240]	Tomato	47 mg kg^-1^	% seedling growth NOEC
Boxall *et al.* (2002), Pfizer (1996)	[167, 240]	Wheat	57 mg kg^-1^	% germination NOEC
Boxall *et al.* (2002), Pfizer (1996)	[167, 240]	Wheat	57 mg kg^-1^	% root elongation NOEC
Boxall *et al.* (2002), Pfizer (1996)	[167, 240]	Wheat	47 mg kg^-1^	% seedling growth NOEC
		MICRO-ORGANISMS		
Boxall *et al.* (2002), Pfizer (1996)	[167, 240]	Clostridium perfringens	40 mg L^-1^	MIC
Boxall *et al.* (2002), Pfizer (1996)	[167, 240]	Nostoc	60 mg L^-1^	MIC
Boxall *et al.* (2002), Pfizer (1996)	[167, 240]	Aspergillus flavus	600 mg L^-1^	MIC
Boxall *et al.* (2002), Pfizer (1996)	[167, 240]	Pseudomonas aeruginosa	800 mg L^-1^	MIC
Boxall *et al.* (2002), Pfizer (1996)	[167, 240]	Chaetomium globosum	800 mg L^-1^	MIC

Dosages for cattle are: 500 (pour-on), 200 (injectable) or 200 (oral) µg kg^-1^ b.w.

**Table 6.** Ecotoxicity of Emamectin Benzoate to Aquatic and Terrestrial Organisms

Source	Reference	Test Organism	Toxicity / Response	Conditions
		VERTEBRATES		
McHenery & Mackie (1999), Schering-Plough Anim. Health (2002)	[246-247]	*Oncorhynchus mykiss* (Rainbow trout)	96 h LC_50_ = 174 µg L^-1^ 96 h NOEC = 48.7 µg L^-1^	
Chukwudebe *et al.* (1996)	[257]	*Lepomis macrochirus* (Bluegill sunfish)	96 h LC_50_ = 180 µg L^-1^ 96 h NOEC = 87 µg L^-1^	
McHenery & Mackie (1999), Schering-Plough Anim. Health (2002)	[246-247]	*Pimephales promelas* (Fathead minnow)	96 h EC_50_ (adult) = 194 µg L^-1^ 96 h NOEC (adult) = 156 µg L^-1^ 96 h LC_50_ (MATC) = 18 µg L^-1^ 96 h NOEC (MATC) = 12 µg L^-1^ 96 h LOEC = 28 µg L^-1^	mortality (96 h); early life stages (96 h): MATC and LOEC
McHenery & Mackie (1999);, Schering-Plough Anim. Health (2002)	[246-247]	*Cyprinodon variegatus* (Sheepshead minnow)	96 h LC_50_ = 1,350 µg L^-1^	exposition 96 h
		TERRESTRIAL INVERTEBRATES		
Ahmad *et al.* (2008)	[144]	*Spodoptera litura* (Lepidoptera: Noctuidae)	72 h LC_50_ = 0.03 (lab. pop.) - 2.31 mg L^-1^	Mortality assessed after 72 h exposure to emamectin. Comparison laboratory susceptible population with field populations
Argentine *et al.* (2002)	[256]	*Spodoptera exigua* (Lepidoptera)	6 d LC_50_ = 0.026 mg L^-1^ 6 d LC_90_ = 0.305 mg L^-1^	Mortality recorded 6 d after application (contact with treated plant)
Argentine *et al.* (2002)	[256]	*Spodoptera frugiperda* (Lepidoptera)	6 d LC_50_ = 0.003 mg L^-1^ 6 d LC_90_ = 0.007 mg L^-1^	Mortality recorded 6 d after application (contact)
Argentine *et al.* (2002)	[256]	*Heliothis virescens* (Lepidoptera)	6 d LC_50_ = 0.003 mg L^-1^ 6 d LC_90_ = 0.009 mg L^-1^	Mortality recorded 6 d after application (contact)
Argentine *et al.* (2002)	[256]	*Plutella xylostella* (Lepidoptera)	6 d LC_50_ = 0.001 mg L^-1^ 6 d LC_90_ = 0.005 mg L^-1^	Mortality recorded 6 d after application (contact)
Argentine *et al.* (2002)	[256]	*Trichoplusia ni* (Lepidoptera)	6 d LC_50_ = 0.007 mg L^-1^ 6 d LC_90_ = 0.013 mg L^-1^	Mortality recorded 6 d after application (contact)
Argentine *et al.* (2002)	[256]	*Pseudoplusia includens* (Lepidoptera)	6 d LC_50_ = 0.006 mg L^-1^ 6 d LC_90_ = 0.022 mg L^-1^	Mortality recorded 6 d after application (contact)
		AQUATIC INVERTEBRATES		
Waddy *et al.* (2007)	[255]	*Homarus americanus* (American lobster)	NOEL (moult cycle) = 0.12 mg a.i. g^-1^ lobster LOEL (moult cycle) = 0.22 mg a.i. g^-1^ lobster	dose response of ovigerous lobsters to emamectin benzoate
McHenery & Mackie (1999), Schering-Plough Anim. Health (2002)	[246-247]	*Nephrops norvegicus* (Decapoda)	96 h LC_50_ = 983 µg L^-1^ 192 h LC_50_ = 572 µg L^-1^ 96 h NOEC = 814 µg L^-1^ 192 h NOEC =440 µg L^-1^ 96 h LC_50_ >68.2 mg kg^-1^ 192 h LC_50_ >68.2 mg kg^-1^ 96 h NOEC = 68.2 mg L^-1^ 192 h NOEC = 68.2 mg kg^-1^	water exposure (96 h and 192 h) Feed exposure (96 h and 192 h) (concentration reported µg.kg^-1^feed)
McHenery & Mackie (1999), Schering-Plough Anim. Health (2002)	[246-247]	*Crangon crangon* (Decapoda)	96 h LC_50_ = 242 µg L^-1^ 192 h LC_50_ = 161 µg L^-1^ 96 h NOEC = 161 µg L^-1^ 192 h NOEC = 161 < µg L^-1^ 96 h LC_50_ >69.3 mg L^-1^ 192 h LC_50_ >69.3 mg L^-1^ 96 h NOEC = 69.3 mg L^-1^ 192 h NOEC = 69.3 mg L^-1^	water exposure (96 h and 192 h) Feed exposure (96 h and 192 h) (concentration reported µg.kg^-1^ feed)
Bravo *et al.* (2008)	[248]	*Caligus rogercresseyi* (Copepod)	EC_50_ = 57-203 µg L^-1^ (µg kg^-1^) (summer season) EC_50_ = 202-870 µg L^-1^ (µg kg^-1^) (winter season) EC_50_ (naive individuals) = 34 µg L^-1^ (µg kg^-1^)	End point: immobilization; resistance in copepods exposed to emamectin benzoate
Willis & Ling (2003)	[249]	*Acartia clausi* (Copepod)	48 h EC_50_ (N6) = 0.57 µg L^-1^ 96 h EC_50_ (N6) = 0.48 µg L^-1^ 48 h EC_50_ (C1) = 0.28 µg L^-1^ 96 h EC_50_ (C1) = 0.13 µg L^-1^ 48 h EC_50_ (C6) = 0.29 µg L^-1^ 96 h EC_50_ (C6) = 5.27 µg L^-1^ NOEC (adult) = 0.05 µg L^-1^ LOEC (adult) = 0.16 µg L^-1^ Egg production reduced for concentrations of 0.16 and 0.5 µg L^-1^	three life stages: nauplii (N6), copepodites (instars C1 and C6) and adults exposed 48 h and 96 h to emamectin benzoate Daily egg production after 96 h exposition
McHenery & Mackie (1999), Schering-Plough Anim. Health (2002)	[246-247]	*Artemia salina* (Anostraca)	LC_50_ (6 h) = 1.73 mg L^-1^	IC100
McHenery & Mackie (1999), Schering-Plough Anim. Health (2002)	[246-247]	*Mysidopsis bahia* (Mysidacea)	96 h LC_50_ = 0.04 mg L^-1^ 96 h NOEC = 0.02 µg L^-1^	
Willis & Ling (2003)	[249]	*Pseudocalanus elongatus* (Copepod)	48 h EC_50_ (N6) = 0.12 µg L^-1^ 48 h EC_50_ (C1) = 0.14 µg L^-1^ 96 h EC_50_ (C1) = 0.17 µg L^-1^ 48 h EC_50_ (C6) = 0.45 µg L^-1^ 96 h EC_50_ (C6) = 10.9 µg L^-1^	life stages: nauplii (N6) and copepodites (C1 and C6) exposed 48 h and 96 h to emamectin benzoate
Willis & Ling (2003)	[249]	*Temora longicornis* (Copepod)	48 h EC_50_ (N6) = 0.23 µg L^-1^ 48 h EC_50_ (C1) = 0.41 µg L^-1^ 48 h EC_50_ (C6) = 2.8 µg L^-1^	life stages: nauplii (N6) and copepodites (C1 and C6) exposed 48 h to emamectin benzoate
Willis & Ling (2003)	[249]	*Oithona similis* (Copepod)	48 h EC_50_ (N6) >15.8 µg L^-1^ 96 h EC_50_ (N6) >15.8 µg L^-1^ 48 h EC_50_ (C1) = 15.86 µg L^-1^ 96 h EC_50_ (C1) = 14.8 µg L^-1^ 48 h EC_50_ (C6) = 232 µg L^-1^ 96 h EC_50_ (C6) = 113 µg L^-1^	life stages: nauplii (N6) and copepodites (C1 and C6) exposed 48 h and 96 h to emamectin benzoate
McHenery & Mackie (1999), Schering-Plough Anim. Health (2002)	[246-247]	*Corophium volutator* (Amphipoda) (mud shrimp)	10 d LC_50_ = 6.32 µg L^-1^ 10 d NOEC = 3.2 µg L^-1^ 10 d LC_50_ = 193 µg kg^-1^ 10 d NOEC =115 µg kg^-1^	water (µg. L^-1^) sediment (µg.kg^-1^wet sediment)
McHenery & Mackie (1999), Schering-Plough Anim. Health (2002)	[246-247]	*Daphnia magna* (Cladocera)	48 h LC_50_ (48 h) = 1.0 µg L^-1^ 48 h NOEC (48 h) = 0.3 µg L^-1^ 21 d LC_50_ reprod. = 0.16 µg L^-1^ 21 d NOEC reprod. = 0.09 µg L^-1^ 21 d MATC = 0.12 µg L^-1^ 21 d LC_50_ feed = 0.13 µg kg^-1^	mortality (48 h); reproduction - LOEC (21 d); MATC (maximum acceptable toxicant concentration)(21 d); LC_50_ (adults) feed exposure (21 d) (µg kg^-1^feed)
McHenery & Mackie (1999), Schering-Plough Anim. Health (2002)	[246-247]	*Capitella capitata* (Polychaete worm, Capitellidae)	21 d LC_50_ = 1.04 mg L^-1^ 21 d NOEC = 460 µg L^-1^	Exposition 21 d
McHenery & Mackie (1999), Schering-Plough Anim. Health (2002)	[246-247]	*Arenicola marina* (lugworm, Polychaeta)	10 d LC_50_ = 111 µg kg^-1^ 10 d NOEC = 56.0 µg kg^-1^	sediment (µg kg^-1^ wet sediment)

**Table 7.** Ecotoxicity of Moxidectin (MOX) to terrestrial and aquatic organisms (for key to dosages, see Table footnotes).

Source	Reference	Test organism	Toxicity / response	Conditions
		VERTEBRATES		
Fort Dodge (1997)	[270]	Bobwhite quail	278 mg kg^-1^	21 d acute oral LD_50_
Fort Dodge (1997)	[270]	Mallard duck	365 mg kg^-1^	21 d acute oral LD_50_
Fort Dodge (1997)	[270]	Chicken	283 mg kg^-1^	14 d acute oral LD_50_
Fort Dodge (1997)	[270]	*Lepomis macrochirus* (Bluegill)	0.0006 mg L^-1^ <0.0005 mg L^-1^	96 h LC_50_ 96 h NOEC
Fort Dodge (1997)	[270]	*Onchorhynchus mykiss* (Rainbow trout)	0.0002 mg L^-1^ <0002 mg L^-1^	96 h LC_50_ 96-h NOEC
		TERRESTRIAL INVERTEBRATES		
Iwasa *et al.* (2008)	[266]	*Caccobius jessoensis* (dung beetle)	No effect on egg-adult survival	Pour-on (cattle)
Fincher & Wang (1992)	[269]	*Digitonthophagus gazella* (dung beetle)	no adverse effects on adult emergence	Injectable (cattle) Reproduction
Fort Dodge (1997)	[270]	*Digitonthophagus gazella* (dung beetle)	No effect on colonisation nor larval development NOEC >0.50 mg kg^-1^	Injectable (cattle); adult NOEC
Doherty *et al.* (1994)	[66]	*Digitonthophagus gazella* (dung beetle)	Effects at concentrations ≥ 256 µg kg^-1^	Sensitivity of larvae
Fort Dodge (1997)	[270]	*Digitonthophagus gazella* (dung beetle)	0.256 mg kg^-1^	progeny EC_50_
Wardhaugh *et al.* (2001)	[231]	*Onthophagus taurus* (dung beetle)	Dung voided from 3-70 days post-treatment showed no detectable effects on development or survival	Pour-on (cattle)
Fincher & Wang (1992)	[269]	*Euoniticellus intermedius* (dung beetle)	no adverse effects on adult emergence	Injectable (cattle); reproduction
Fort Dodge (1997)	[270]	*Euoniticellus intermedius* (dung beetle)	>0.50 mg kg^-1^ 0.47 mg kg^-1^ >0.27 mg kg^-1^	adult NOEC progeny EC_50_ progeny NOEC
Fort Dodge (1997)	[270]	*Aphodius larvae* (dung beetle)	No effect on egg-adult survival	Injectable (cattle)
Hempel *et al.* (2006)	[193]	*Aphodius constans* (dung beetle)	LC_50_ = 4.0 - 5.4 mg kg^-1^ dung (d.w.)	
Wardhaugh *et al.* (1996)	[174]	*Musca vetustissima* (bush fly)	MOX has no effect on larval survival	Injectable (steers)
Floate *et al.* (2001)	[178]	*Stomoxys calcitrans* (stable fly)	no effect	Spiked dung
Wardhaugh *et al.* (1996)	[174]	*Musca domestica (house* fly)	MOX has no effect on larval survival	Injectable (steers)
Wardhaugh *et al.* (2001)	[180]	*Musca inferior* (Diptera)	MOX has no effect on larval survival	Pour-on (cattle)
Floate *et al.* (2001)	[178]	*Musca domestica* (house fly)	no effect	Pour-on (cattle); Survival of flies developing in dung
Farkas *et al.* (2003)	[264]	*Musca domestica* (house fly)	no effect on larva-to-adult survival	Injectable (cattle); Injectable (swine) 300 µg.kg^-1^ b.w.
Floate *et al.* (2001)	[178]	*Haematobia irritans* (horn fly)	< control at wks 1, 2 and 4.	Pour-on (cattle); Survival of flies developing in dung
Fort Dodge (1997)	[270]	*Haematobia irritans exigua* (horn fly)	0.134 mg kg^-1^ 0.064 mg kg^-1^	EC_50_ NOEC
Iwasa *et al.* (2008)	[266]	*Haematobia irritans* (horn fly)	Reduced egg-adult survival in dung voided ≤ 7 days post-treatment	Pour-on (cattle)
Iwasa *et al.* (2008)	[266]	*Neomyia cornicina* (dung-dwelling Diptera)	Reduced egg-adult survival in dung voided ≤ 7 days post-treatment	Pour-on (cattle)
Wardhaugh *et al.* (2001)	[180]	*Orthelia timorensis* (Diptera)	MOX has no effect of larval survival	Pour-on (cattle)
Fort Dodge (1997)	[270]	*Eisenia foetida* (earthworm)	37.2 mg kg^-1^ substrate	28 d subacute LC_50_
		AQUATIC INVERTEBRATES		
Fort Dodge (1997)	[270]	*Daphnia magna* (Cladocera)	0.00003 mg L^-1^	48 h EC_50_
Fort Dodge (1997)	[270]	*Daphnia magna* (Cladocera)	0.00001 mg L^-1^	48 h NOEC
		PLANTS AND ALGAE		
Fort Dodge (1997)	[270]	*Selenastrum capricornutum* (green algae)	0.087 mg L^-1^	72 h EC_50_
Fort Dodge (1997)	[270]	Abutilon theophrasti (velvetleaf), Ambrosia artemisiifolia (common ragweed), Avena fatua (wild oats), Brassica kaber (wild mustard), Calystegia arvensis (hedge bindweed), Cyperus rotundus (purple nutsedge), Digitaria sanguinalis (large crabgrass), Echinochloa crus-galli (barnyardgrass), Elytrigia repens (quackgrass), Ipomoea sp. (morningglory), Setaria viridis (green foxtail), Sida spinosa (prickly sida).	4 kg ha^-1^	phytotoxicity NOEC

Dosages for cattle are: 500 (pour-on), 200 (injectable) or 200 (oral) µg kg^-1^ b.w.

**Table 8.** Ecotoxicity of Spinosad (SPI) to Aquatic and Terrestrial Organisms

Source	Reference	Test organism	Toxicity / response	Duration and Conditions
		VERTEBRATES		
WHO (2008)	[293]	Rat, m. & f.	LD_50_ ≥ 3738 mg a.i. kg^-1^ b.w. (m.) LD_50_ > 5000 mg kg^-1^ b.w. (f.)	OECD guideline 401 acute oral toxicity, 1987
WHO (2008)	[293]	Rat, m. & f.	LD_50_ > 5.18 mg L^-1^	EC test guideline (EC method B.2 acute toxicity) (inhalation, 4h), 1984
WHO (2008)	[293]	Rat, m. & f.	NOAEL = 9.5 mg m3	14-d inhalation, 15-d recovery
WHO (2008)	[293]	Rat, m. & f.	NOAEL = 8.6 mg kg^-1^ b.w. d^-1^ LOAEL = 42.7 mg kg^-1^ b.w. d^-1^	13-week oral; repeated administration
WHO (2008)	[293]	Rat, m. & f.	NOAEL = 7.7 mg kg^-1^ b.w. d^-1^ LOAEL = 39.1 mg kg^-1^ b.w. d^-1^	13-week oral; repeated administration
WHO (2008)	[293]	Rat, m. & f.	NOAEL = 2.4 mg kg^-1^ b.w. d^-1^ LOAEL = 11.4 mg kg^-1^ b.w. d^-1^ No carcinogenic potential	2-year oral, combined chronic toxicity and carcinogenicity
WHO (2008)	[293]	Rat	NOAEL = 10 mg kg^-1^ b.w. d^-1^ Reproduction NOAEL = 100 mg kg^-1^ b.w. d^-1^	2-generation; reproductive study
WHO (2008)	[293]	Rat	Maternal NOAEL = 50 mg kg^-1^ b.w. d^-1^ Developmental NOAEL = 200 mg kg^-1^ b.w. d^-1^ No teratogenic potential	Teratogenicity
WHO (2008)	[293]	Rat, m. & f.	No evidence of neurotoxicity in acute, sub-chronic and chronic studies	Neurotoxicity
WHO (2008)	[293]	Mouse, m. & f.	LD_50_ > 5000 mg kg^-1^ b.w. (m. & f.)	OECD guideline 401 acute oral toxicity, 1987
WHO (2008)	[293]	Mouse, m. & f.	NOAEL = 7.5 mg kg^-1^ b.w. d^-1^ LOAEL = 22.5 mg kg^-1^ b.w. d^-1^	3-month oral
WHO (2008)	[293]	Mouse, m. & f.	NOAEL = 11.4 mg kg^-1^ b.w. d^-1^ LOAEL = 32.7 mg kg^-1^ b.w. d^-1^ No carcinogenic potential	18-month oral, combined chronic toxicity and carcinogenicity
WHO (2008)	[293]	Dog, m. & f.	NOAEL = 4.89 mg kg^-1^ b.w. d^-1^ LOAEL = 9.73 mg kg^-1^ b.w. d^-1^	13-week oral; repeated administration
WHO (2008)	[293]	Dog, m. & f.	NOAEL = 2.68 mg kg^-1^ b.w. d^-1^ LOAEL = 8.22 mg kg^-1^ b.w. d^-1^	12-month oral
WHO (2008)	[293]	Rabbit, m.& f.	LD_50_ > 5000 mg kg^-1^ b.w. (m. & f.)	OECD guideline 402 acute dermal toxicity, 1987
WHO (2008)	[293]	Rabbit, m. & f.	NOAEL = 1000 mg kg^-1^ b.w.	21-d dermal; repeated administration (sub-acute to chronic
WHO (2008)	[293]	Rabbit	Maternal NOAEL = 10 mg kg^-1^ b.w. d^-1^ Developmental NOAEL = 50 mg kg^-1^ b.w. d^-1^ No teratogenic potential	Teratogenicity
WHO (2008)	[293]	*Oncorhynchus mykiss* (rainbow trout)	96 h LC^50^ = 27 mg L^-1^	96 h, FIFRA 72^-1^ & OECD 203, 12.5 ± 0.5°C; Acute toxicity, static
WHO (2008)	[293]	*Oncorhynchus mykiss* (rainbow trout)	80 d NOEC = 0.5 mg L^-1^	80-d, FIFRA 72-4(a) & OECD 210 (12 ± 2°C); Early life-stage toxicity, flow through
WHO (2008)	[293]	*Lepomis macrochirus* (Bluegill sunfish)	96 h LC^50^ = 5.94 mg L^-1^	96-h, FIFRA 72^-1^ & OECD 203 (21-22.1°C); acute toxicity, static
WHO (2008)	[293]	*Cyprinus carpio* (common Carp)	96 h LC^50^ = 4 mg L^-1^	96-h, FIFRA 72^-1^ & OECD 203 (24.5-25.5°C); acute toxicity, flow through
WHO (2008)	[293]	*Cyprinus carpio* (common Carp)	96 h LC^50^ > 49 mg L^-1^	96-h, OECD 203 (22 ± 2°C), 480 g.L^-1^ SC; acute toxicity, static
[Dow AgroSciences] (2010)	[308]	*Cyprinodon variegatus* (sheepshead Minnow)	96 h LC^50^ = 7.9 mg L^-1^	96-h acute
WHO (2008)	[293]	*Colinus virginianus* (bobwhite quail)	14 d LD_50_ > 2000 mg kg^-1^ b.w.	14-d, FIFRA 71^-1^; acute oral toxicity
WHO (2008)	[293]	*Colinus virginianus* (bobwhite quail)	5 d LC^50^ > 5253 mg a.i. kg^-1^ diet	5-d, FIFRA 71-2 & OECD 205, 88% A+D spinosyns; short-term dietary toxicity
WHO (2008)	[293]	*Colinus virginianus* (bobwhite quail)	21 week NOEC = 550 mg kg^-1^ diet	21-week, FIFRA 71-4(a) & OECD 206; reproduction study
WHO (2008)	[293]	*Anas platyrhynchos* (mallard duck)	14 d LD_50_ > 2000 mg kg^-1^ b.w.	14-d, FIFRA 71^-1^; Acute oral toxicity
WHO (2008)	[293]	*Anas platyrhynchos* (mallard duck)	5 d LC^50^ > 5156 mg k^-1^g diet	5-d, FIFRA 71-2 & OECD 205; short-term dietary toxicity
WHO (2008)	[293]	*Anas platyrhynchos* (mallard duck)	21 week NOEC = 550 mg kg^-1^ diet	21-week, FIFRA 71-4(b) & OECD 206; Reproduction study
		TERRESTRIAL INVERTEBRATES		
Thompson et al. (1995)	[273]	*Anthonomus grandis* (Col. Curculionidae; Boll weevil)	LC^50^ = 1.0 mg L^-1^	Adult mortality; leaf disk 5 days
Thompson et al. (1995)	[273]	*Diabrotica undecimpunctata howardi* (Col. Chrysomelidae; Southern corn rootworm)	LC^50^ = 1.0 µg a.i. per larva	topical – mortality 2-d
McLeod et al. (2002)	[276]	*Epitrix fuscula* (Col. Chrysomelidae; eggplant flea beetle)	2-d LC^50^ = 25.9 mg L^-1^ 2-d LC95 = 208.5 mg L^-1^ 4-d LC^50^ = 9.8 mg L^-1^ 4-d LC95 = 65.4 mg L^-1^	2-d and 4-d mortality; contact with treated leaf disk plant
Lambkin & Rice (2007)	[277]	*Alphitobius diaperinus* (Col. Tenebrionidae)	LC^50^ = 0.037-0.040 µg 100 mL^-1^ LC99 = 0.721-0.809 µg 100 mL^-1^	Contact; solvent acetone
Cisneros et al. (2002)	[310]	*Aleochara bilineata* (Col. Staphylinidae)	10% mortality	Exposition to 200 mg kg^-1^ SPI
Kristensen & Jespersen (2004)	[350]	*Musca domestica* (Diptera)	72 h LC^50^ = 0.51 µg g^-1^	72-h feeding bioassay (µg a.i. per gram of sugar); susceptible population.
Kristensen & Jespersen (2004)	[350]	*Musca domestica* (Diptera)	72 h LC^50^ = 1.5 – 5.5 µg g^-1^	72-h feeding bioassay (a.i. on sugar); resistant population.
Kristensen & Jespersen (2004)	[350]	*Musca domestica* (Diptera)	48 h LC^50^ = 40 ng g fly^-1^	48-h; topical application; susceptible population.
Kristensen & Jespersen (2004)	[350]	*Musca domestica* (Diptera)	48 h LC^50^ = 2.5 – 4.7 ng fly^-1^	48-h; topical application; resistant population.
Salgado (1998)	[4]	*Drosophila melanogaster* (Diptera)	24 h LC^50^ = 8.0 mg kg^-1^	a.i. in 10% sucrose on filter paper; contact and feeding. 24-h exposition
De Deken et al. (2004)	[287]	*Glossina palpalis gambiensis* (Diptera)	LD_50_ = 2.500 mg L^-1^ LD90 = 3.908 mg L^-1^	Topical application SPI on mesonotum of individual flies: 1 µL; 11.6% a.i. µL^-1^. Mortality (absence of the slightest movement) recorded at 48h after application; teneral indiv.
De Deken et al. (2004)	[287]	*Glossina palpalis gambiensis* (Diptera)	LD_50_ = 2.180 mg L^-1^ LD90 = 3.051 mg L^-1^	48-h; topical application 1 µL; 11.6% a.i. µL^-1^; gravid indiv.
De Deken et al. (2004)	[287]	*Glossina morsitans morsitans* (Diptera)	LD_50_ = 1.117 mg L^-1^ LD90 = 1.874 mg L^-1^	48-h; topical application 1 µL; 48% a.i. µL^-1^; gravid indiv.
King & Hennessey (1996)	[278]	*Anastrepha suspensa* (Diptera: Tephritidae)	EC_99_ (f) = 9.4 mg kg^-1^ EC_99_ (m) = 5.8 mg kg^-1^	SPI combined with a sugar-yeast hydrolysate and used as a bait spray on sexually mature females (f) and males (m) in a no-choice test.
Franc & Bouhsira (2009)	[288]	*Ctenocephalides canis* (Siphonaptera: Pulicidae)	24-h mortality: 100% Efficacy from the reinfestation > 99%: 3 wks; 90% 30 d post-treatment	SPI-treated dogs (oral; tablets); 30 mg kg^-1^ l.w.
Blagburn *et al.* (2010)	[289]	*Ctenocephalides felis* (Siphonaptera: Pulicidae)	100% mortality of adult fleas at 4 through 48h post-treatment	SPI-treated dogs (oral); 30 mg kg^-1^ l.w.
Thompson *et al.* (1995)	[273]	*Spodoptera exigua* (Lepid. Noctuidae; Beet armyworm)	2 d LC^50^ = 0.02 µg a.i. per larva	Injection – mortality 2 days; 4th stage
Thompson *et al.* (1995)	[273]	*Spodoptera exigua* (Lepid. Noctuidae; Beet armyworm)	1 d LC^50^ = 0.71 µg a.i. per larva	topical – mortality 1d;&amp;nbsp; 4th stage
Thompson *et al.* (1995)	[273]	*Spodoptera exigua* (Lepid. Noctuidae; Beet armyworm)	5 d LC^50^ = 5.8 mg L^-1^	Diet - mortality 5d; 2nd stage
Thompson *et al.* (1995)	[273]	*Spodoperda frugiperda* (Lepid. Noctuidae; Fall armyworm)	LC^50^ = 3.0 mg L^-1^	Drench
Ahmad *et al.* (2008)	[144]	*Spodoptera litura* (Lepid. Noctuidae)	72 h LC^50^ =1.06 µg mL^-1^ (range 0.87–1.29)	Mortality assessed after 72 h exposure to SPI for a laboratory susceptible population (Lab-PK)
Ahmad *et al.* (2008)	[144]	*Spodoptera litura* (Lepid. Noctuidae)	72 h LC^50^ = 12.1 to 129 mg L^-1^	Mortality assessed after 72 h exposure to SPI. Field populations
Thompson *et al.* (1995)	[273]	*Heliothis virescens* (Lepid. Noctuidae; Tobacco budworm)	24 h LC^50^ = 0.16 – 0.31 mg a.i. L^-1^	Drench – mortality 24h
Crouse *et al.* (2001)	[351]	*Heliothis virescens* (Lepid. Noctuidae; Tobacco budworm)	LC^50^ = 0.31 mg a.i. L^-1^	Neonate larvae; Acute LC^50^ with spinosyn
Crouse *et al.* (2001)	[351]	*Heliothis virescens* (Lepid. Nocrm)	LC^50^ < 0.05 to > 64 mg L^-1^	Neonate larvae; Acute LC^50^ with different spinosyn analogs
Crouse *et al.* (2001)	[351]	*Heliothis virescens* (Lepid. Noctuidae; Tobacco budworm)	4 d LC^50^ = 32 mg L^-1^	A/D mixture (formulated 10b+9j analogs) under simulated field conditions; 4-day residual (cotton)
Crouse *et al.* (2001)	[351]	*Heliothis virescens* (Lepid. Noctuidae; Tobacco budworm)	LC^50^ > 200 mg L^-1^	SPI under simulated field conditions; 4-day residual (cotton)
Salgado (1998)	[4]	*Heliothis virescens* (Lepid. Noctuidae; Tobacco budworm)	LD_50_ = 14 ng (6-31 ng)	LD_50_ value for larvae 50-70 mg; injection.
Thompson *et al.* (1995)	[273]	*Trichoplusia ni* (Lepid. Noctuidae; Cabbage looper)	5 d LC^50^ = 0.08 µg a.i. per larva	topical – mortality 5d; 2^nd^ stage
Thompson *et al.* (1995)	[273]	*Laspeyresia (Cydia) pomonella *(Lep. Tortricidae; Codling moth)	5 d LC^50^ = 0.25 µg a.i. per larva	topical – mortality 5d
Crouse *et al.* (2001)	[351]	*Laspeyresia (Cydia) pomonella* (Lep. Tortricidae; Codling moth)	14 d LC^50^ = 82 mg L^-1^ (acute; eggs) 14 d LC^50^ = 81 mg L^-1^ (larvae)	A/D mixture (formulated 10b+9j analogs) under simulated field conditions; 14-day residual (apple)
Crouse *et al.* (2001)	[351]	*Laspeyresia (Cydia) pomonella* (Lep. Tortricidae; Codling moth)	14 d LC^50^ = 130 mg L^-1^ (acute; eggs) 14 d LC^50^ = 350 mg L^-1^ (larvae)	SPI under simulated field conditions; 14-day residual (apple)
WHO (2008)	[293]	*Apis mellifera* (honey bee)	LD_50_ = 0.057 µg a.i. bee^-1^ (SPI) LD_50_ = 0.049 µg a.i. bee ^-1^ (480SC)	OECD 213. Oral exposure (SPI & spinosyn analog)
WHO (2008)	[293]	*Apis mellifera* (honey bee)	LD_50_ = 0.004 a.i. µg bee ^-1^ (SPI) LD_50_ = 0.050 µg a.i. bee ^-1^ (480SC)	OECD 214. Contact exposure (SPI & spinosyn analog)
WHO (2008)	[293]	*Apis mellifera* (honey bee)	LD_50_ = 0.006 µg a.i. bee ^-1^ (SPI) LD_50_ = 0.049 µg a.i. bee ^-1^ (480SC)	EPPO 170; Acute oral (SPI & spinosyn analog)
Salgado (1998)	[4]	*Periplaneta americana* (cockroach)	24 d LD_50_ = 0.74 µg a.i.	24-d; injection of adult male
Cisneros *et al.* (2002)	[310]	*Doru taeniatum* (earwig)	48% mortality: 1.2 mg kg^-1^ a.i. 98% in the 1200 mg kg^-1^ a.i.	14-d period; contaminated granules
Crouse *et al.* (2001)	[351]	*Aphis gossypii* (Homoptera; Cotton aphid)	LC^50^ = 50 mg L^-1^ (between 42 and 88)	Acute LC^50^; 6 to 8-day-old squash plants infested with cotton aphids 16-24 h prior to treatment
Crouse *et al.* (2001)	[351]	*Aphis gossypii* (Homoptera; Cotton aphid)	LC^50^ = 2.5 to 55 mg L^-1^	Acute LC^50^ with different spinosyn analogs
Thompson *et al.* (1995)	[273]	*Tetranychus urticae* (Two spotted spider mite)	4 d LC^50^ = 5.3 mg L^-1^	Leaf spray – mortality 4 days
Crouse *et al.* (2001)	[351]	*Tetranychus urticae* (Two spotted spider mite)	LC^50^ = 5.3 mg L^-1^	Acute LC^50^; mixed-age mobile mites or mite nymphs transferred to squash plants
Crouse *et al.* (2001)	[351]	*Tetranychus urticae* (Two spotted spider mite)	LC^50^ = 0.4 to 55 mg L^-1^	Acute LC^50^ with different Spinosyn analogs
Crouse *et al.* (2001)	[351]	*Tetranychus urticae* (Two spotted spider mite)	4 d LC^50^ = 25 mg L^-1^	A/D mixture (formulated 10b+9j analogs) under simulated field conditions; 4-day residual (apple)
Crouse *et al.* (2001)	[351]	*Tetranychus urticae* (Two spotted spider mite)	4 d LC^50^ > 100 mg L^-1^	SPI under simulated field conditions; 4-day residual (apple)
WHO (2008)	[293]	*Eisenia foetida* (earthworm)	14 d LC^50^ > 970 mg kg^-1^ d.s.	14-d, 20 ± 2°C; Acute toxicity
[Dow AgroSciences] (2010)	[308]	Earthworm	NOEC = 18.65 mg kg^-1^ d.s.	Reproductive effects; kg soil
		AQUATIC INVERTEBRATES		
WHO (2008)	[293]	*Daphnia magna* (water flea)	48 h EC_50_ > 1.0 mg L^-1^	48-h, FIFRA 72-2 & OECD 202 Part 1 (20 ± 2°C); Acute toxicity, static
WHO (2008)	[293]	*Daphnia magna* (water flea)	48 h EC_50_ = 9.1 mg L^-1^	48-h, OECD 202 Part 1 (20 ± 2°C), Acute toxicity, static, formulation 480SC
WHO (2008)	[293]	*Daphnia magna* (water flea)	21 d NOEC = 0.0012 mg L^-1^ (flow through); 21 d NOEC = 0.0080 mg L^-1^ (semi-static)	21-d, FIFRA 72-4 & OECD 202 Part 2 (20 ± 2°C); Chronic toxicity
[Dow AgroSciences] (2010)	[308]	*Americamysis bahia* (Mysid shrimp)	48 h LC^50^ > 7.9 mg L^-1^	48-h
[Dow AgroSciences] (2010)	[308]	*Palaeomonetes pugio* (Grass shrimp)	48 h LC^50^ > 9.7 mg L^-1^	48-h
Bond *et al* (2004)	[285]	*Anopheles albimanus* (Diptera)	LC^50^ = 0.024 mg L^-1^	Suspension concentrate formulation of SPI; 24h lethal concentration for third and fourth instars
Bond *et al* (2004)	[285]	*Aedes aegypti* (Diptera)	LC^50^ = 0.025 mg L^-1^	Suspension concentrate formulation of SPI; 24h lethal concentration for third and fourth instars
Darriet & Corbel (2006)	[283]	*Aedes aegypti* (Diptera)	LC^50^ = 0.055 mg L^-1^ LC95 = 0.200 mg L^-1^	Larval mortality
Antonio *et al.* (2009)	[352]	*Aedes aegypti* (Diptera)	LC^50^ = 0.06 mg L^-1^	late third instars, wild population
WHO (2008)	[293]	*Chironomus riparius* (midge)	25 d NOEC = 0.002 mg L^-1^	25-d, OECD 219 (20 ± 0.5°C); Chronic toxicity, static
[Dow AgroSciences] (2010)	[308]	*Chironomus riparius* (midge)	NOEC = 0.375 µg L^-1^	Chronic toxicity
[Dow AgroSciences] (2010)	[308]	*Crassostrea virginica* (Eastern Oyster)	96 h EC_50_ = 0.3 mg L^-1^	96-h
		ALGAE AND PLANTS		
WHO (2008)	[293]	*Navicula pelliculosa* (Freshwater green algae)	120 h EC_50_ = 0.079 mg L^-1^	120 h, FIFRA 123-2 & OECD 201 (22 ± 1°C); Static water
WHO (2008)	[293]	*Navicula pelliculosa* (Freshwater green algae)	120 h EC_50_ = 0.35 mg L^-1^	120 h, OECD 201 (22 ± 1°C); Static water; formulation 480SC
WHO (2008)	[293]	*Anabaena flos-aquae* (Blue green algae)	120 h EC_50_ = 6.1 mg L^-1^	120 h, FIFRA 123-2 & OECD 201 (24 ± 2°C); Static water
WHO (2008)	[293]	*Selenastrum capricornutum* (green algae)	72 h EC_50_ = 56 mg L^-1^	72-h, FIFRA 123-2 & OECD 201 (24 ± 2°C); Static water
[Dow AgroSciences] (2010)	[308]	*Selenastrum capricornutum* (green algae)	7 d EC_50_ > 105.5 mg L^-1^	7-d
[Dow AgroSciences] (2010)	[308]	*Skeletonema costatum* (Marine diatom)	5 d EC_50_ = 0.23 mg L^-1^	5-d
WHO (2008)	[293]	*Lemna gibba* (duckweed)	14 d EC_50_ = 6.6 mg L^-1^	14-d, FIFRA 123-2 & OECD 221 (25.3 ± 0.15°C); Static water
		MICRO-ORGANISMS		
[Dow AgroSciences] (2010)	[308]	Micro-organisms	Soil: no effect at 7 mg L^-1^ Sewage: no effect at 100 mg L^-1^	

**Table 9.** Ecotoxicity of MLs on the Aquatic and Terrestrial Ecosystems and the Whole Dung Community (for Key to Dosages, see Table Footnote)

Source	References	Compound	Whole dung community	Toxicity / response	Conditions
Floate *et al.* (2002)	[322]	DOR	Coprophilous species of Coleoptera, Diptera and Hymenoptera	Reduced numbers of insects developing in dung voided 2 (for 1 species) and 4 (for 8 species) weeks post-treatment; some evidence of delayed degradation	Pour-on (cattle)
Suarez *et al.* (2003)	[353]	DOR	Coprophilous arthropods and nematodes	Reduced numbers of beetle and fly larvae, springtails, mites and nematodes; no effect on degradation	Injectable (cattle)
Floate *et al.* (2008)	[323]	DOR	Coprophilous species of Coleoptera, Diptera and Hymenoptera	Reduced emergence of some species in dung voided ≤ 16 weeks post-treatment	Pour-on (cattle)
Suarez *et al.* (2009)	[234]	DOR	Coprophilous arthropods and nematodes	Reduced numbers of beetle and fly larvae, springtails, mites and nematodes; no effect on degradation	Injectable (cattle)
Floate *et al.* (2002)	[322]	EPR	Coprophilous species of Coleoptera, Diptera and Hymenoptera	Reduced numbers of insects developing in dung voided 1 (for 3 species), 2 (for 4 species) and 4 (for 2 species) weeks post-treatment; some evidence of delayed degradation	Pour-on (cattle)
McCracken & Foster (1993)	[354]	IVM	Dung and soil invertebrates	Significant differences in community in and under pats	Injectable (cattle) Diversity
Barth *et al.* (1993)	[346]	IVM	Dung degradation; dipteran, coleopteran and nematode diversity	Decrease in number of larvae; no effect on number of adults	Slow release bolus 12 mg day^-1^ for 120 days (cattle).
Floate (1998)	[335]	IVM	Coprophilous species of Coleoptera, Diptera and Hymenoptera	Reduced emergence of some species in dung voided ≤ 12 weeks post-treatment	Pour-on (cattle)
Krüger & Scholtz (1998)	[355]	IVM	Coprophilous species of Coleoptera, Diptera	Lower in treated and natural pats after 3 months and after 2 months in artificial pats	Injectable (cattle) 3 months; evenness and diversity; drought conditions
Krüger & Scholtz (1998)	[356]	IVM	Coprophilous species of Coleoptera, Diptera	Natural pats: reduced diversity first 7 days post-treatment and 3 months post-treatment	Injectable (cattle) 3 months evenness and diversity; high-rainfall conditions
Madsen *et al.* (1990)	[177]	IVM	Coprophilous species of Coleoptera, and Diptera	Inhibited development of Cyclorrhapha dipterans for 30 days, Nematocera dipterans for 10 days; no effect on earthworms	Injectable (cattle) 30 days development
Sommer & Bibby (2002)	[357]	IVM	Decomposition of dung organic matter in soil	reduced loss for all time intervals, i.e. for 0-8, 0-12 and 0-16 weeks	Single subcutaneous injection to heifers of 10 mg 50 kg^-1^ b.w.
Floate *et al.* (2002)	[322]	IVM	Coprophilous species of Coleoptera, Diptera and Hymenoptera	Reduced numbers of insects developing in dung voided 1 (for 2 species), 2 (for 4 species) and 4 (for 6 species) weeks post-treatment	Pour-on (cattle)
Dimander *et al.* (2003)	[358]	IVM	No observations on dung fauna	No observed effect on dung degradation	SR bolus with 12 mg day-1 (40-65 µg kg-1 b.w. day-1) for 135 days (cattle)
Jacobs *et al.* (1988)	[359]	IVM	No observations on dung fauna	No observed effect on dung degradation	Pour-on (cattle)
Wall & Strong (1987), Strong & Wall (1988)	[202, 360]	IVM	Coprophilous arthropods	Reduced numbers of beetles and flies in dung voided ≤ 100 days post-treatment; reduced dung degradation; no effect on colonization with spiked dung	SR bolus with 40 µg.kg^-1^ b.w. day^-1^) for 135 days (cattle); spiked dung with 0.5 and 0.125 mg kg^-1^ dung f.w.
McKeand *et al.* (1988)	[361]	IVM	No observations on dung fauna	No observed effect on dung degradation	Pour-on (cattle)
Schaper & Liebisch (1991)	[181]	IVM	Coprophilous arthropods, nematodes	Reduced emergence of adult Diptera (Sepsidae, Muscidae); reduced numbers of Dipteran larvae and nematodes; no effect on numbers of adult or larval dung beetles; no observed effect on dung degradation	Injectable (cattle)
Herd *et al.* (1993)	[362]	IVM	No observations on dung fauna	Delayed degradation of copromes (dung)	Oral suspension (horses) 200 µg kg^-1^
Wratten *et al.* (1993)	[203]	IVM	Earthworms	No observed effect on dung degradation; no effect on numbers of earthworms recovered from soil near and under dung	SR bolus with 0.05-0.08 mg.kg^-1^ b.w.day^-1^) for 90 or 120 days (cattle); Injectable (cattle)
Nilssen *et al.* (1999)	[363]	IVM	Coprophilous arthropods, nematodes	Reduced numbers of nematodes; no effect on degradation	Injectable (reindeer)
Suarez *et al.* (2003)	[234]	IVM	Coprophilous arthropods and nematodes	Reduced numbers of beetle and fly larvae, springtails, mites and nematodes; no effect on degradation	Injectable (cattle)
Iglesias *et al.* (2006)	[364]	IVM	Coprophilous arthropods	Reduced number and diversity of arthropods	Injectable (cattle)
Iwasa *et al.* (2005)	[331]	IVM	Coprophilous Diptera	Reduced numbers of cyclorrhaphan flies (7 taxa); increased numbers of nematoceran flies (Ceratopogonidae, Psychodidae)	Pour-on (cattle)
Römbke *et al.* (2010)	[54]	IVM	Dung community: beetles (species level), flies (total number) Soil organisms: Collembola, mites	Dung beetle: Volinus distinctus, NOEC and (EC_50_): 0.50 and 0.62mg IVM kg^-1^ dung d.w., respectively. Dung fly larvae: NOEC <0.31mg IVM kg^-1^ d.w. Dung decomposition: NOEC <0.78mg IVM kg^-1^ dung d.w. No IVM-related effects on collembolans and mites.	Injectable (cattle); residue analysis in dung: 0.31 to 0.81mg IVM kg^-1^ dung d.w.; in soil: concentrations much lower
Floate *et al.* (2002)	[322]	MOX	Coprophilous species of Coleoptera, Diptera and Hymenoptera	Reduced numbers of insects developing in dung voided 1 (for 5 species) and 2 (for 1 species) weeks post-treatment; some evidence of delayed degradation	Pour-on (cattle)
Iwasa *et al.* (2008)	[266]	MOX	Coprophilous Diptera	Fewer Sepsis latiforceps, Sphaeroceridae and Sciaridae	Pour-on (cattle)
Suarez *et al.* (2009)	[234]	MOX	Coprophilous arthropods and nematodes	Reduced numbers of beetle and fly larvae, springtails, mites and nematodes; no effect on degradation	Injectable and Pour-on formulations (cattle)
Brinke *et al.* (2010)	[213]	IVM	Benthic communities Natural sediments and overlying water (224 d)	Meio fauna community: 224 d NOEC = 6.2 µg/kg sedim. dry wt Nematodes community: 224 d NOEC = 0.6 µg/kg sediment dry wt	abundance and community composition
Sanderson *et al.* (1997)	[56]	IVM	Cladoceran community	10-97 d NOEC, species richness < 30 ng L^-1^ Significant effects were observed at the lowest nominal concentration (30 ng L^-1^)	Aquatic mesocosm (265 d). Abundance and species richness

## ABBREVIATIONS

ABM: = Abamectin
a.i.: = Active ingredient
ACR: = Acute to chronic ratio between LC_50_ and NOEC
b.w.: = Body weight
ca.: = About
CarE: = carboxylesterase
d.: = Day
d.f.: = Dry faeces
d.s.: = Dry soil
d.w.: = Dry weight
DEM: = Diethyl maleate
DOTTS: = Dung Organism Toxicity Test Standardization (http://www.dottsgroup.org). It is an advisory group of the Society for Environmental Toxicity and Chemistry (SETAC).
DOR: = Doramectin
*e.g*.: = For example
EC: = Effective concentration
EC_50_: = Half maximum effective concentration (refers to the concentration of a drug which induces a response halfway between the baseline and maximum)
EC_80_: = 80% effective concentration
EFSA: = European Food Safety Agency
EMB: = Emamectin benzoate
EMA: = European Medicines Agency
EPPO: = European Plant Protection Organisation
EPR: = Eprinomectin
ERA: = Environmental Risk Assessment
*et al.*: = and others
EU: = European Union
f.: = Female
f.w.: = Fresh weight
FC: = Faecal concentration
GABA: = Gamma-aminobutyric acid
GST: = Glutathione S-transferase
IC_50_: = Median Inhibition Concentration (concentration that reduces the effect by 50%)
IM: = Intramuscular
IOBC: = International Organization for Biological Control
IPM: = Integrated pest management
ISO: = International Organisation for Standardisation
IVM: = Ivermectin
LC_50_: = Lethal concentration 50%
LD_50_: = Lethal dose 50%
LOEC: = Lowest observed effect concentration
LOEL: = Lowest observed effect level
l.w.: = Live weight
m.: = Male
MATC: = Maximum Acceptable Toxicant Concentration
MFO: = Mixed function oxidase
mg: = Milligram
µg: = Microgram
µg L^-1^: = Microgram per litre
MATC: = Maximum Acceptable Toxicant Concentration
MIC: = Minimal inhibitory concentration
ML: = Macrocyclic lactone
MSD: = Merck Sharp & Dohme
ng: = Nanogram
NOAEL: = No observed adverse effect level
NOEC: = No observed effect concentration
NRA: = National Registration Authority for Agricultural and Veterinary Chemicals
OECD: = Organisation for Economic Co-Operation and Development
PBO: = Piperonyl butoxide
PEC: = Predicted Environmental Concentration
pg: = Picogram
ppb: = Parts per billion
ppm: = Parts per million
SD: = Standard Deviation
SETAC: = Society for Environmental Toxicity and Chemistry
SR: = Slow release
TOC: = Total Organic Carbon
TPP: = Triphenyl phosphate
VICH: = International Cooperation on Harmonisation of Technical Requirements for Registration of Veterinary Medicinal Products (http://www.vichsec.org/)
VMP: = Veterinary Medicinal Product
w.w.: = Wet weight
